# Eight new species of *Deltochilum* Eschscholtz, 1822 (subgenus *D. Deltohyboma* Lane, 1946) from the Eastern Cordillera of Colombia and Venezuelan Andes (Coleoptera, Scarabaeinae, *plebejum* species group)

**DOI:** 10.3897/zookeys.1280.182114

**Published:** 2026-05-22

**Authors:** Arturo González-Alvarado, Jhon Cesar Neita

**Affiliations:** 1 Postdoctoral research, Instituto de Investigación de Recursos Biológicos Alexander von Humboldt, Villa de Leyva, Boyacá, Colombia Postdoctoral research, Instituto de Investigación de Recursos Biológicos Alexander von Humboldt Villa de Leyva, Boyacá Colombia; 2 Curator of Entomology and Invertebrate Collections, Instituto de Investigación de Recursos Biológicos Alexander von Humboldt, Villa de Leyva, Boyacá, Colombia Curator of Entomology and Invertebrate Collections, Instituto de Investigación de Recursos Biológicos Alexander von Humboldt Villa de Leyva, Boyacá Colombia

**Keywords:** Deltochilini, dung beetles, Mérida Cordillera, new species, Neotropical, Sierra Nevada de Santa Marta, South America

## Abstract

The Neotropical genus *Deltochilum* Eschscholtz, 1822, is one of the most speciose genera in the tribe Deltochilini. This genus of dung beetles (Scarabaeidae: Scarabaeinae) is divided into eight subgenera. The subgenus *D. Deltohyboma* Lane, 1946, with 51 valid species, is the most diverse in the genus and was recently divided into 19 species groups. This paper presents the description of eight new species from the plebejum species group: *Deltochilum tyba***sp. nov**., *D. pseudoabdominale***sp. nov**., *D. parapseudoabdominale***sp. nov**., *D. picachos***sp. nov**., *D. pauxi***sp. nov**., *D. altiventris***sp. nov**., *D. ventripuncturatus***sp. nov**., and *D. nobile***sp. nov**. These species are distributed along the Eastern Cordillera of Colombia and the Venezuelan Andes, with one species also found in the Sierra Nevada de Santa Marta (Colombia). The lectotype for *D. plebejum* Balthasar, 1939 is designated herein. Three identification keys (for major males, minor and medium-sized males, and females), along with descriptions, photographs, and distribution maps are presented for all species. Additionally, redescriptions of *D. plebejum* and *D. abdominale* Martínez, 1947, originally included in this species group, are presented. With these new species, the number of valid species in the subgenus *D. Deltohyboma* reaches 59.

## Introduction

The Neotropical genus *Deltochilum* is divided into eight subgenera. The subgenus *D. Deltohyboma* was recently divided into 19 species groups (González-Alvarado and Vaz-de-Mello 2021). One of them, the plebejum species group, was defined by the following character combination: anterior margin of the clypeus, between clypeal teeth, concave, slightly expanded, but not triangular in shape; posterior edge of metafemur with one margin; ventral surface of protibia with carina or tubercles; humeral region of the elytra with two carinae; narrow striae and elongate tubercle on interstria III at elytral apex (González-Alvarado and Vaz-de-Mello 2021).

Before this work, only two species were recognized in this group. One, *Deltochilum plebejum*, was described in 1939 by Balthasar using two specimens from the Maracaibo Basin in Venezuela. However, the exact locality remains unknown, and no additional information or new records for the species have been reported since that time.

The other described species, *D. abdominale*, was originally described from Cerro Naiguatá, Venezuela (Martínez 1947) and later cited for Colombia by Vulcano and Pereira (1964). However, the subsequent record from Guaviare, Colombia (Medina et al. 2001), is likely a misidentification. This is supported by the fact that Colombian species in this group appear restricted to the Eastern Cordillera and its adjacent piedmont, typically occurring at elevations between 400 m and 2000 m.

Specimens of this species group are rare in collections, and individuals are typically collected in low numbers at any given locality. Herein, eight new species are described, and a lectotype is designated for *D. plebejum*.

## Materials and methods

The terms of the external morphology follow González-Alvarado and Vaz-de-Mello (2021). Following Lawrence et al. (2010), we use the terms metaventrite and metaventral process instead of metasternum, and ventrites for the visible abdominal sternites. Terminology for integumental sculpturing (e.g., the shiny points on the interstriae) follows González-Alvarado and Vaz-de-Mello (2021). The term stria refers specifically to the line connecting the strial punctures. Morphometric proportions were calculated using the following ratios:

1. Interocular distance: the width of one eye was measured at its widest point. The interocular distance was then measured from the internal margin of one eye to the internal margin of the other at the same level.
2. Strial width vs. Interstrial width: strial width was measured on the second stria at the elytral disc. The distance was then measured from the external margin of that stria to the internal margin of the third stria.
3. Interstrial punctures size: the diameter of a single puncture was measured on the third interstria at the elytral disc. This was compared to the distance from the external margin of the second stria to the internal margin of the third stria at the same point.
4. Pygidial punctures size: The diameter of a single puncture was measured on the pygidial disc. This was compared to the total distance between the internal margins of the pygidium at that same point.

Names of the structures of the endophallus are based on Medina et al. (2013), Génier (2019), Cristóvão and Vaz-de-Mello (2020) and González-Alvarado and Vaz-de-Mello (2021). For practical purposes, the two endophallites of the medial region of the endophallus are named “right” and “left” endophallites according to their position when the endophallus is everted, with the plate-shaped endophallite upwards (dorsal) and the basal endophallite ventrally (González-Alvarado and Vaz-de-Mello 2021).

The Material examined section was prepared using AUTOMATEX (Brown 2013). Labels for the type material of previously described species are transcribed verbatim. For labels without geographic coordinates, those were searched for using the GEOLocate Web Application https://www.geo-locate.org/web/WebGeoref.aspx, Google Earth. The CMNC specimens were georeferenced by François Génier. The georeferenced geographic coordinates and the interpreted information (e.g., Mag. [Magdalena]) are given within square brackets “[ ]”. A question mark “?” was added to geographic coordinates or any other information deemed uncertain.

The distribution map was constructed using QGIS 3.40 software, using the following shapefiles: country and administrative division (http://www.gadm.org) and Google Satellite layer. The figures were prepared using Inkscape 1.4 software.

This work is based on the study of 371 specimens from the following collections (curator(s) in parentheses).

**BDGC** Bruce D. Gill personal collection, Ontario, Canada (Bruce Gill).

**CALT-ECC** Colección Alejandro Lopera Toro-Escarabajos Coprófagos de Colombia, Villa de Leyva, Boyacá Colombia (Alejandro Lopera - Arturo González).

**CBUMAG** Colecciones Biológicas de la Universidad del Magdalena (Larry Jiménez).

**CEMT** Coleção Entomológica de Mato Grosso Eurides Furtado, Universidade Federal de Mato Grosso, Cuiabá, Brazil (Fernando Z. Vaz-de-Mello).

**CMNC** Canadian Museum of Nature, Ottawa, Canada (François Génier).

**IAvH** Colección Entomológica del Instituto de Investigación de Recursos Biológicos Alexander von Humboldt, Villa de Leyva, Colombia (Jhon César Neita).

**ICN** Colección Entomológica del Instituto de Ciencias Naturales, Universidad Nacional de Colombia, Bogotá, Colombia (Fernando Fernández – Juan Pablo Botero).

**NMPC** National Museum (Natural History), Prague, Czech Republic (Jiří Hájek).

**MANC** Museo Argentino de Ciencias Naturales “Bernardino Rivadavia”, Buenos Aires, Argentina (Arturo Roig).

**RMNH** National Museum of Natural History, Leiden, Netherlands (Max Caspers - Oscar Vorst).

**UPTC-CE** Colección Entomológica del Museo de Historia Natural “Luis Gonzalo Andrade,” Tunja, Boyacá, Colombia (Juan Carvajal - Irina Morales).

**Figure 1.**
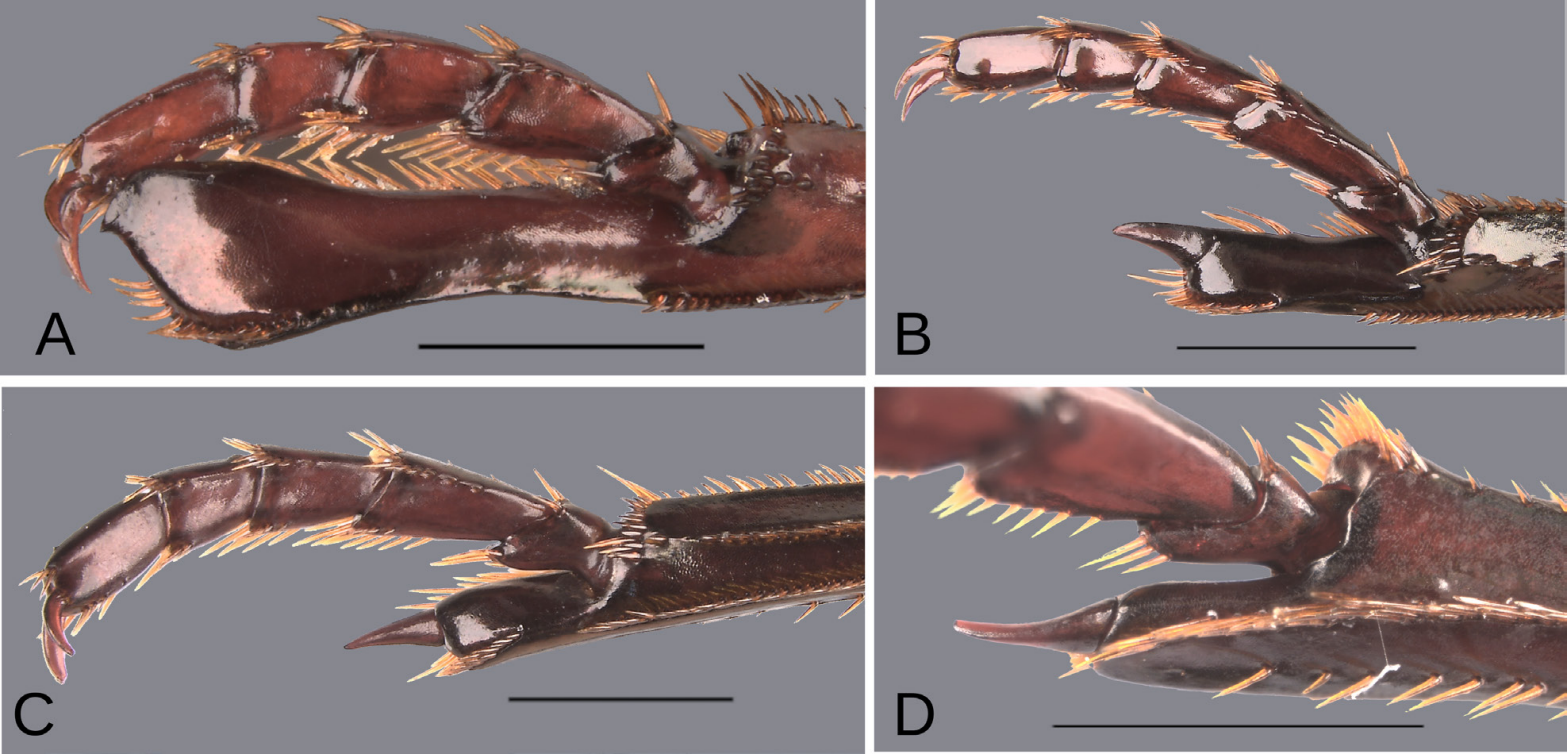
Metatibial apex of the three “sizes” of males of the plebejum species group. **A**. Major male, paratype of *Deltochilum ventripuncturatus* sp. nov.; **B**. Major male, paratype of *D. pseudoabdominale* sp. nov.; **C**. Medium-sized male, holotype of *D. pauxi* sp. nov.; **D**. Minor male, paratype of *D. picachos* sp. nov. Scale bars: 1 mm.

As the species of the plebejum species group exhibit allometric male dimorphism (see Results and discussion section), three artificial size categories of males are used to facilitate species description and identification. These three categories are:

1. Major: males in which the metatibial spur insertion is elongated (at least the length of the two basal tarsomeres) and the metatibial spur is fused (Fig. 1A, B).
2. Medium-sized: males in which the metatibial spur insertion is elongated (longer than the basal tarsomere) and the metatibial spur is articulated (Fig. 1C).
3. Minor: males with no, or poorly, elongated spur insertion (approximately the length of the basal tarsomere) and an articulated metatibial spur (Fig. 1D).

Based on these size categories, two separate identification keys were constructed: one for major males and another for medium-sized and minor males. The first provides the easiest and most reliable method for species identification. A third identification key was also created for females (though it can be used for males as well). However, we recommended that, whenever possible, the identification key for major males should be used, because males possess the main characters used for species definition, and the females of some species are very similar.

## Results and discussion

**Figure 2.**
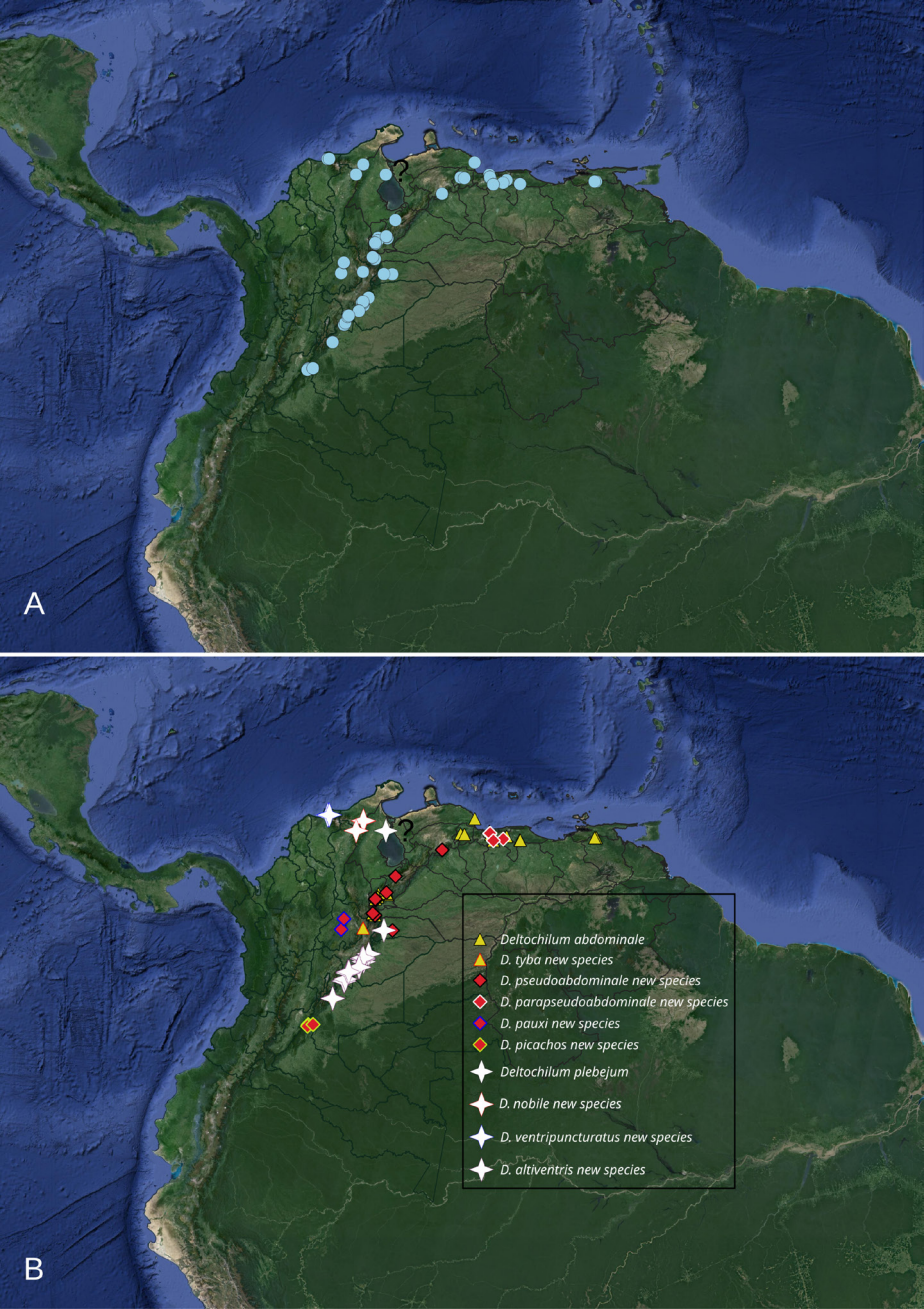
Known distribution of the species of the plebejum species group. **A**. Map showing all records used in this work; **B**. Map showing record for each species, grouped (same symbol) by related species. The question mark “?” indicate the unspecified type locality (Maracaibo Basin) of *Deltochilum plebejum* Balthasar, 1939.

With the new species described here the number of valid species of *Deltochilum* rises to 123 (without taking into account the subspecies) and the subgenus *D. Deltohyboma* to 59. The plebejum species group comprises ten species. All species were found distributed along the Eastern Cordillera of the Andes, Mérida Cordillera, and the Sierra Nevada de Santa Marta. The known range extends from the southernmost locality at Picachos, Colombia, north to the Sierra Nevada de Santa Marta, representing the northeast collection point, with the most northeastern point being Teresén, Monagas, Venezuela (Fig. 2A).

With the information provided in the specimen labels, the species seems to be associated with well-preserved forests, and very few specimens were collected in degraded habitats and pastures. A clear association with dung or carrion cannot be made: the specimens were collected with those two types of bait, and it is possible that the species are generalist. Unfortunately, as the majority of the species in this species group are new, no ecological information has been published and further information is unlikely to elucidate where these species can be collected.

As stated by González-Alvarado and Vaz-de-Mello (2021) this species group is closely related with the *parile* and *aequinoctiale* species groups. These three species groups share several characters and are distributed in the northeast part of the Andes. In some localities species from the plebejum species group are found in sympatry with species from the *aequinoctiale* and/or *parile* species groups (González-Alvarado and Vaz-de-Mello 2021). While some species in this group appear to be geographically isolated, others occur in sympatry (Fig. 2B). For instance, *Deltochilum pseudoabdominale* sp. nov. has been collected alongside *D. abdominale* at certain sites, and with *D. parapseudoabdominale* sp. nov. at others. Similarly, *D. altiventris* sp. nov. was found co-occurring with *D. pseudoabdominale* sp. nov. in one locality and with *D. picachos* sp. nov. in another. The presence of several unique morphological characters in these overlapping populations provides strong evidence to support the hypothesis that they represent distinct species.

The sexual dimorphism present in the plebejum species group is found in several structures. Two of them, namely the protibial spur (broad and foliaceus in males, spiniform in females) and the first ventrite, expanded in males, whereas it is not expanded in females, are not affected by allometry. On the other hand, other structures are affected by allometry in males, these structures and their allometry are: 1. first ventrite elevated, the size of that elevation varies; 2. insertion of the metatibial spur, that insertion elongated or not, with the spur articulated or fused; 3. internal carina of the metatibia elevated, the size of the elevation and the shape varies; 4. mesofemur with denticle or protuberance, the size of that denticle or the protuberance varies in size; 5. metafemur and metatibia are curved; the curve varies from poorly curved to strongly curved. These aforementioned allometric characters are not present in all species. Instead, each species presents a specific combination of those traits, some of which are shared across different species. We hypothesized that species sharing specific combination of these dimorphisms are more closely related to one another than to those lacking them.

The discrete division of three sizes of males used here (see Materials and methods section) is artificial because the sizes of the structures affected by allometry are continuous. However, these divisions are considered useful for the species description and identification. Moreover, the combination of these allometric characters is considered relevant to species delimitation. The fitness and/or the use of these allometric variations in reproduction is unknown, and this is an interesting theme of research. This is not the only species group of the subgenus *D. Deltohyboma* that presents such allometric sexual dimorphism, and its function is unknown for any group (González-Alvarado and Vaz-de-Mello 2021).

*Deltochilum plebejum*, *D. nobile* sp. nov., *D. ventripuncturatus* sp. nov., and *D. altiventris* sp. nov. are considered closely related, sharing the following combination of characters: mesofemur lacking a denticle; the elevation of the internal carina of the metatibia is formed by tubercles basally and a carina medially; the first ventrite is elevated; and generally, the striae are thin. *Deltochilum plebejum* and *D. nobile* sp. nov. are considered closely related; however, *D. plebejum* is only known from an unspecific locality (Maracaibo Basin). It is possible that the northernmost part of the Eastern Cordillera also delimits its distribution, with *D. plebejum* on the eastern slope and *D. nobile* sp. nov. on the western slope (Fig. 2B, white stars).

Meanwhile, *D. ventripuncturatus* sp. nov., and *D. altiventris* sp. nov. appear to be species geographically isolated from each other and from the other two species. *Deltochilum ventripuncturatus* sp. nov. is found in the Sierra Nevada de Santa Marta, while *D. altiventris* sp. nov. is distributed on the eastern slope of the Eastern Cordillera, much further south than *D. plebejum* and *D. nobile* sp. nov. It is possible that the point where the Eastern Cordillera bifurcates (The Santurbán Massif) and forms the Venezuelan Andes marks the northernmost distribution of *D. altiventris* sp. nov. (Fig. 2B, white stars).

The other species, *D. pseudoabdominale* sp. nov., *D. parapseudoabdominale* sp. nov., *D. picachos* sp. nov., and *D. pauxi* sp. nov., share the following combination of characters: mesofemur lacking a denticle; the elevation of the internal carina of the metatibia is formed by small tubercles or a small carina basally and normally a small carina medially; the first ventrite is not elevated; and generally, the striae are wide. These four species appear to be geographically isolated latitudinally within the Eastern Cordillera and the Venezuelan Andes and/or are excluded by other species of the plebejum species group (Fig. 2B, red diamonds). *Deltochilum pseudoabdominale* sp. nov., *D. parapseudoabdominale* sp. nov. could be excluded by *D. abdominale* in the northernmost part of the Venezuelan Andes. Meanwhile, *D. pseudoabdominale* sp. nov. and *D. picachos* sp. nov. could be excluded by *D. altiventris* sp. nov. in the center of the Eastern Cordillera (Fig. 2B, red diamonds).

*Deltochilum abdominale* and *D. tyba* sp. nov. both exhibit a denticle on the mesofemur, possess thin striae, and the elevation of the internal carina of the metatibia is formed by tubercles. However, *D. tyba* sp. nov. has the first ventrite elevated, while *D. abdominale* does not. This shared morphology, combined with the difference in the first ventrite, makes it difficult to associate these species with others. Considering only the first ventrite, *D. tyba* sp. nov. is allied with *Deltochilum plebejum*, *D. nobile* sp. nov., *D. ventripuncturatus* sp. nov., and *D. altiventris* sp. nov., while *Deltochilum abdominale* is allied with *D. pseudoabdominale* sp. nov., *D. parapseudoabdominale* sp. nov., *D. picachos* sp. nov., and *D. pauxi* sp. nov. However, we consider *D. abdominale* and *D. tyba* sp. nov. closely related because they bear a denticle on the mesofemur and because of their distribution. It appears that the Eastern Cordillera is delimiting their distribution, with *D. abdominale* distributed on the eastern slope and *D. tyba* sp. nov. on the western slope (Fig. 2B, yellow triangles).

These hypotheses need to be evaluated with phylogenetic analyses, and studies of genetics and ecology. This species group could be an interesting group for evolutionary and speciation studies.

### The plebejum species group

**Figure 3.**
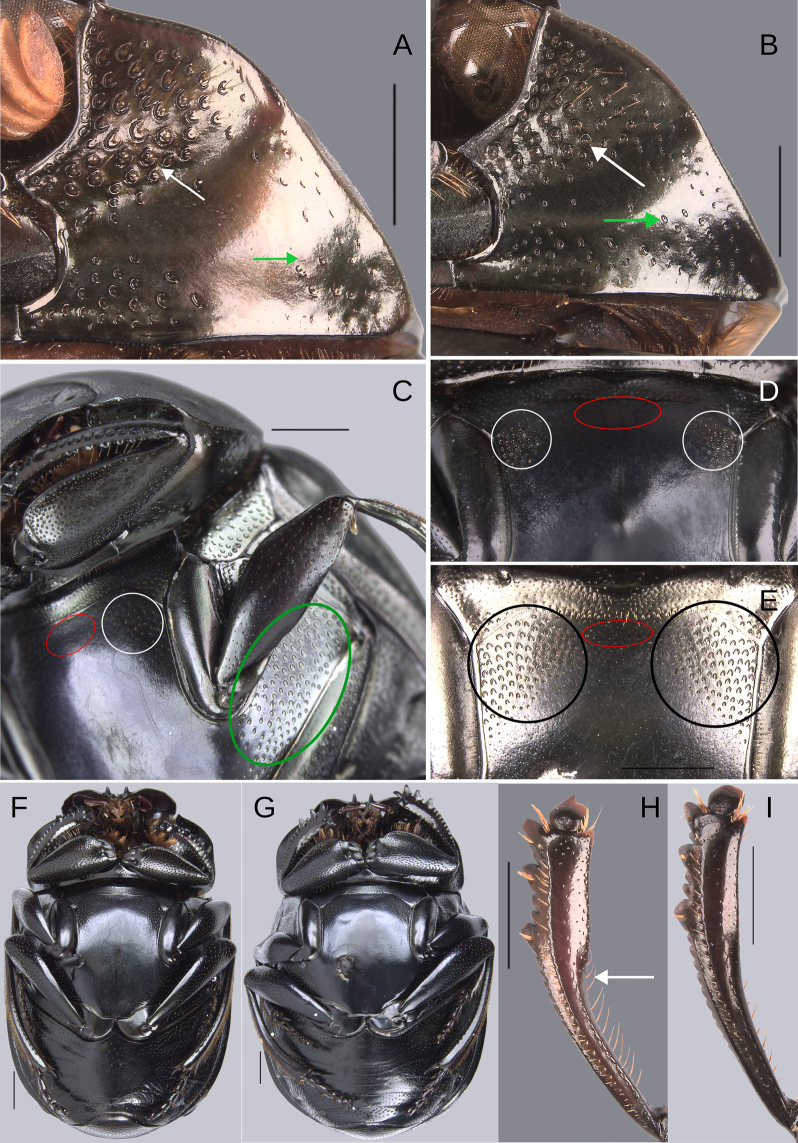
General morphology of the species of the plebejum species group. Circles showing the punctures of the anterolateral (white and black circles) and anterocentral (red circle) areas of the metaventral process. Green circle showing the punctures of the lateral lobes of metaventrite. **A**. Hypomera, paratype of *Deltochilum altiventris* sp. nov., arrows showing the incomplete closed anterior (white arrow) and posterior (green arrow) punctures; **B**. Hypomera, *D. abdominale* Martínez, 1947, white arrow showing the complete closed anterior punctures and the green arrow showing the incomplete closed posterior punctures; **C**. Metaventrite, paratype of *D. altiventris* sp. nov.; **D**. Anterior part of the metaventral process, holotype of *D. picachos* sp. nov.; **E**. Anterior part of the metaventral process, holotype of *D. pauxi* sp. nov.; **F**. Male ventral view, paratype of *D. parapseudoabdominale* sp. nov.; **G**. Female ventral view, *D. abdominale* Martínez, 1947; **H**. Male protibia, paratype of *D. altiventris* sp. nov., white arrow showing the strongly broadening toward the apex; **I**. Male protibia, holotype of *D. pauxi* sp. nov. Scale bars: 1 mm.

A diagnosis and description of this species group is provided by González-Alvarado and Vaz-de-Mello (2021). With the new species recognized herein, certain characters are added or modified from that description as follows; only these supplemental or amended characters are treated here:

**Redescription**. Eyes large, inter-ocular distance 8–10× the width of one eye. Hypomera with punctures incompletely closed and setigerous (Fig. 3A, white arrow, green arrow) or ocellated and setigerous punctures on the anterior part (Fig. 3B, white arrow) and most of the punctures incompletely closed posteriorly and without setae (Fig. 3B, green arrow). Tubercles at elytral apex on interstriae with following variations: 1) III, V–VII with all tubercles well developed. 2) III–VII with all tubercles well developed or with IV poorly developed. 3) II–VII with all tubercles well developed. 4) III, V–VII with tubercles, with I and IV with a small elevation, those elevations have the same brightness as the tubercles. Striae almost inconspicuous or narrow, width of third stria, in species with the broadest striae, 1/16 of the distance between striae II and III. Metaventral process with anterior-lateral areas bearing punctures (Fig. 3C, white circle), those punctures larger than the punctures in the anterior-central area (Fig. 3C, red oval) and varying in shape, size, and density between species (Fig. 3D, E, white circle, black circle, red oval). Lateral lobes of metaventrite with largest punctures (Fig. 3C, green oval), varying in shape, size, and density between species.

**Sexual dimorphism. Male** (Fig. 3F). Protibial spur broad and foliaceus. Protibia strongly (Fig. 3H) or regularly (Fig. 3I) broadened toward the apex. Mesofemur unarmed or with either a denticle or a protuberance. Apex of mesotibia on ventral-internal margin with a large spatulate expansion. Metatrochanter with a dorsal hook-shaped protrusion. Metafemur is more curved than female, in some species males it is strongly broadened toward the apex (see Fig. 21H). Internal carina of metatibia more elevated. The internal margin of the metatibia in females has a minimally elevated carina bordered by setigerous punctures, almost equidistantly separated. In males that carina is strongly elevated, mainly on the basal third, and has much less setigerous punctures on the basal middle. In some species the basal setigerous punctures, and depending on the view, give the carina the appearance of tubercles. Also, in some species, that carina after the basal third is abruptly reduced (much less elevated), and after that reduction the carina becomes abruptly larger (this gives the appearance that the metatibia has two carinae, or basal tubercles and carina). Ventrite I expanded posteriorly, expansion reaching the fourth or fifth ventrite; width of the expansion of ventrite I, on ventrite III, from narrower (see Fig. 14E, white lines) to as wide as (see Fig. 12E, white lines) the distance between mesocoxae. In some species, ventrite I is elevated or elevated and with small punctures. Medial area of endophallus one or two endophallites, if with two, the left endophallite 4–7× smaller than right endophallite.

**Female** (Fig. 3G). Protibial spur thin and spiniform. Inner apical angle of protibia with spiniform projection. Metatrochanter without a dorsal hook-shaped protrusion or only with a slight and small protuberance. Medially, ventrite I not expanded posteriorly, broader than ventrite II; ventrites II–V almost with the same width, only V slightly broadest. Ventrites V–VI not strongly narrow medially and VI broader than V. Ventrite I is not elevated in females, even in those species where the male characteristically possesses this elevation. Additionally, in the species where the elevated ventrite I is accompanied by small punctures, these punctures are absent in the female.

**Variation**. All species exhibit allometric variation in the males. This variation is present in the length of the metatibial spur insertion, the size of the tubercles and/or carinae on the internal margin of the metatibia, and the metatibial spur (fused or articulated) in all species. In species with the protibia strongly broadened toward the apex, the degree of this broadened varies. Additionally, in species with the base of the first ventrite elevated, the size of this elevation varies, and in species with a denticle or protuberance on the mesofemur, the size of this structure varies. All these variations are a continuum. Some males exhibit an elongated metatibia, but it is difficult to determine if the spur is still articulated or completely fused. In such cases, they are considered medium-sized males, as exemplified by the holotype of *D. altiventris* sp. nov. (see Fig. 19G).

**Composition**. *Deltochilum plebejum* Balthasar, 1939, *D. abdominale* Martínez, 1947, *D. tyba* sp. nov., *D. pseudoabdominale* sp. nov., *D. parapseudoabdominale* sp. nov., *D. picachos* sp. nov., *D. pauxi* sp. nov., *D. altiventris* sp. nov., *D. ventripuncturatus* sp. nov. and *D. nobile* sp. nov.

**Distribution (Fig. 2)**. Eastern cordillera of the Andes (Colombia), Venezuelan Andes, and The Sierra Nevada de Santa Marta (Colombia).

### Species account

#### Deltochilum abdominale

Taxon classificationAnimaliaColeopteraScarabaeidae

Martínez, 1947

9C909A09-14A2-55C9-BFB9-90B8B8A91CCD

**Figure 4.**
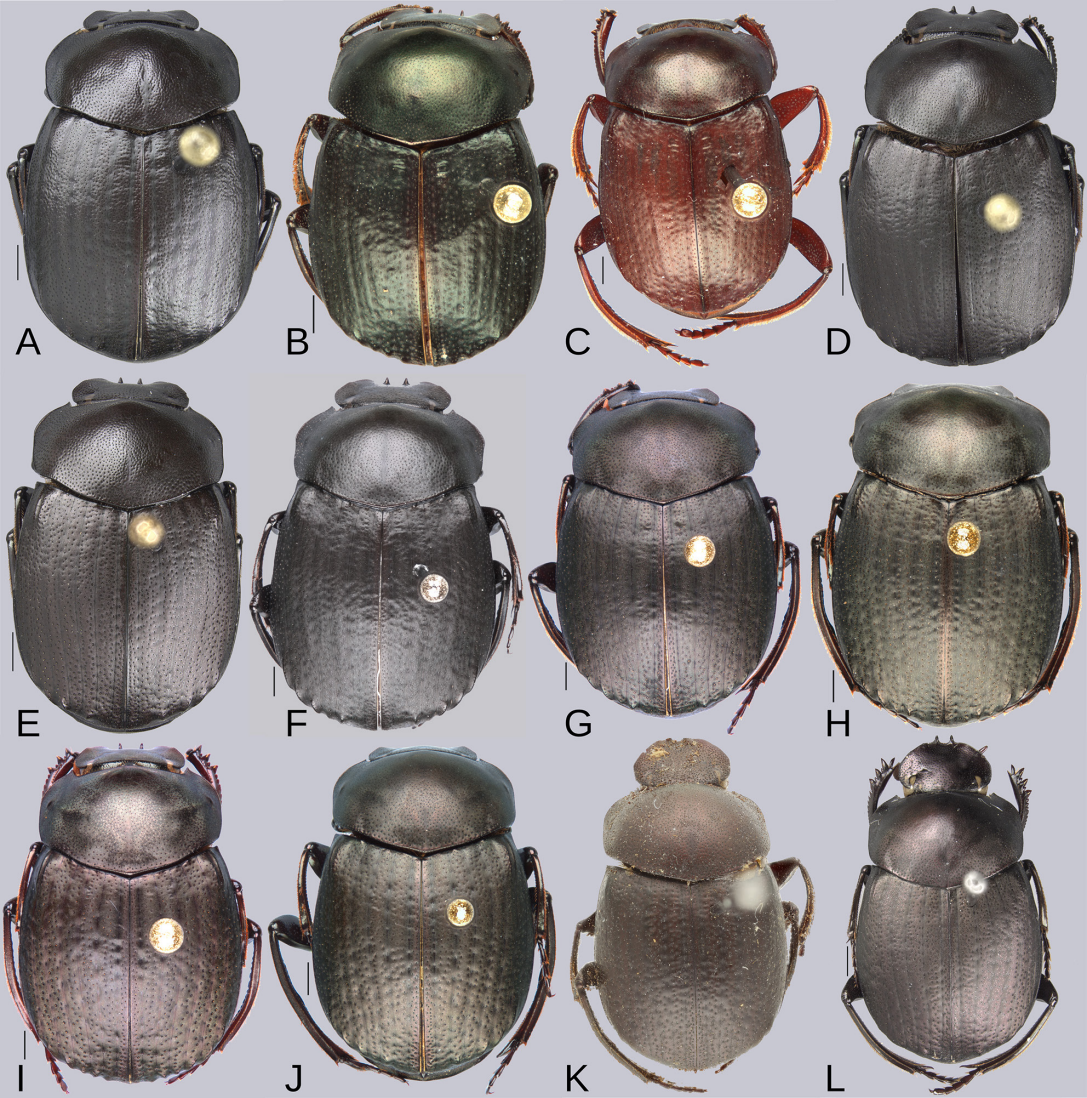
Dorsal habitus of the species of the plebejum species group. **A–K**. Males, **L**. Female. **A**. *Deltochilum abdominale* Martínez, 1947; **B**. Holotype of *D. tyba* sp. nov.; **C**. Paratype of *D. tyba* sp. nov.; **D**. Holotype of *D. pseudoabdominale* sp. nov.; **E**. Holotype of *D. parapseudoabdominale* sp. nov.; **F**. Holotype of *D. picachos* sp. nov.; **G**. Holotype of *D. pauxi* sp. nov.; **H**. Holotype of *D. altiventris* sp. nov.; **I**. Holotype of *D. ventripuncturatus* sp. nov.; **J**. Holotype of *D. nobile* sp. nov.; **K**. Lectotype of *D. plebejum* Balthasar, 1939; **L**. Paralectotype of *D. plebejum*. Scale bars: 1 mm.

**Figure 5.**
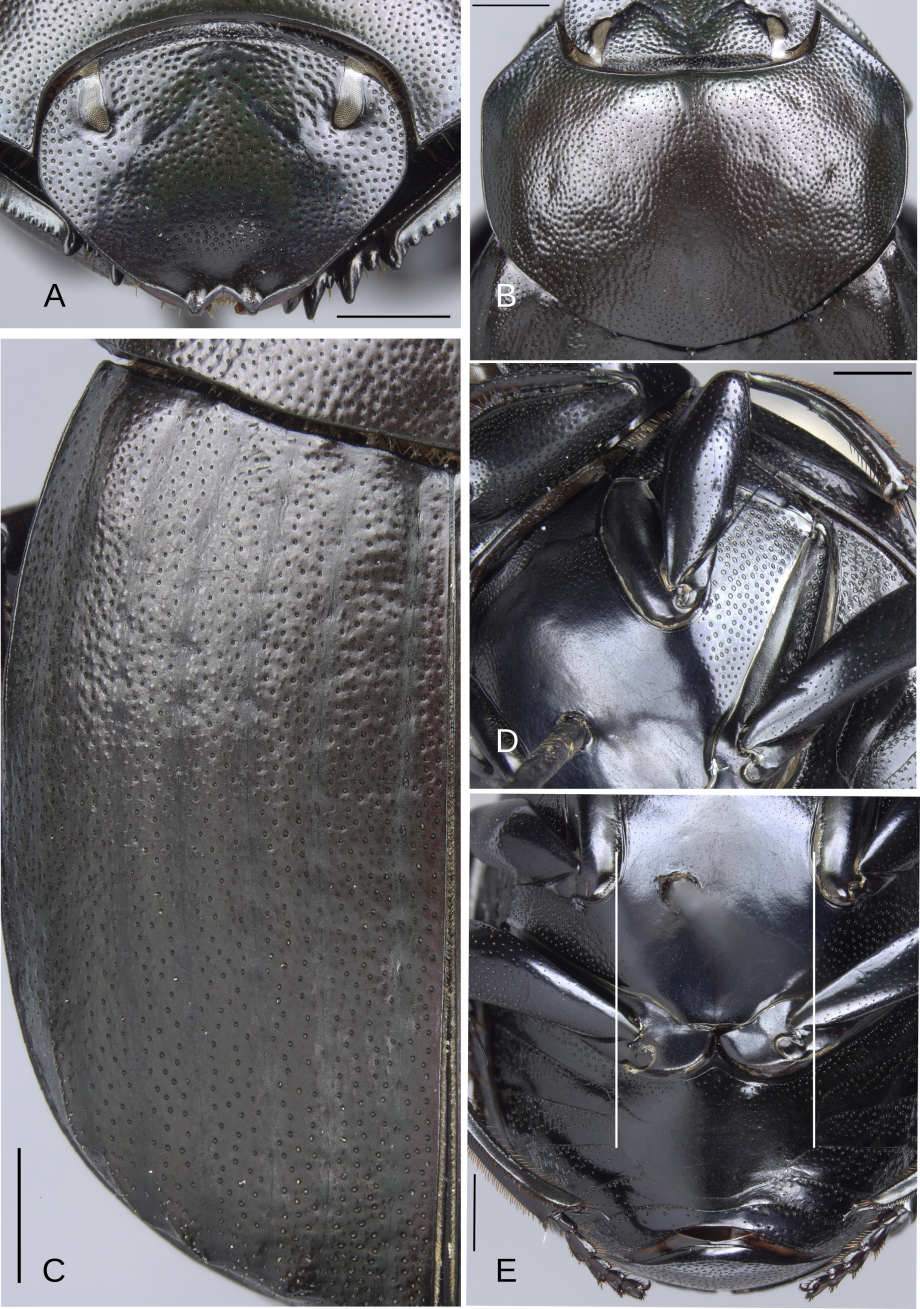
Morphology of *Deltochilum abdominale* Martínez, 1947, male**. A**. Head; **B**. Pronotum; **C**. Elytra; **D**. Metaventrite; **E**. Abdomen, ventrites, white lines showing the comparison of the width of the expansion of the ventrite I (on ventrite III) and the distance between the mesocoxae. Scale bars: 1 mm.

**Figure 6.**
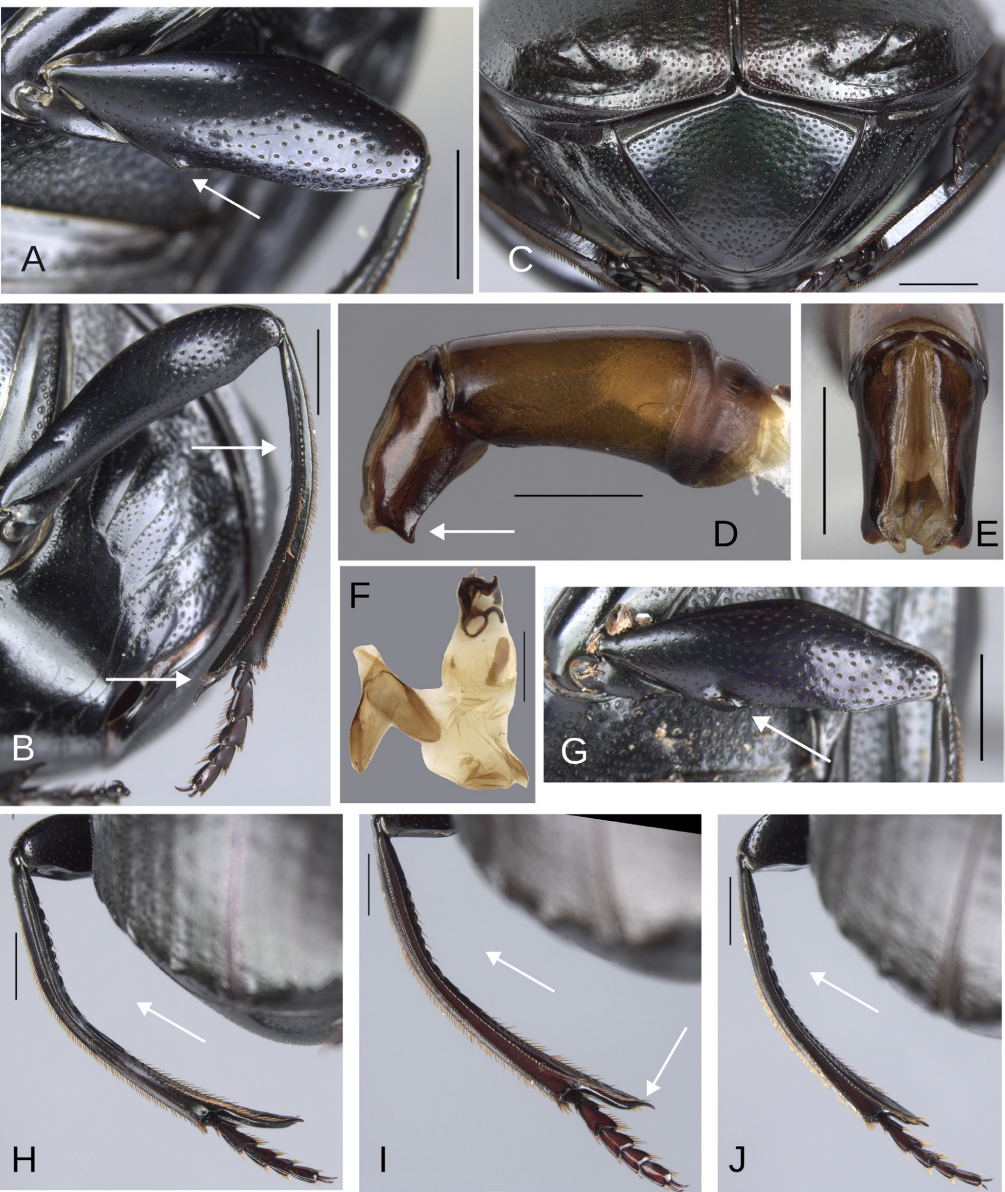
Morphology of *Deltochilum abdominale* Martínez, 1947, **A–F**. Minor male, **G–I**. Major male, **J**. Medium-sized male**. A**. Mesofemur, arrow showing the denticle; **B**. Metatibia, arrows showing the internal carina and the metaspur articulated; **C**. Pygidium; **D**. Aedeagus, lateral view, arrow showing the large denticle; **E**. Parameres, dorsal view; **F**. Endophallus; **G**. Mesofemur, arrow showing the denticle; **H**. Metatibia, arrow showing the internal carina; **I**. Metatibia, arrows showing the internal carina and the metaspur articulated; **J**. Metatibia, arrow showing the internal carina. Scale bars: 1 mm.

**Figure 7.**
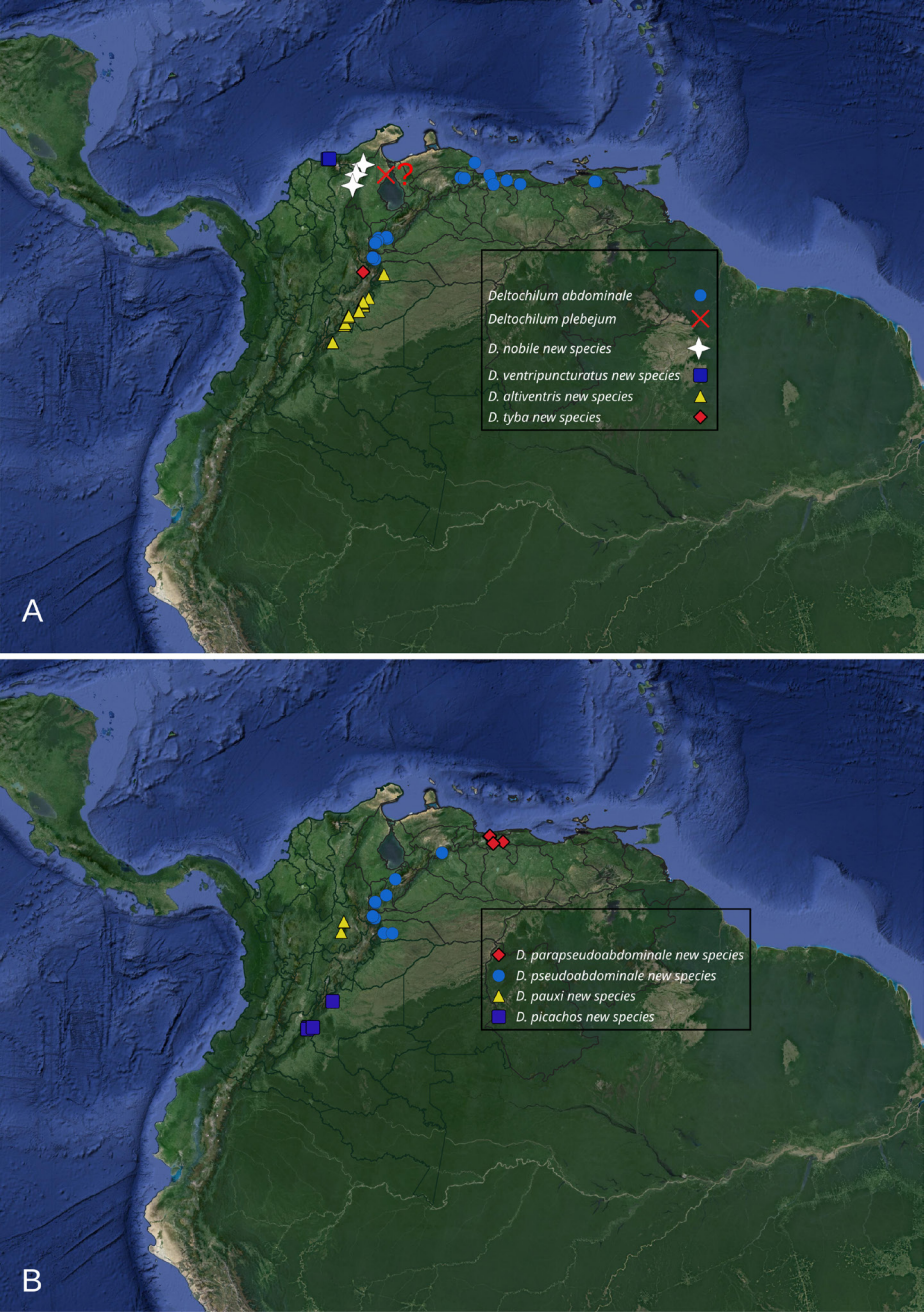
Known distribution of the species of the plebejum species group. **A**. *Deltochilum abdominale* Martínez, 1947, *D. plebejum* Balthasar, 1939, *D. nobile* sp. nov., *D. ventripuncturatus* sp. nov., *D. altiventris* sp. nov. and *D. tyba* sp. nov.; **B**. *Deltochilum parapseudoabdominale* sp. nov., *D. pseudoabdominale* sp. nov., *D. pauxi* sp. nov., *D. picachos* sp. nov. Question mark “?” indicates the unspecified locality (Maracaibo Basin) of *D. plebejum* Balthasar, 1939.

Figs 3B, 3G, 4A, 5, 6, 7A (blue circle)

Deltochilum (D.) abdominalis Martínez, 1947: 269–274. Lámina VIII (description).
Deltochilum (Deltohyboma) abdominalis Martínez 1954: 47 (cited from Colombia, error; see D. ventripuncturatus sp. nov.).
Deltochilum (Deltohyboma) abdominale Vulcano & Pereira, 1964: 652 (catalogue).
Deltochilum (Deltohyboma) abdominalis Blanco 1987: 43 (distribution, Venezuela).
Deltochilum abdominale Medina et al. 2001: 136 (cited from Guaviare (gv?) Colombia with doubts).
Deltochilum (Deltohyboma) abdominalis González-Alvarado & Vaz-de-Mello, 2021: 62, 74 (species group).
Deltochilum abdominale Lozano De La Rosa et al. 2024: 72 (declination of the species epithet in neuter, distribution, Venezuela).

##### Type material examined.

***Holotype*** ♂ Venezuela, D.[istrito] F.[ederal], 720 mts alt., Mo. del Naiguatá, AGO. 941. R. Lichy. Legit. Coll Martínez. [white label] HOLOTIPO. [red label] Deltochilumabdominalis [male symbol] sp. nov. A. Martínez-DET. 946. [white label] FICHADO. [white label] MACN-En 790. (MACN).

##### Additional material examined

**(81 specimens)**: **Colombia: Norte de Santander**: Toledo, IP Santa María, Alto de Herrera, Vda. Diamante, Fca. La Primavera. PNN Tamá, 7°7'N, 72°13'W, 1000 m, 1♀, 2♂, 1999.ix.22, González E., T. Exc. Hum (IAvH), Toledo, Vda. San Alberto. Bosque, 07°11'08.8"N, 072°18'39.6"W, 823 m, 2♂, 11.iv.2019, Ramírez, M. & Lopera, A., T.Exc.H #SAF1 (24 h) (1♂ IAvH, 1♂, CALT-ECC), Toledo, Vda. San Alberto. Bosque, 07°11'15.0"N, 072°18'28.3"W, 1104 m, 1♀, 11.iv.2019, Ramírez, M. & Lopera, A., T.Exc.H #SAF3 (24 h) (IAvH). **Venezuela**: Chichiriviche (Litoral, D.F), [10°56'1.51"N], [68°16'40.14"W], 1♀, 27.i.1977, Bordón coll. (BDGC); **Aragua**: Cuyagua, [10°26'42"N], [67°41'44"W], 800 m, 2♀, 1♂, x.1972, Martínez coll. (CMNC), Quebrada Coirral da Piedra, El Limón, [10°19'N], [67°39'W], 700 m, 1♂, 7.vi.1973, Bordón coll. (CMNC), Racho Grande, [10°4'1.40"N], [67°32'34.02"W], 1200 m, 1♂, 27–30.viii.1983, B Gill coll. (BDGC), Racho Grande, [10°4'1.40"N], [67°32'34.02"W], 1150 m, 1♂, 4–9.vii.1986, B Gill coll. (BDGC); **Miranda**: Parque Nacional Guatopo, [10°5'37"N], [66°29'41"W], 6♀, 6♂, 3–8.iii.1988, F. Génier coll., pitfall trap, human feces (CMNC), Parque Nacional Guatopo, 50 km SE Caracas. forest, [10°5'N], [66°29'W], 400 m, 2♀, 1♂, 5–6.iii.1971, S. Peck coll., pitfall trap, human dung (CMNC), Santa Crucita, 33 km N Altagracia, Parque Nacional Guatopo. forest, [10°14'N], [67°1'W], 400 m, 1♂, 7–10.vi.1987, S. & J. Peck coll., dung trap (CMNC); **Monagas**: Cueva Guácharo, Caripe. forest over coffee, [10°11'N], [63°32'30"W], 750 m, 1♀, 1♂, 20–31.vii.1987, S. & J. Peck coll. (CMNC), Teresén, [10°11'N], [63°29'W], 800 m, 3♂, 12.iv.1963, R. Hernandez coll. (CMNC); **Táchira**: 20 km NE San Cristobal, [7°57'34"N], [72°5'3"W], 3000 m, 1♂, 20.v.1974, S. Peck coll. (CMNC), 20 km NE San Cristobal, [7°57'34"N], [72°5'3"W], 1200 m, 1♂, 20–22.v.1974, S. Peck coll. (CMNC), La Trampita, Presa La Honda, [7°56'1"N], [71°43'24"W], 1100 m, 1♀, 17.xii.1988, D. Havranek coll., mini-dung cup traps (CMNC), Las Trampitas, Pregonero. sub-mountain forest, [8°0'40"N], [71°45'38"W], 1240 m, 10♀, 11♂, 9–14.vii.1989, S. & J. Peck coll., carrion traps (CMNC), Pregonero Las Trampitas, [7°59'48.89"N], [71°45'49.08"W], 1240 m, 2♀, 4♂, 9–14.vii.1989, J Peck & S Peck coll. (BDGC), Rio Frio, [7°50'37.11"N]?, [72°6'33.48"W]?, 500 m, 3♀, 3♂, 11–18.viii.1983, B Gill coll. (BDGC), San Cristobal, [7°45'30.99"N], [72°12'20.62"W], 1200 m, 3♀, 6♂, 10–18.viii.1983, B Gill coll. (BDGC); **Yaracuy**: Bolívar. Aroa, 10°20'22.74"N, 68°50'5.71"W, 1372 m, 1♀, 1♂, 19.VII.2009, Asmüssen M, Colmenares P., Martínez H., Cebo Heces humanas (CEMT).

##### Diagnosis.

This species can be distinguished from all other species within the plebejum species group by having the smallest interstrial punctures (Fig. 5C). These punctures are nearly the same size as the interstrial shiny points; in all other species of the group, these shiny points are distinctly smaller than the interstrial punctures. Furthermore, the males possess a denticle on the mesofemur (Fig. 6A, G), a character shared with *D. tyba* sp. nov. (Fig. 9G), but the two species can be separated by the size of the interstrial punctures previously described.

##### Redescription.

Minor male. Size 10 mm. ***Color***. Dark brown with some blue reflections dorsally. Dark blue ventrally. ***Head*** (Fig. 5A). Interocular distance 8× the width of one eye. Punctures on frons separated by one or less than one diameter of each puncture and subequal in size to head discal punctures. Disc punctures separated by one or less than one diameter of each puncture. Punctures from disc towards anterior area successively of almost with the same size. Genal punctures subequal in size to the discal punctures and separated by one diameter of each puncture. ***Pronotum*** (Fig. 5B). Margin between anterior and medial-lateral angle almost straight. Medial angle of pronotum rounded. Margin between medial-lateral angle and posterior-lateral angle almost straight. Disc punctures half size to anterior-lateral ones and separated by more than one diameter of each puncture. Basal punctures are smaller than anterior-lateral ones and separated by approximately one diameter of each puncture. ***Hypomera***. With ocellated and setigerous punctures on the anterior part (Fig. 3B, white arrow). Most of punctures incompletely closed posteriorly and without setae (Fig. 3B, green arrow). Anterior punctures denser and larger than posterior ones. ***Elytra*** (Fig. 5C). Carina of the ninth interstria almost reaching middle of elytral length. Elytral apex on interstriae III, V–VII with tubercles. Striae I–VII almost inconspicuous. Width of third stria on disc ~1/55 of the distance between striae II and III. Strial punctures ~2× wider than the stria. First stria as wider as second stria. Stria VIII conspicuous apically, laterally almost inconspicuous. Punctures on interstriae approximately separated by two diameters of each puncture and the same size or slightly smaller than strial punctures. Punctures of the third interstria on disc occupying ~ 1/18 of the distance between striae II and III. Interstriae with shiny points mixed with the punctures. Points almost the same size as the interstrial punctures. ***Metaventrite*** (Fig. 5D). Disc with few deep excavation, occupying metaventral basal third. Anterolateral areas of metaventral process with punctures completely closed and separated by less than one diameter of each puncture. Anterocentral area of metaventral process with few and small punctures, subequal in size to the discal punctures. Punctures on the lateral lobules not completely closed (some of them) and separated by one or less than one diameter of each puncture. ***Abdomen*** (Fig. 5E). Ventrite I expanded posteriorly, expansion reaching the fifth ventrite; on ventrite III, the expansion is as wide as the distance between mesocoxae. Basal part of the ventrite I, between the metacoxae not modified. ***Legs*** (Fig. 6A, B). Protibia strongly broadens toward the apex. Profemora with punctures separated by less than one diameter of each puncture. Mesofemur with punctures separated by 2× the diameter of each puncture. Mesofemur with a basal denticle on the posterior edge. Metafemur regularly broadens toward the apex. Metafemur with punctures separated by 2× the diameter of each puncture. Metatibial spur insertion not elongated. Metatibial spur clearly distinct from the metatibia. Internal margin of metatibia with tubercles on the basal half of the metatibial length. ***Pygidium*** (Fig. 6C). Pygidial punctures almost uniform in size and density. Punctures separated by one or less than the diameter of each puncture. Discal punctures, on the center of the pygidium, small, each puncture occupying ~1/45 of the distance between the lateral pygidial margins. ***Genitalia*** (Fig. 6D–F). Paramera smaller than the phallobase. Apex of paramera with a large denticle in lateral view. Medial area of endophallus with two endophallites, the right ~4× longer than the other.

##### Variation.

The female exhibits the differences described here under the sexual dimorphism section of the species group redescription. Medium-sized males have both the basal denticle of the metafemur and the metatibial tubercles (Fig. 6J, white arrow) that are larger than those found in the minor male. The metaspur is articulated. Major males: the metatibial basal carina is irregular and strongly interrupted by setae, giving it the appearance of tubercles (Fig. 6, white arrow). This carina is significantly reduced in the mid-length of the metatibial; distal to this reduction, the carina rises again (giving the appearance of having both tubercles and carina). Other major males possess this tuberculate basal part, but the reduction is less pronounced, and the tubercles are continuous (Fig. 6I, white arrow). The basal denticle of the metafemur is larger and more strongly curved (Fig. 6G). The metatibia is also more strongly curved (Fig. 6H, I). Metaspur fused.

##### Known distribution

**(Fig. 7A blue circle)**. Colombia, Norte de Santander. Venezuela, Aragua, Miranda, Monagas, Táchira, Yaracuy.

##### Remarks.

This species has been collected in forest, submontane forest and few specimens in forest mixed with coffee. This species is one of the members of the plebejum species group that is widely distributed, possessing a broad altitudinal range from 400 to probably 1,400 m above sea level.

#### Deltochilum tyba sp. nov.

Taxon classificationAnimaliaColeopteraScarabaeidae

6526FD61-3159-5BA9-9C05-4F407091639B

https://zoobank.org/C2ADF872-4361-48D8-9077-07A7D4F8B7CB

Figs 4B, 4C, 7A (red diamonds), 8, 9

##### Type material.

***Holotype***: ♂, **Colombia**: Santander: Capitanejo. Bosque Ribera, 6°36'35.3"N, 72°41'17.9"W, 1256 m, 9/3/15, N.J. Beltran, Pitfall Exc Hum (Ex-CALT-ECC) [IAvH-E-268920]. ***Paratype***: ♂, **Colombia**: Santander: Capitanejo. Bosque Ribera, 6°36'35.3"N, 72°41'17.9"W, 1256 m, 9/3/15, N.J. Beltran, Pitfall Exc Hum (CALT-ECC).

##### Diagnosis.

**Figure 8.**
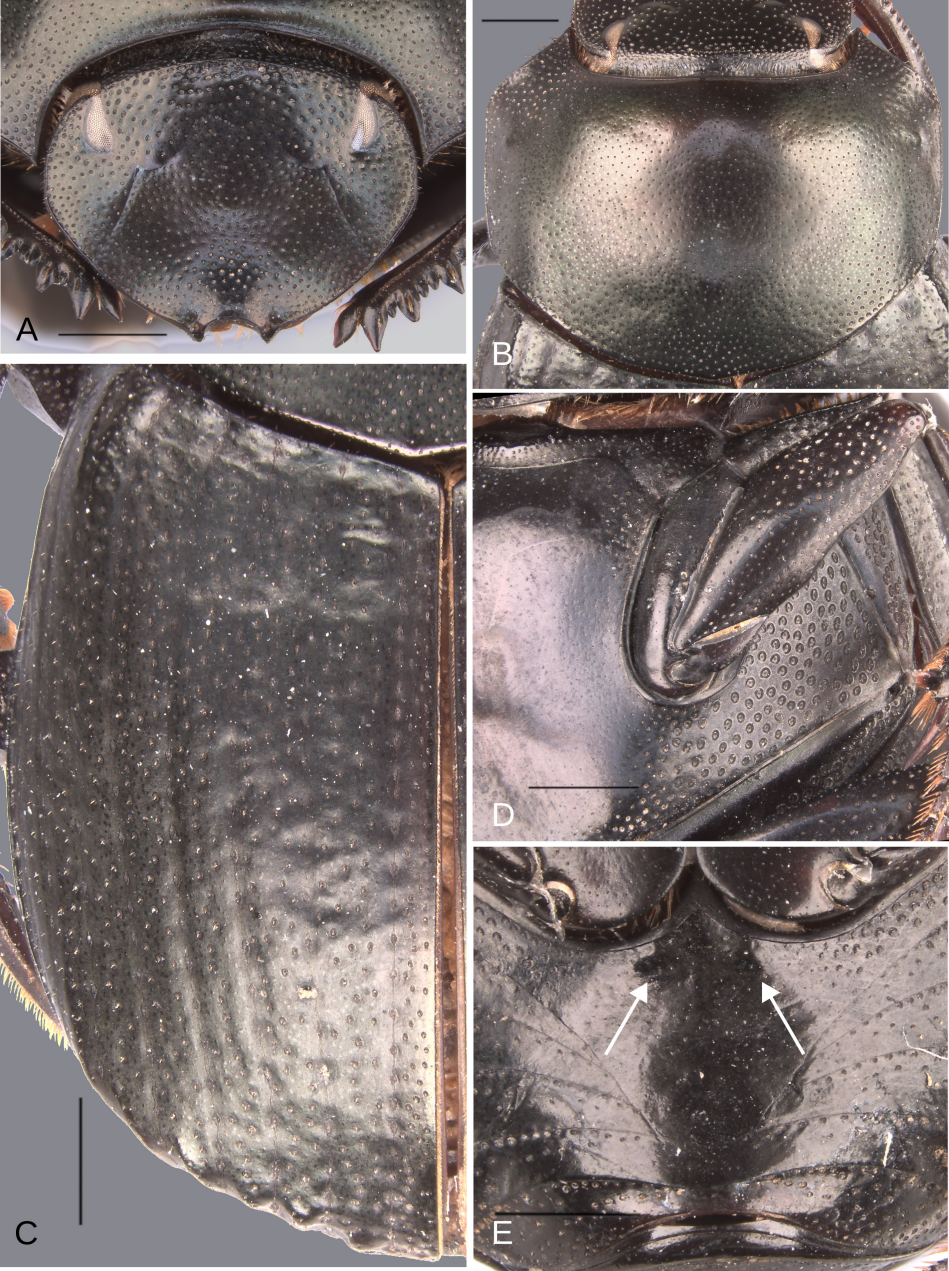
Morphology of *Deltochilum tyba* sp. nov., holotype male. **A**. Head; **B**. Pronotum; **C**. Elytra; **D**. Metaventrite; **E**. Abdomen, ventrites, arrows showing the elevation of the first ventrite. Scale bars: 1 mm.

**Figure 9.**
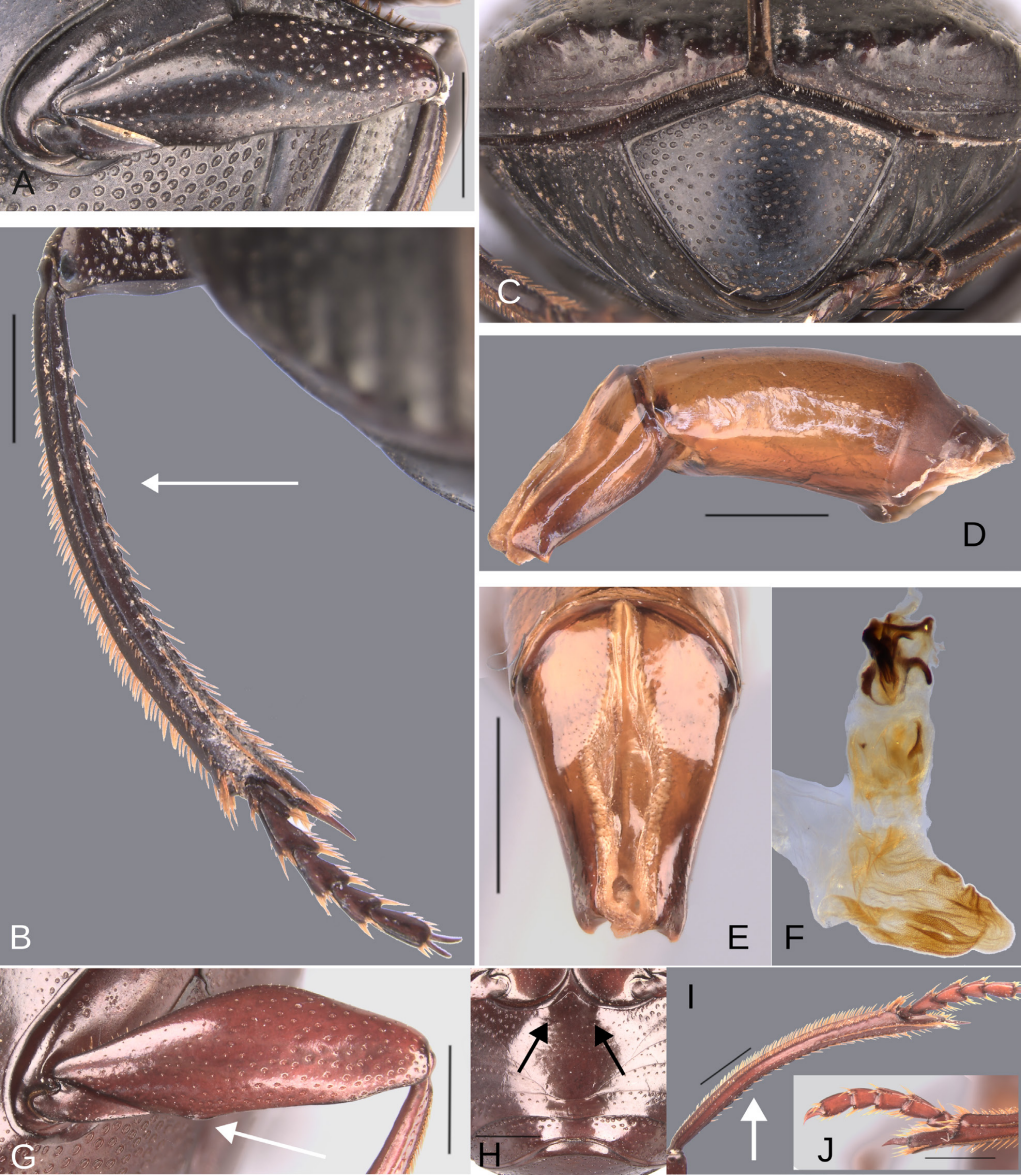
Morphology of *Deltochilum tyba* sp. nov., **A–F**. Holotype male, **G–J**. Paratype, medium-sized male. **A**. Mesofemur; **B**. Metatibia, arrow showing the internal carina; **C**. Pygidium; **D**. Aedeagus, lateral view; **E**. Parameres, dorsal view; **F**. Endophallus; **G**. Mesofemur, arrow showing the denticle; **H**. Abdomen, ventrites, arrows showing the elevation of the first ventrite; **I**. Metatibia arrow showing the internal carina; **J**. Apex of the metatibia. Scale bars: 1 mm.

This species (Fig. 9G) and *D. abdominale* Martínez, 1947 (Fig. 6A, G), are the only two species in which males possess a denticle on the mesofemur. They can be distinguished by the interstrial punctures: these punctures are larger than the interstrial shiny points in *D. tyba* sp. nov. (Fig. 8C), and are nearly the same size as the interstrial shiny points in *D. abdominale* (Fig. 5C).

##### Description.

Minor male. Size 10 mm. ***Color***. Pale green dorsally. Brown ventrally. ***Head*** (Fig. 8A). Interocular distance 10× the width of one eye. Punctures on frons separated by one or more than one diameter of each puncture and subequal in size than discal punctures. Disc punctures separated by one or more than one diameter of each puncture. Punctures from disc towards anterior area successively slightly enlarged. Genal punctures subequal in size and density to the discal punctures. ***Pronotum*** (Fig. 8B). Margin between anterior and medial-lateral angle almost straight. Medial angle of pronotum rounded. Margin between medial-lateral angle and posterior-lateral angle almost straight. Disc punctures half size to anterior-lateral ones and separated by more than one diameter of each puncture. Basal punctures smaller than anterior-lateral ones and separated by approximately one diameter of each puncture. ***Hypomera***. With ocellated and setigerous punctures on the anterior part (as Fig. 3B, white arrow). Most of punctures incompletely closed posteriorly and with setae. Anterior punctures denser and larger than posterior ones. ***Elytra*** (Fig. 8C). Carina of the ninth interstria not reaching middle of elytral length. Elytral apex on interstriae II–VII with tubercles. Striae I–VII almost inconspicuous. Width of third stria on disc ~1/32 of the distance between striae II and III. Strial punctures variable in size, ≥ 2× wider than the stria. First stria slightly wider and deeper than second stria. Stria VIII conspicuous apically and laterally, and reaching carina of the ninth interstria. Punctures on interstriae approximately separated by two diameters. Punctures of the third interstria on disc occupying ~1/10 of the distance between striae II and III. Interstriae with shiny points mixed with the punctures. Points smaller than interstrial punctures, ~3× smaller. ***Metaventrite*** (Fig. 8D). Disc shallowly excavated, occupying the metaventral basal third. Anterolateral areas of metaventral process with punctures completely closed and separated by one diameter of each puncture. Anterocentral area of metaventral process with few and small punctures, slightly larger in size than the discal punctures. Punctures on the lateral lobules not completely closed (some of them) and variable in density, dispersed towards the metaventral disc. ***Legs*** (Fig. 9A, B). Protibia regularly broadens toward the apex. Profemora with punctures separated by less than one diameter of each puncture. Mesofemur with punctures separated by ≥ 2× the diameter of each puncture. Mesofemur with a basal denticle on the posterior edge. Metafemur regularly broadens toward the apex. Metafemur with punctures separated by ≥ 2× the diameter of each puncture. Metatibial spur insertion elongated, with the length subequal to the basal two tarsomeres. Metatibial spur clearly distinct from the metatibia. Internal margin of metatibia with tubercles on the basal half of the metatibial length. ***Abdomen*** (Fig. 8E). Ventrite I expanded posteriorly, expansion reaching the fourth ventrite; on ventrite III, the expansion narrower than the distance between mesocoxae. Basal part of the ventrite I, between the metacoxae elevated. ***Pygidium*** (Fig. 9C). Pygidial punctures almost uniform in size and density. Punctures separated by one or more than the diameter of each puncture. Discal punctures on the center of the pygidium, medium, each puncture occupying ~1/36 of the distance between the lateral pygidial margins. ***Genitalia*** (Fig. 9D–F). Paramera smaller than the phallobase. Apex of paramera with a denticle in lateral view. Medial area of endophallus with two endophallites, the right ~7× longer than the other.

##### Variation.

The medium-sized paratype (Figs 4C, 9J) is a teneral and exhibits the following characteristics: the denticle of the mesofemur larger and more curved (Fig. 9G, white arrow). The metatibial tubercles are larger (Fig. 9I, white arrow). The first ventrite is more elevated (Fig. 9H). Metaspur articulate. The major male and female are unknown.

##### Etymology.

The name tyba is a noun in apposition, derived from the Muisca (Chibcha) indigenous word ‘tyba’ or ‘tybá’, meaning ‘Great Captain’ or ‘Cacique’ (chief). This name is given to reference the type locality, Capitanejo is the diminutive of Capitán, used in allusion to a local indigenous leader.

##### Known distribution

**(Fig. 7A red diamonds)**. This species is only known from the type locality: Colombia: Santander: Capitanejo. Bosque Ribera, 6°36'35.3"N, 72°41'17.9"W, 1256 m.

#### Deltochilum pseudoabdominale sp. nov.

Taxon classificationAnimaliaColeopteraScarabaeidae

6BB1BD39-F308-5A8B-8EFD-5947EA6F1268

https://zoobank.org/57D373A7-0B49-4561-8C82-8B77EEA4DDAE

Figs 1B, 4D, 7B (blue circles), 10, 11

##### Type material.

***Holotype***: ♂, **VEN.[EZUELA]: Mérida**: ULA Biological Reserve, La Carbonerra [Carbonera?], 20 km SE Azulita. *Podocarpus* forest, [8°39'23"N], [71°24'33"W], 2300 m, 28.vi-3.viii.[19]89, S. & J. Peck, malaise (CMNC) [WSD00039446]. ***Paratypes* (48 specimens)**: **Colombia: Arauca**: Tame. Brisas el Cravo. Bosque, 06°31'25"N, 71°51'57"W, 2000 m, 1♀, 14–19/II/08, F. Alvarado, E.H. (CALT-ECC), Tame. Brisas el Cravo. Bosque, 06°31'01"N, 71°31'46"W, 1000 m, 1♀, 20–25/XI/07, F. Alvarado, E.H. (CALT-ECC); **Norte de Santander**: Toledo, IP Santa María, Alto de Herrera, Vda. Diamante, Fca. La Primavera. PNN Tamá, 7°7'N, 72°13'W, 1450 m, 4♀, 5♂, 1999.ix, González E., T. Exc. H. (IAvH), Toledo, IP Santa María, Alto de Herrera, Vda. Diamante, Fca. La Primavera. PNN Tamá, 7°6'N, 72°13'W, 1000 m, 1♀, 1999.ix.22, González E., T. Exc. H. (IAvH), Toledo, IP Santa María, Alto de Herrera, Vda. Diamante, Fca. La Primavera. PNN Tamá, 7°6'N, 72°13'W, 1250 m, 1♂, 1999.ix.30, González E., T. Exc. H. (IAvH), Toledo, IP Santa María, Alto la Herrera, Vda. Diamante, Fca. La Primavera. PNN Tamá. Bosque secundario conservado, 7°7'N, 72°13'W, 1450 m, 2♂, 1999.ix.29, González E., T. Exc. H. 10 (IAvH), Toledo, Vda. San Alberto. Bosque, 07°11'15.0"N, 072°18'28.3"W, 1104 m, 1♂, 14.iv.2019, Ramírez, M. & Lopera, A., T.Exc.H #SAF3 (96 h) (IAvH), Toledo, Vda. San Alberto. Bosque, 07°11'33.1"N, 072°18'13.3"W, 1358 m, 1♂, 14.iv.2019, Ramírez, M. & Lopera, A., T.Exc.H #SAF5 (96 h) (IAvH), Toledo. PNN Tamá, Santa María Alto de Herrera, Vda. El Diamante, Finca La Primavera, 7°7'N, 72°13'W, 1000 m, 2♀, 1♂, 1999.x.25, González, E.Colecta Manual (UPTC-CE), Toledo. PNN Tamá, Santa María Alto de Herrera, Vda. El Diamante, Finca La Primavera. Bosque, 7°7'N, 72°13'W, 1000 m, 2♀, 1999.x.09, González, E.Trampa de excremento humano (UPTC-CE). **Venezuela: Lara**: Yacambu N.P. 14 km SE Sinaré, 9°42'N, 69°34'W, 1650 m, 2♀, 1♂, v.1998, J. Ashe, R Brooks & R Hanley coll., F.I.T. (BDGC), Yacambu N.P. 17 km SE Sinaré, 9°42'N, 69°34'W, 1510 m, 2♀, 18.v.1998, J. Ashe, R Brooks & R Hanley coll., F.I.T. (BDGC); **Táchira**: La Idea, Campamento Siberia, Pregonero. rainforest, [8°1'N], [71°46'W], 1200 m, 1♂, 10–31.vii.1989, S. & J. Peck coll., flight interception trap (CMNC), San Cristóbal, [7°45'30.99"N], [72°12'20.62"W], 1200 m, 12♀, 7♂, 10–18.viii.1983, B Gill coll. (BDGC), Via Chorro del Indio, San Cristóbal, [7°43'20"N], [72°11'31"W], 1♀, iv.1987, D. Havranek coll. (CMNC).

##### Diagnosis.

**Figure 10.**
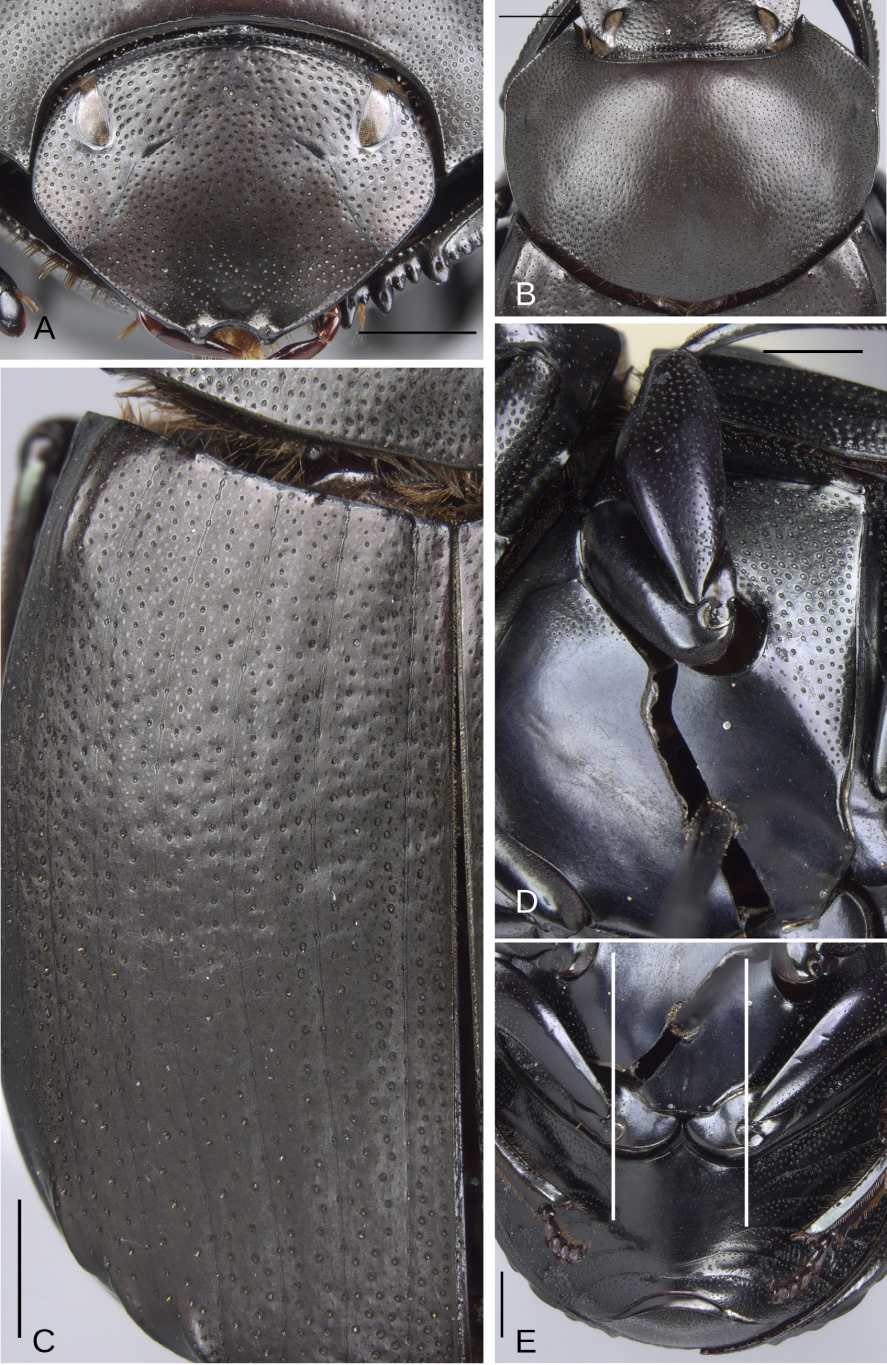
Morphology of *D. pseudoabdominale* sp. nov., holotype male. **A**. Head; **B**. Pronotum; **C**. Elytra; **D**. Metaventrite; **E**. Abdomen, ventrites, white lines showing the comparison of the width of the expansion of the ventrite I (on ventrite III) and the distance between the mesocoxae. Scale bars: 1 mm.

**Figure 11.**
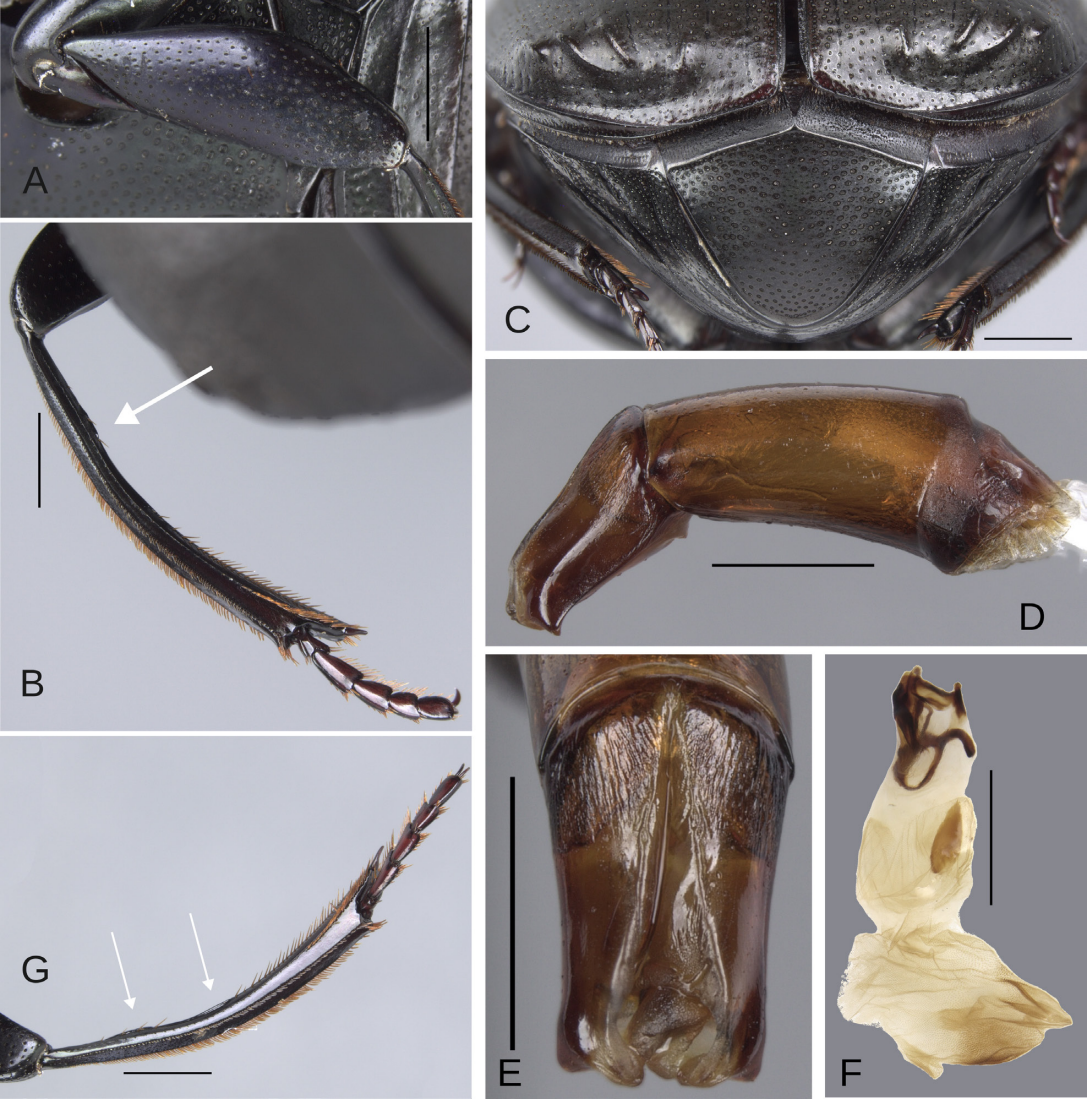
Morphology of *D. pseudoabdominale* sp. nov., **A–F**. Holotype male, **G**. Paratype, medium-sized male **A**. Mesofemur; **B**. Metatibia, arrow showing the internal carina; **C**. Pygidium; **D**. Aedeagus, lateral view; **E**. Parameres, dorsal view; **F**. Endophallus; **G**. Metatibia, arrows showing the internal carina. Scale bars: 1 mm.

**Figure 12.**
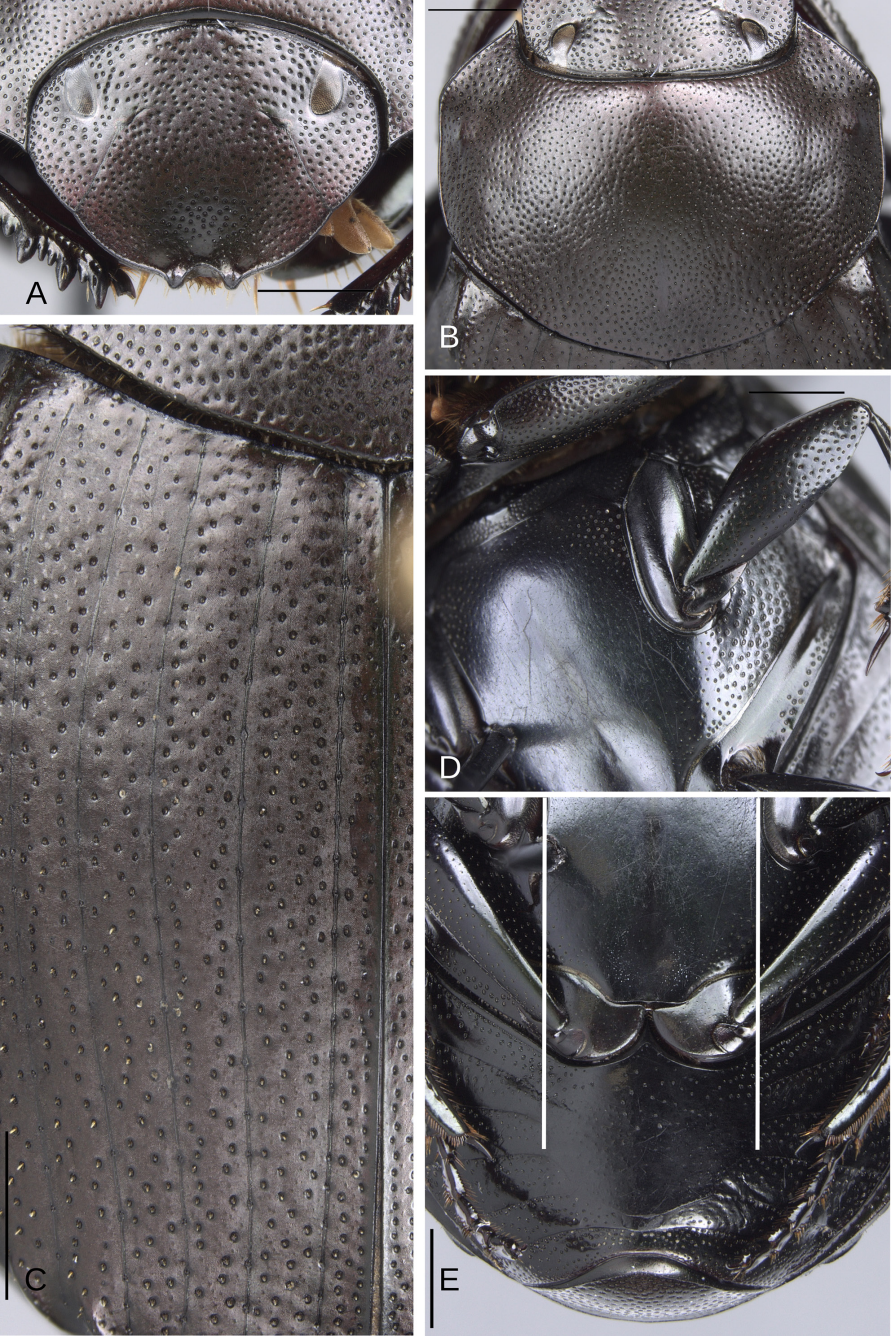
Morphology of *D. parapseudoabdominale* sp. nov., holotype male. **A**. Head; **B**. Pronotum; **C**. Elytra; **D**. Metaventrite; **E**. Abdomen, ventrites, white lines showing the comparison of the width of the expansion of the ventrite I (on ventrite III) and the distance between the mesocoxae. Scale bars: 1 mm.

**Figure 13.**
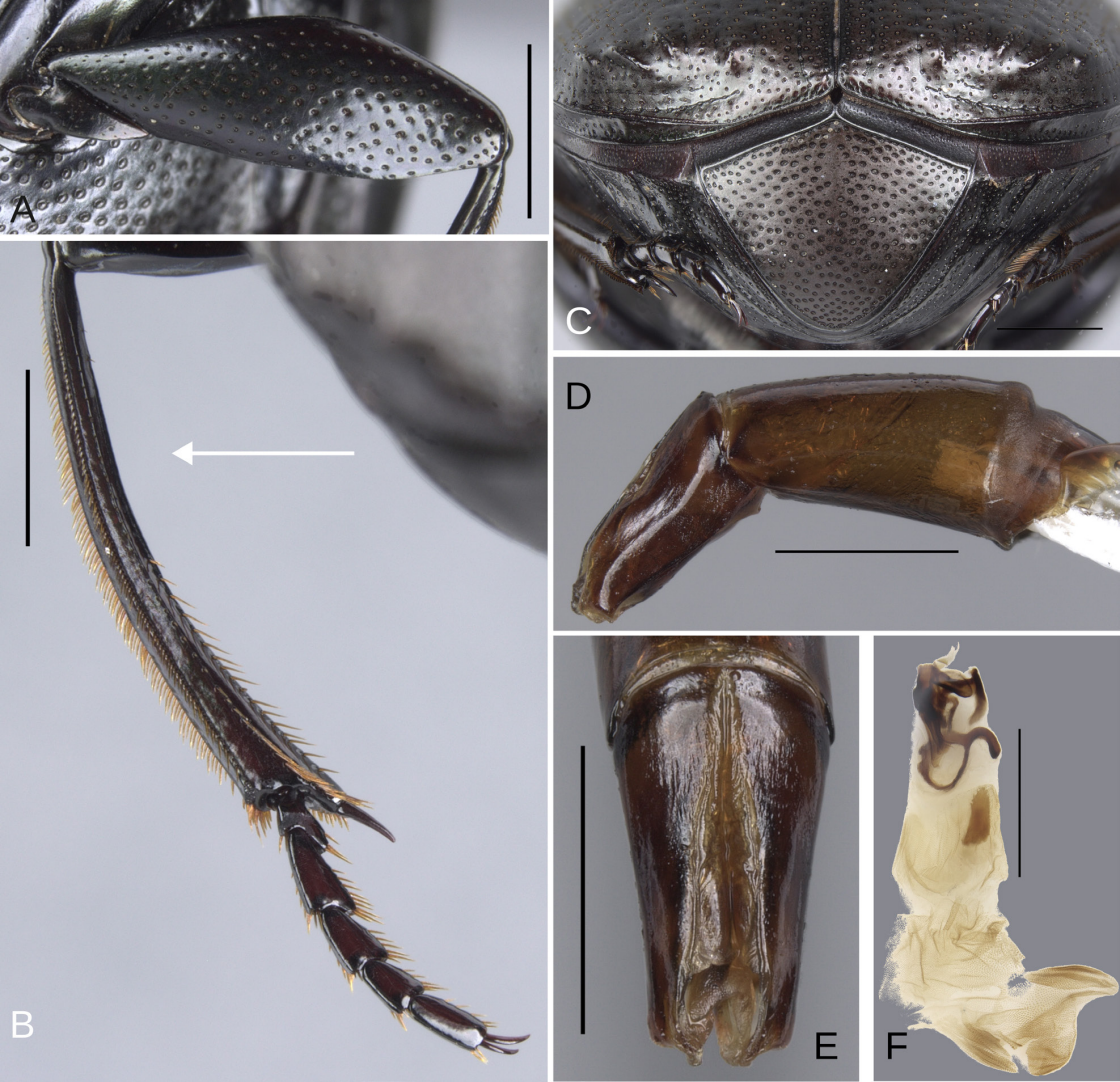
Morphology of *D. parapseudoabdominale* sp. nov., holotype male. **A**. Mesofemur; **B**. Metatibia, arrow showing the internal carina; **C**. Pygidium; **D**. Aedeagus, lateral view; **E**. Parameres, dorsal view; **F**. Endophallus. Scale bars: 1 mm.

**Figure 14.**
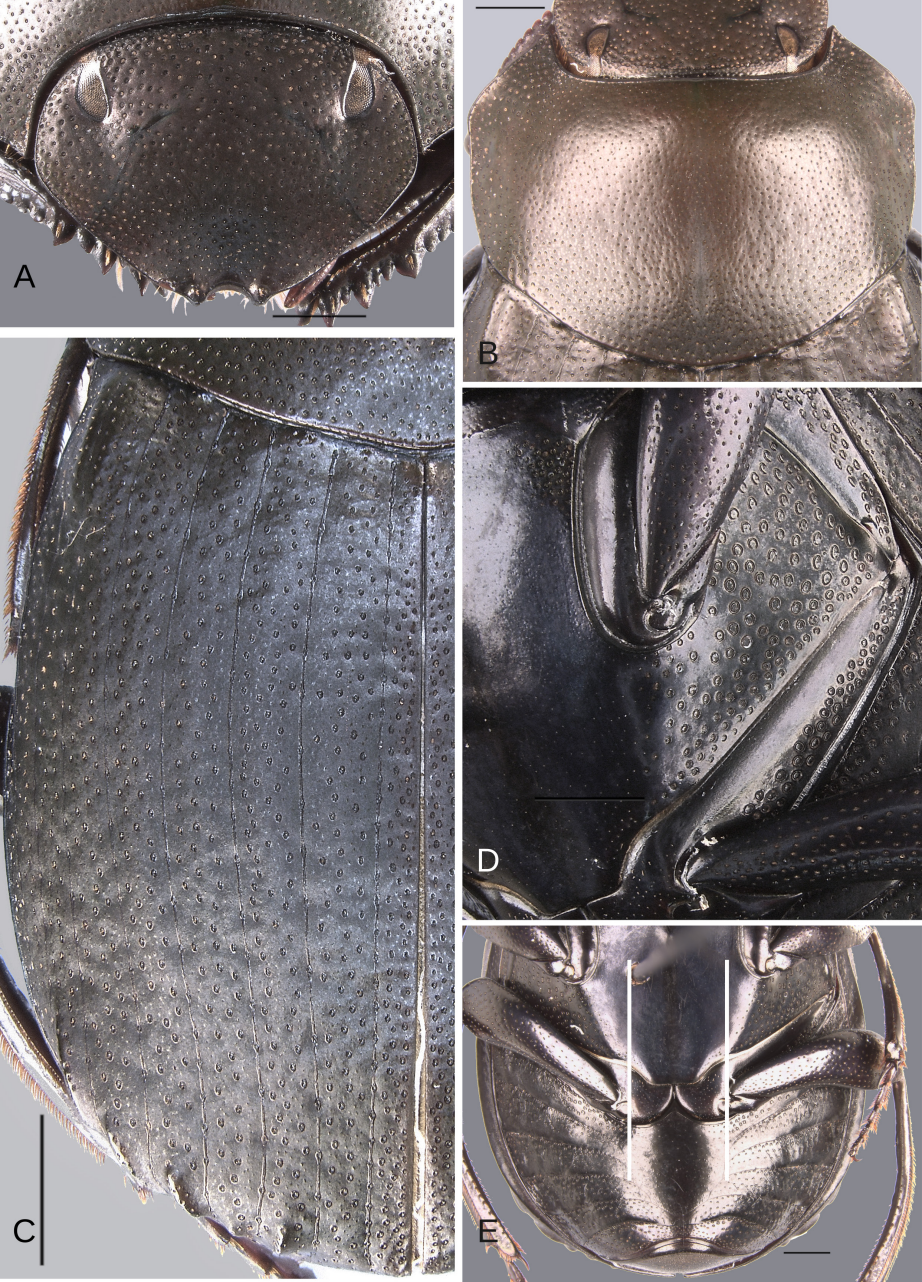
Morphology of *D. picachos* sp. nov., holotype male**. A**. Head; **B**. Pronotum; **C**. Elytra; **D**. Metaventrite; **E**. Abdomen, ventrites, white lines showing the comparison of the width of the expansion of the ventrite I (on ventrite III) and the distance between the mesocoxae. Scale bars: 1 mm.

**Figure 15.**
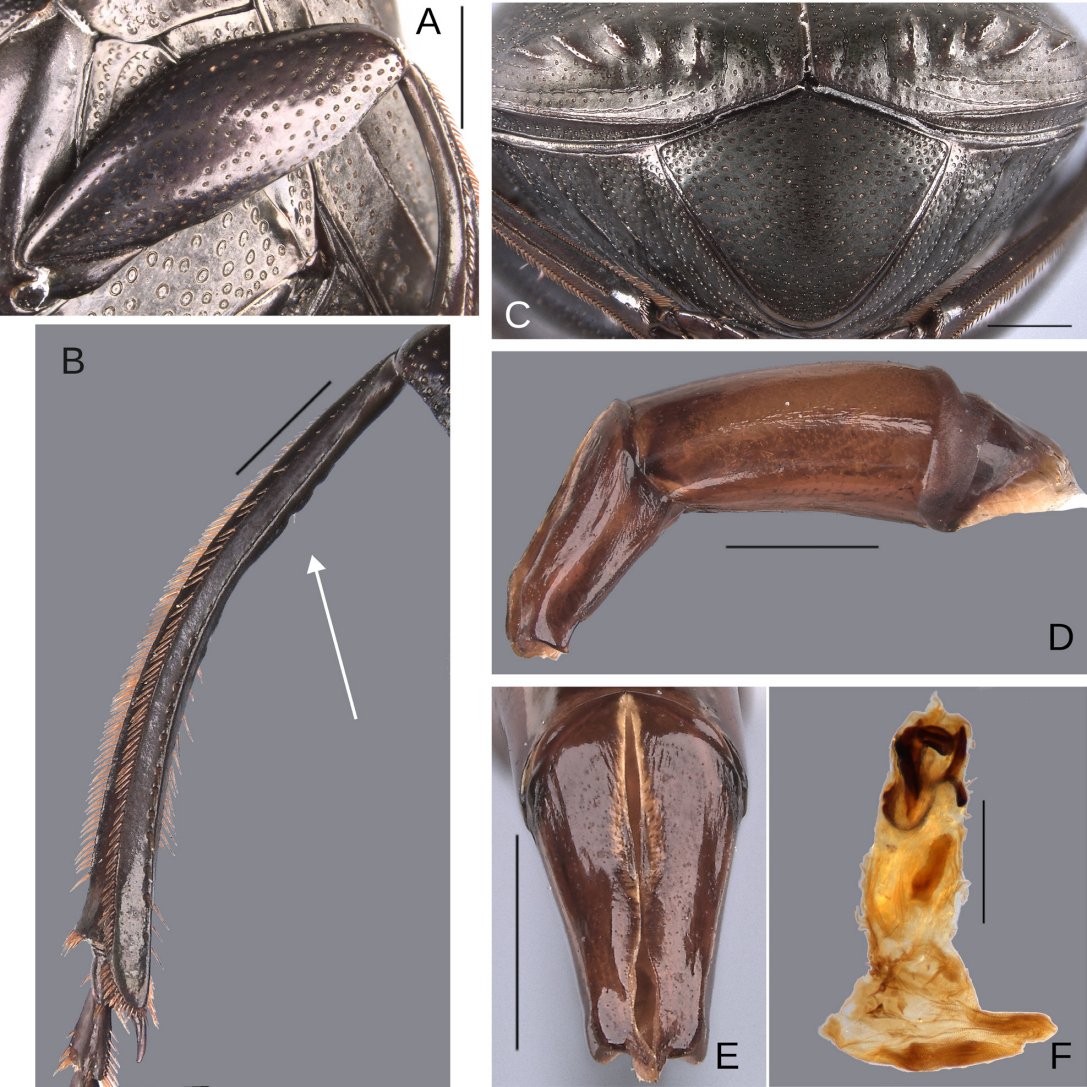
Morphology of *D. picachos* sp. nov., holotype male. **A**. Mesofemur; **B**. Metatibia, arrow showing the internal carina; **C**. Pygidium; **D**. Aedeagus, lateral view; **E**. Parameres, dorsal view; **F**. Endophallus. Scale bars: 1 mm.

**Figure 16.**
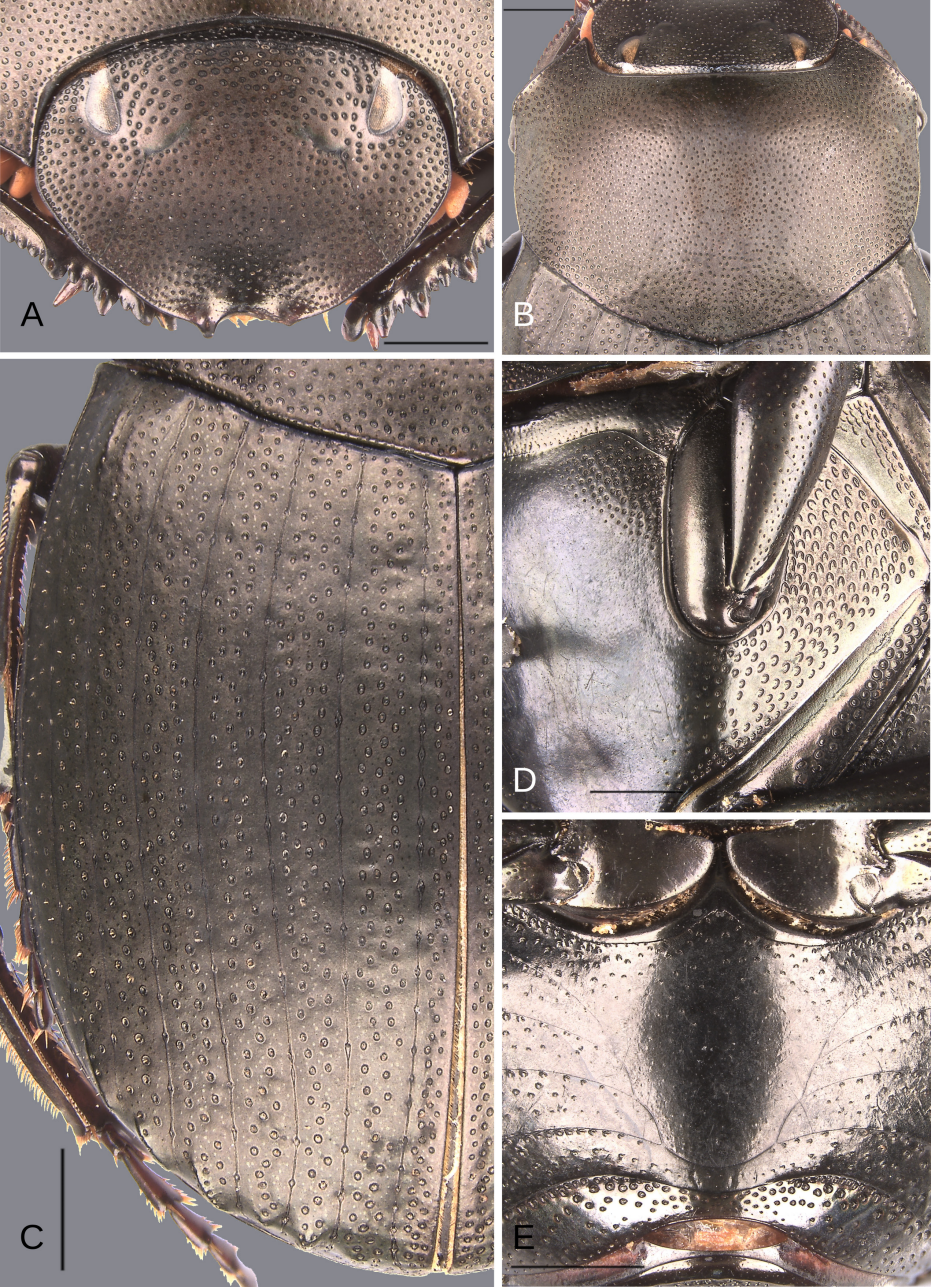
Morphology of *D. pauxi* sp. nov., holotype male. **A**. Head; **B**. Pronotum; **C**. Elytra; **D**. Metaventrite; **E**. Abdomen, ventrites. Scale bars: 1 mm.

**Figure 17.**
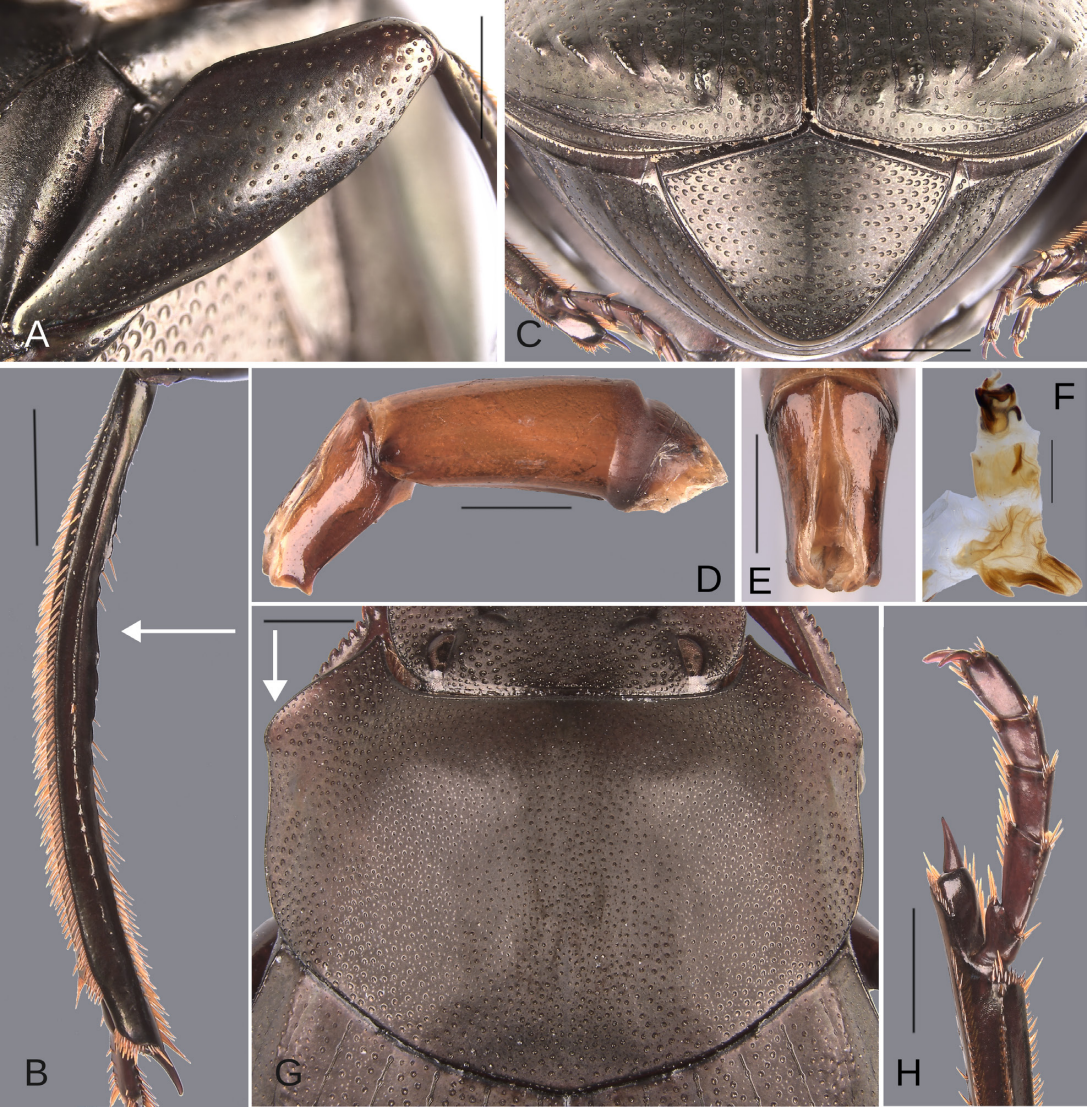
Morphology of *D. pauxi* sp. nov., **A–F**. Holotype male, **G–H**. Paratype, major male. **A**. Mesofemur; **B**. Metatibia, arrow showing the internal carina; **C**. Pygidium; **D**. Aedeagus, lateral view; **E**. Parameres, dorsal view; **F**. Endophallus; **G**. Pronotum, arrow showing the medial angle; **H**. Apex of the metatibia, Scale bars: 1 mm.

This species is very similar to *D. parapseudoabdominale* sp. nov., *D. picachos* sp. nov. and *D. pauxi* sp. nov. by having the striae wide (Figs 10C, 12C, 14C, 16C), and the males with the first ventrite not elevated (Figs 10E, 12E, 14E, 16E) and the mesofemur not modified (Figs 11A, 13A, 15A, 17A). This species can be separated from *D. parapseudoabdominale* sp. nov. by the width of the male first ventrite: at the level of the third ventrite, the width of the first ventrite is narrower than the intermesocoxal distance in this species (Fig. 10E), whereas it is equal to the intermesocoxal distance in *D. parapseudoabdominale* sp. nov. (Fig. 12E). Additionally, they are separated by the punctures on the anterior part of the head: these punctures are slightly larger than the punctures of the head disc in *D. parapseudoabdominale* sp. nov. (Fig. 12A), but are nearly the same size in *D. pseudoabdominale* sp. nov. (Fig. 10A).

From *D. pauxi* sp. nov., this species can be distinguished by the medial angle of the pronotum, which is rounded in *D. pseudoabdominale* sp. nov. (Fig. 10B) and salient in *D. pauxi* sp. nov. (Fig. 17G). Furthermore, this species can be separated from *D. picachos* sp. nov. by the apical tubercles of the elytra: tubercles on interstriae III, V, and VII in *D. pseudoabdominale* sp. nov. (Fig. 11C), whereas are present on interstriae III–VII in *D. picachos* sp. nov. (Fig. 15C).

##### Description.

Minor male. Size 10.30 mm. ***Color***. Dark brown dorsally. Dark blue ventrally. ***Head*** (Fig. 10A). Interocular distance 8× the width of one eye. Punctures on frons separated by one or less than one diameter of each puncture and slightly larger in size than discal punctures. Disc punctures separated by more than one diameter of each puncture. Punctures from disc towards anterior area successively of almost with the same size. Genal punctures subequal in size and density to the discal punctures. ***Pronotum*** (Fig. 10B). Margin between anterior and medial-lateral angle almost straight. Medial angle of pronotum rounded. Margin between medial-lateral angle and posterior-lateral angle subsinuate. Disc punctures half size to anterior-lateral ones and separated by more than one diameter of each puncture. Basal punctures smaller than anterior-lateral ones and separated by approximately two diameters of each puncture. ***Hypomera***. With ocellated and setigerous punctures on the anterior part (as Fig. 3B, white arrow). Most of punctures incompletely closed posteriorly and without setae. Anterior punctures denser and larger than posterior ones. ***Elytra*** (Fig. 10C). Carina of the ninth interstria almost reaching middle of elytral length. Elytral apex on interstriae III, V–VII with tubercles. Striae I–VII conspicuous. Width of third stria on disc ~1/24 of the distance between striae II and III. Strial punctures ~2× wider than the stria. First stria slightly wider and deeper than second stria. Stria VIII almost inconspicuous apically and laterally and reaching carina of the ninth interstria. Punctures on interstriae approximately separated by two diameters and the same size or slightly larger than strial punctures. Punctures of the third interstria on disc occupying ~1/12 of the distance between striae II and III. Interstriae with shiny points mixed with the punctures. Points smaller than interstrial punctures, almost half the size. ***Metaventrite*** (Fig. 10D). Disc shallowly excavated, occupying the metaventral basal third. Anterolateral areas of metaventral process with punctures completely closed and separated by less than one diameter of each puncture. Anterocentral area of metaventral process with few and small punctures, subequal in size to the discal punctures. Punctures on the lateral lobules closed and variable in density, dispersed towards the metaventral disc. ***Legs*** (Fig. 11A, B). Protibia regularly broadens toward the apex. Profemora with punctures separated by less than one diameter of each puncture. Mesofemur with punctures separated by 2× the diameter of each puncture. Mesofemur unarmed on the posterior edge. Metafemur regularly broadens toward the apex. Metafemur with punctures separated by 2× the diameter of each puncture. Internal margin of metatibia with a small carina on the basal third. Metatibial spur insertion not elongated. Metatibial spur clearly distinct from the metatibia. ***Abdomen*** (Fig. 10E). Ventrite I expanded posteriorly, expansion reaching the fifth ventrite; on ventrite III, the expansion narrower than the distance between mesocoxae. Basal part of the ventrite I, between the metacoxae not modified. ***Pygidium*** (Fig. 11C). Pygidial punctures variable in size and density. Punctures separated by one or less than the diameter of each puncture on the disc, more disperse towards the apex. Discal punctures, on the center of the pygidium, medium, each puncture occupying ~1/35 of the distance between the lateral pygidial margins. ***Genitalia*** (Fig. 11D–F). Paramera smaller than the phallobase. Apex of paramera with a denticle in lateral view. Medial area of endophallus with one endophallite.

##### Variation.

The female exhibits the differences described here under the sexual dimorphism section of the redescription of the species group. Medium-sized male: the metatibial basal carina is stronger. This carina is significantly reduced in the middle of the metatibial length; following this reduction, the carina rises again (giving the appearance of having two carinae) (Fig. 11G). Metafemur strongly broadened towards the apex. Metaspur articulate. Major males: the metatibial basal carina stronger. This carina is significantly reduced in the middle of the metatibial length; following this reduction, the carina rises again (giving the appearance of having two carinae). Metafemur strongly broadened toward the apex. Metaspur fused (Fig. 1B).

##### Etymology.

The name, *pseudoabdominale*, is an adjective derived from the Greek prefix *pseudēs*-, meaning false or resembling, and *abdominale*. This name is given in reference to the sympatry of this new species with *Deltochilum abdominale*.

##### Known distribution

**(Fig. 7B blue circles)**. Type locality: Venezuela: Mérida: ULA Biological Reserve, La Carbonerra [Carbonera?], 20 km SE Azulita. *Podocarpus* forest, [8°39'23"N], [71°24'33"W], 2300 m. This species in known from Colombia, Arauca, Norte de Santander and Venezuela, Lara, Mérida y Táchira.

##### Remarks.

This species has been collected in forest, conserved secondary forest, rainforest, and in *Podocarpus* forest. This species is one of the members of the plebejum species group that is widely distributed alongside *D. abdominale*. These two species are found in sympatry. *Deltochilum pseudoabdominale* sp. nov. appears to have a more restricted altitudinal range (from 1,000 to probably 1,400 m) than *D. abdominale*.

#### Deltochilum parapseudoabdominale sp. nov.

Taxon classificationAnimaliaColeopteraScarabaeidae

33C9C9DB-0764-51CE-9983-C0C628F35C97

https://zoobank.org/6C365DCF-659C-4D7E-B643-CEE04C0A93CF

Figs 3F, 4E, 7B (red diamonds), 12, 13

##### Type material.

***Holotype***: ♂, **VEN.[EZUELA]: Edo. Aragua**: Portachuela, Rancho Grande, [10°20'51"N], [67°41'15"W], 1100 m, 21–24.ii.1971, S. Peck, limburg (CMNC). ***Paratypes* (33 specimens): Venezuela: Aragua**: Portachuelo Pass, Parque Nacional Henri Pittier, [10°20'51"N], [67°41'15"W], 1200 m, 1♀, 7–13.vi.1999, Ratcliffe, Jameson, Smith, Villatoro coll., flight interception trap (CMNC), Portachuelo, Rancho Grande, [10°20'51"N], [67°41'15"W], 1100 m, 2♀, 19–21.ii.1971, S. Peck coll., human dung (CMNC), 4♀, 6♂, 21–24.ii.1971, S. Peck coll., Limburg (CMNC), Racho Grande, [10°4'1.40"N], [67°32'34.02"W], 1200 m, 2♀, 3♂, 27–30.viii.1983, B Gill coll. (BDGC), Racho Grande, [10°4'1.40"N], [67°32'34.02"W], 1460 m, 2♀, 3♂, 4–14.vii.1983, B Gill coll. (BDGC), Rancho Grande [site 2], [10°20'59"N], [67°40'55"W], 1100 m, 1♀, 22–23.ii.1971, H. & A. Howden coll. (CMNC), Rancho Grande [site 5], [10°21'34"N], [67°40'32"W], 1500 m, 1♀, 2♂, 21–25.ii.1971, S. Peck coll., carrion trap (CMNC), 3♀, 21–25.ii.1971, S. Peck coll., human dung trap (CMNC), Rancho Grande [site 7], [10°20'N], [67°41'W], 1460 m, 1♀, 1♂, 4–14.vii.1986, B. Gill coll. (CMNC), Tiara, 50 km SW Caracas. forest, [10°7'47"N], [67°9'20"W], 2000 m, 1♂, 22–25.ii.1971, S. Peck coll., human dung (CMNC).

##### Diagnosis.

This species is very similar to *D. pseudoabdominale* sp. nov., *D. picachos* sp. nov., and *D. pauxi* sp. nov. by having the striae wide (Figs 10C, 12C, 14C, 16C), and the males with the first ventrite not elevated (Figs 10E, 12E, 14E, 16E) and the mesofemur not modified (Figs 11A, 13A, 15A, 17A). It can be separated from these species by having the male first ventrite wide, on the third ventrite, the width of the first ventrite is as wide as the intermesocoxal distance (Fig. 12E), in all other species the width of the first ventrite is narrower than the intermesocoxal distance (Figs 10E, 14E, 16E). Additionally, it can be differentiated from *D. pseudoabdominale* sp. nov. and *D. picachos* sp. nov. by the punctures on the anterior part of the head: they are slightly larger than the punctures of the head disc in *D. parapseudoabdominale* sp. nov. (Fig. 12A), but are nearly the same size in the other two species (*D. pseudoabdominale* sp. nov. and *D. picachos* sp. nov.) (Figs 10A, 14A). Furthermore, from *D. pauxi* sp. nov., this species can be distinguished by the medial angle of the pronotum, which is rounded in *D. parapseudoabdominale* sp. nov. (Fig. 12B) and salient in *D. pauxi* sp. nov. (Fig. 17G)

##### Description.

Minor male. Size 9.70 mm. ***Color***. Dark brown with some blue reflections dorsally. Dark blue ventrally. ***Head*** (Fig. 12A). Interocular distance 8× the width of one eye. Punctures on frons separated by one or less than one diameter of each puncture and slightly larger than the head discal punctures. Disc punctures separated by one or more than one diameter of each puncture. Punctures from disc towards anterior area successively slightly enlarged. Genal punctures subequal in size and density to the discal punctures. ***Pronotum*** (Fig. 12B). Margin between anterior and medial-lateral angle almost straight. Medial angle of pronotum rounded. Margin between medial-lateral angle and posterior-lateral angle almost straight. Disc punctures half size to anterior-lateral ones and separated by more than one diameter of each puncture. Basal punctures smaller than anterior-lateral ones and separated by one or more than one diameter of each puncture. ***Hypomera***. With most of punctures incompletely closed and setigerous (as Fig. 3A, white and green arrows). Anterior punctures denser and larger than posterior ones. ***Elytra*** (Fig. 12C). Carina of the ninth interstria almost reaching middle of elytral length. Elytral apex on interstriae III, V–VII with tubercles; interstria IV with a small elevation with the same brightness as the tubercles. Striae I–VII conspicuous. Width of third stria on disc ~1/25 of the distance between striae II and III. Strial punctures almost the same size as interstrial punctures. First stria slightly wider and deeper than second stria. Stria VIII almost inconspicuous apical and laterally. Punctures on interstriae approximately separated by two diameters and the same size or slightly larger than strial punctures. Punctures of the third interstria on disc occupying ~1/13 of the distance between striae II and III. Interstriae with shiny points mixed with the punctures. Points slightly smaller than interstrial punctures. ***Metaventrite*** (Fig. 12D). Disc shallowly excavated, occupying the metaventral basal third. On the middle with a small elevation. Anterolateral areas of metaventral process with punctures with few punctures completely closed and separated by one diameter of each puncture. Anterocentral area of metaventral process with few and small punctures, subequal in size to the discal punctures. Punctures on the lateral lobules closed and separated by one or more than one diameter of each puncture. Dispersed towards the metaventral disc. ***Legs*** (Fig. 13A, B). Protibia regularly broadens toward the apex. Profemora with punctures separated by less than one diameter of each puncture. Mesofemur with punctures separated by ≥ 1× the diameter of each puncture. Mesofemur unarmed on the posterior edge. Metafemur regularly broadens toward the apex. Metafemur with punctures separated by ≥ 1× the diameter of each puncture. Metatibial spur insertion not elongated. Metatibial spur clearly distinct from the metatibia. Internal margin of metatibia with a very small basal carina. ***Abdomen*** (Fig. 12E). Ventrite I expanded posteriorly, expansion reaching the fifth ventrite; on ventrite III, the expansion almost as wide as the distance between mesocoxae. Basal part of the ventrite I, between the metacoxae not modified. ***Pygidium*** (Fig. 13C). Pygidial punctures almost uniform in size and density. Punctures separated by one or less than the diameter of each puncture. Discal punctures, on the center of the pygidium, medium, each puncture occupying ~1/35 of the distance between the lateral pygidial margins. ***Genitalia* (**Fig. 13D–F). Paramera smaller than the phallobase. Apex of paramera with a small denticle in lateral view. Medial area of endophallus with one endophallite.

##### Variation.

The female exhibits the differences described here under the sexual dimorphism section of the redescription of the species group. The medium-sized and major males are unknown.

##### Etymology.

The name *parapseudoabdominale* is an adjective formed from the Greek prefix *pará*-, meaning beside or allied to, and *pseudoabdominale*. This name is given to denote the strong morphological similarity and close affinity to the previously described species *Deltochilum pseudoabdominale* sp. nov.

##### Known distribution

**(Fig. 7B red diamonds)**. Type locality: Venezuela: Aragua: Portachuelo, Rancho Grande, [10°20'51"N], [67°41'15"W], 1100 m. This species is known from Venezuela, Aragua.

##### Remarks.

This species appears to have a restricted distribution. This species is found in sympatry with *D. abdominale*. *Deltochilum parapseudoabdominale* sp. nov. appears to have a more restricted altitudinal range (from 1,000 to probably 2,000 m) than *D. abdominale*.

#### Deltochilum picachos sp. nov.

Taxon classificationAnimaliaColeopteraScarabaeidae

2C2A6A7F-C088-5663-8215-75EED5B6A1D6

https://zoobank.org/F3C3BB2F-B0A8-4610-9D84-9625B9DD9C6E

Figs 1D, 3D, 4F, 7B (purple squares), 14, 15

##### Type material.

***Holotype***: ♂, **Colombia: Caquetá**: San Vicente del Caguán, Alto del río Pato, Fca. Andalucía. PNN Los Picachos, 02°44'N, 74°53'W, 1560 m, 1997.ix.23, Escobar F., T. Exc. H. 14 (IAvH) [IAvH-E-209544]. ***Paratypes* (33 specimens)**: **Colombia: Caquetá**: PNN Los Picachos, 02°48'N, 74°40'W, 1560 m, 1♀ [with aedeagus], 1998.ix, Escobar F., T. Exc. H. (IAvH), PNN Los Picachos, 02°48'N, 74°40'W, 1780 m, 1♂, 1998.ix, Escobar F., T. Exc. H. (IAvH), PNN Los Picachos, 02°48'N, 74°40'W, 1560 m, 1♂, 1998.ix, Escobar F., T. Exc. H. (IAvH), PNN Los Picachos, 02°48'N, 74°40'W, 1780 m, 1♂, 1998.ix, Escobar F., T. Exc. H. (IAvH), PNN Los Picachos. Bosque, 02°47'51'N, 74°51'18"W, 1770 m, 1♀, 1♂, 1997.xi–xii, Escobar F. (IAvH), PNN Los Picachos. Bosque, 02°48'N, 74°40'W, 1780 m, 1♀, 1998.ix, Escobar F., T. Exc. Hum (IAvH), San Vicente del Caguán – PICACHOS. Bosque, [02°48'N], [74°40'W], 1800–2000 m, 3 Unsexed, Nov - 1997, F. Escobar, Hongos (CALT-ECC), San Vicente del Caguán – PICACHOS. Bosque, [02°48'N], [74°40'W], 1780 m, 2 Unsexed, Nov – Dec 1997, F. Escobar, T. 2 (CALT-ECC), San Vicente del Caguán, Alto del río Pato, Fca. Andalucía. PNN Los Picachos, 02°44'N, 74°53'W, 1560 m, 1♂, 1997.ix.23, Escobar F., T. Exc. H. 14 (IAvH), San Vicente del Caguán, Alto del río Pato, Fca. Andalucía. PNN Los Picachos, 02°44'N, 74°53'W, 1780 m, 1♀, 1997.xi, Escobar F., T. Exc. H. 21 (IAvH), San Vicente del Caguán, Alto del río Pato, Fca. Andalucía. PNN Los Picachos, 02°44'N, 74°53'W, 1560 m, 1♀/♂, 1♂/♀, 1997.xi, Escobar F., Trampa con Hongos 20 (IAvH), San Vicente del Caguán, Alto del río Pato, Fca. Andalucía. PNN Los Picachos. Bosque, 02°44'N, 74°53'W, 1780 m, 1 Female/Male#, 1997.xi, Escobar F., T. Exc. H. 21 (IAvH), San Vicente del Caguán, IP Guayabal, Alto del río Pato, Fca. Andalucia. PNN Los Picachos, 02°47'51"N, 74°51'18"W, 1560 m, 1♂ [without aedeagus], 1997.xi–xii, Escobar F., T. Exc. H. (IAvH), San Vicente del Caguán, IP Guayabal, Alto del río Pato, Fca. Andalucia. PNN Los Picachos, 02°48'N, 74°40'W, 1560 m, 1♂, 1998.ix, Escobar F., T. Exc. H. (IAvH), San Vicente del Caguán. Guayabal, PNN Los Picachos, Alto del Río Pato, Fca. Andalucía, 02°47'N, 74°51'W, 1780 m, 8 Unsexed, 1997.xi–xii, Escobar F., Trampa de caída (UPTC-CE), San Vicente del Caguán. Guayabal, PNN Los Picachos, Alto del Río Pato, Fca. Andalucía, 02°44'N, 74°53'W, 1560 m, 2 Unsexed, 1997.xi–xii, Escobar F., Trampa de intercepción de vuelo (UPTC-CE), San Vicente del Caguán. PNN Los Picachos. Bosque, [02°48'N], [74°40'W], 1800 m, 2 Unsexed, 1997.xi, Escobar F., Trampa de intercepción de vuelo (UPTC-CE); **Meta**: Cubarral. F. La Cabaña. B. Montano, 3°49'39"N, 73°53'22.00"W, 1000 m, 2♂, IV/2015, M. Ramírez, PF Exc Hum (CALT-ECC).

##### Diagnosis.

This species is very similar to *D. pseudoabdominale* sp. nov., *D. parapseudoabdominale* sp. nov., and *D. pauxi* sp. nov. by having the striae wide (Figs 10C, 12C, 14C, 16C), and the males with the first ventrite not elevated (Figs 10E, 12E, 14E, 16E) and the mesofemur not modified (Figs 11A, 13A, 15A, 17A). This species can be separated from *D. parapseudoabdominale* sp. nov. by the width of the male first ventrite: at the level of the third ventrite, the width of the first ventrite is narrower than the intermesocoxal distance in this species (Fig. 14E), whereas it is equal to the intermesocoxal distance in *D. parapseudoabdominale* sp. nov. (Fig. 12E). Additionally, they are separated by the punctures on the anterior part of the head: these punctures are slightly larger than the punctures of the head disc in *D. parapseudoabdominale* sp. nov. (Fig. 12A), but are nearly the same size in *D. picachos* sp. nov. (Fig. 14A).

From *D. pauxi* sp. nov., this species can be distinguished by the medial angle of the pronotum, which is rounded in *D. picachos* sp. nov. (Fig. 14B) and salient in *D. pauxi* sp. nov. (Fig. 17G). Furthermore, this species can be separated from *D. pseudoabdominale* sp. nov. by the apical tubercles of the elytra: these tubercles are present on interstriae III, V, and VII in *Deltochilum pseudoabdominale* sp. nov. (Fig. 11C), but are present on interstriae III through VII in *D. picachos* sp. nov. (15C).

##### Description.

Medium-sized male. Size 10 mm. ***Color***. Dark brown. Dark greenish-blue ventrally. ***Head*** (Fig. 14A). Interocular distance 8× the width of one eye. Punctures on frons separated by one or less than one diameter of each puncture and larger in size than discal punctures. Disc punctures separated by two diameters of each puncture. Punctures from disc towards anterior area successively of almost with the same size. Genal punctures subequal in size and density to the discal punctures. ***Pronotum*** (Fig. 14B). Margin between anterior and medial-lateral angle almost straight. Medial angle of pronotum rounded. Margin between medial-lateral angle and posterior-lateral angle subsinuate. Disc punctures three times smaller than the anterior-lateral ones and separated by more than one diameter of each puncture. Basal punctures smaller than anterior-lateral ones and separated by approximately one diameter of each puncture. ***Hypomera***. With most of punctures incompletely closed and setigerous (as Fig. 3A, white and green arrows). Anterior punctures denser and larger than posterior ones. ***Elytra*** (Fig. 14C). Carina of the ninth interstria almost reaching middle of elytral length. Elytral apex on interstriae III–VII with tubercles. Striae I–VII conspicuous. Width of third stria on disc ~1/30 of the distance between striae II and III. Strial punctures ~1.5× wider than the stria. First stria as wide as second stria. Stria VIII conspicuous apical and laterally and reaching carina of the ninth interstria. Punctures on interstriae approximately separated by two or slightly more than two diameters of each puncture and slightly larger than strial punctures. Punctures of the third interstria on disc occupying ~1/12 of the distance between striae II and III. Interstriae with shiny points mixed with the punctures. Points smaller than interstrial punctures, ~3× smaller. ***Metaventrite*** (Figs 3D, 14D). Disc shallowly excavated, occupying the metaventral basal third. On the middle with a small elevation. Anterolateral areas of metaventral process with punctures with few punctures completely closed and separated by less than one diameter of each puncture. Anterocentral area of metaventral process with few and small punctures, slightly larger in size than the discal punctures. Punctures on the lateral lobules not completely closed (some of them) and variable in density, dispersed towards the metaventral disc. ***Legs*** (Fig. 15A, B). Protibia regularly broadens toward the apex. Profemora with punctures separated by one or less than one diameter of each puncture. Mesofemur with punctures separated by ≥ 1× the diameter of each puncture. Mesofemur unarmed on the posterior edge. Metafemur regularly broadens toward the apex. Metafemur with punctures separated by ≥ 1× the diameter of each puncture. Metatibial spur insertion elongated, with the length subequal to the first tarsomere. Metatibial spur clearly distinct from the metatibia. Internal margin of metatibia with a small basal irregular carina, this carina is interrupted by setae giving it the appearance of small tubercles. ***Abdomen*** (Fig. 14E). Ventrite I expanded posteriorly, expansion reaching the fifth ventrite; on ventrite III, the expansion narrower than the distance between mesocoxae. Basal part of the ventrite I, between the metacoxae not modified. ***Pygidium*** (Fig. 15C). Pygidial punctures almost uniform in size and density. Punctures separated by one or less than the diameter of each puncture. Discal punctures, on the center of the pygidium, small, each puncture occupying ~1/40 of the distance between the lateral pygidial margins. ***Genitalia*** (Fig. 15D–F). Paramera smaller than the phallobase. Apex of paramera with a denticle in lateral view. Medial area of endophallus with one endophallite.

##### Variation.

The female exhibits the differences described here under the sexual dimorphism section of the redescription of the species group. In the minor males, the metafemur is regularly broadened toward the apex, and the carina of the internal margin is very small and only slightly reduced in the middle. Metaspur articulate (Fig. 1D). In the major males, the metatibia has a small basal carina. This carina is strongly reduced in the middle, giving the appearance that it is composed of two carinae. Metaspur fused.

##### Etymology.

The name refers to the type locality, PNN Los Picachos, noun in apposition.

##### Known distribution

**(Fig. 7B purple squares)**. Type locality: Colombia: Caquetá: San Vicente del Caguán, Alto del río Pato, Fca. Andalucía. PNN Los Picachos, 02°44'N, 74°53'W, 1560 m. Besides the type locality, this species is known from Meta, Colombia.

##### Remarks.

Three paratype specimens housed in the IAvH-E are chimeras, consisting of the head and pronotum of one individual joined to the meso- and metathorax and abdomen of another. For these specimens, we added labels indicating the sex of the head-pronotum and the sex of the remaining part. In the same collection one male paratype lacked the aedeagus, and one female paratype had an aedeagus glued in a triangle pinned with the specimen.

This species has been collected with traps baited with dung and fungi in forest and montane forest. Its altitudinal range extends from 1,000 to 2,000 m.

#### Deltochilum pauxi sp. nov.

Taxon classificationAnimaliaColeopteraScarabaeidae

28DE431D-4025-5542-986A-53A8C6F2EA00

https://zoobank.org/42BBE1E2-BD03-43ED-9B90-51BF6AFC0EFF

Figs 1C, 3E, 3I, 4G, 7B (yellow triangles), 16, 17

##### Type material.

***Holotype***: ♂, **Colombia: Santander**: Betulia, RN Pauxi pauxi. Bosque, 06°58'14.1"N, 073°26'32.3"W, 1124 m, 06.iii.2019, Ramírez, M., T. Exc.H #PPF2(24 h) (IAvH) [IAvH-E-270466]. ***Paratypes***: **Colombia (6 specimens): Santander**: Betulia, RN Pauxi pauxi. Bosque, 06°58'08.9"N, 073°26'28.0"W, 1295 m, 1♂, 06.iii.2019, Ramírez, M., T. Exc.H #PPF1(24 h) (IAvH), Betulia, RN Pauxi pauxi. Bosque, 06°58'19.6"N, 073°26'35.9"W, 1134 m, 1♀, 06.iii.2019, Ramírez, M., T. Exc.H #PPF3(24 h) (IAvH), Betulia, RN Pauxi pauxi. Bosque, 06°58'08.9"N, 073°26'28.0"W, 1295 m, 1♀, 07.iii.2019, Ramírez, M., T. Exc.H #PPF1(48 h) (IAvH), Betulia, RN Pauxi pauxi. Bosque, 06°58'45.8"N, 073°26'46.4"W, 1096 m, 2♀, 09.iii.2019, Ramírez, M., T. Exc.H #PPF6(96 h) (IAvH), El Carmen de Chucurí, Vereda La Belleza. Bosque subandino Potrero, 6°33'25.8"N, 73°33'8.0"W, 1115 m, 1♂, 2018.2.18–19, Castro M.I., Neita J.C. & Torres E., T. Exc. H. (IAvH).

##### Diagnosis.

**Figure 18.**
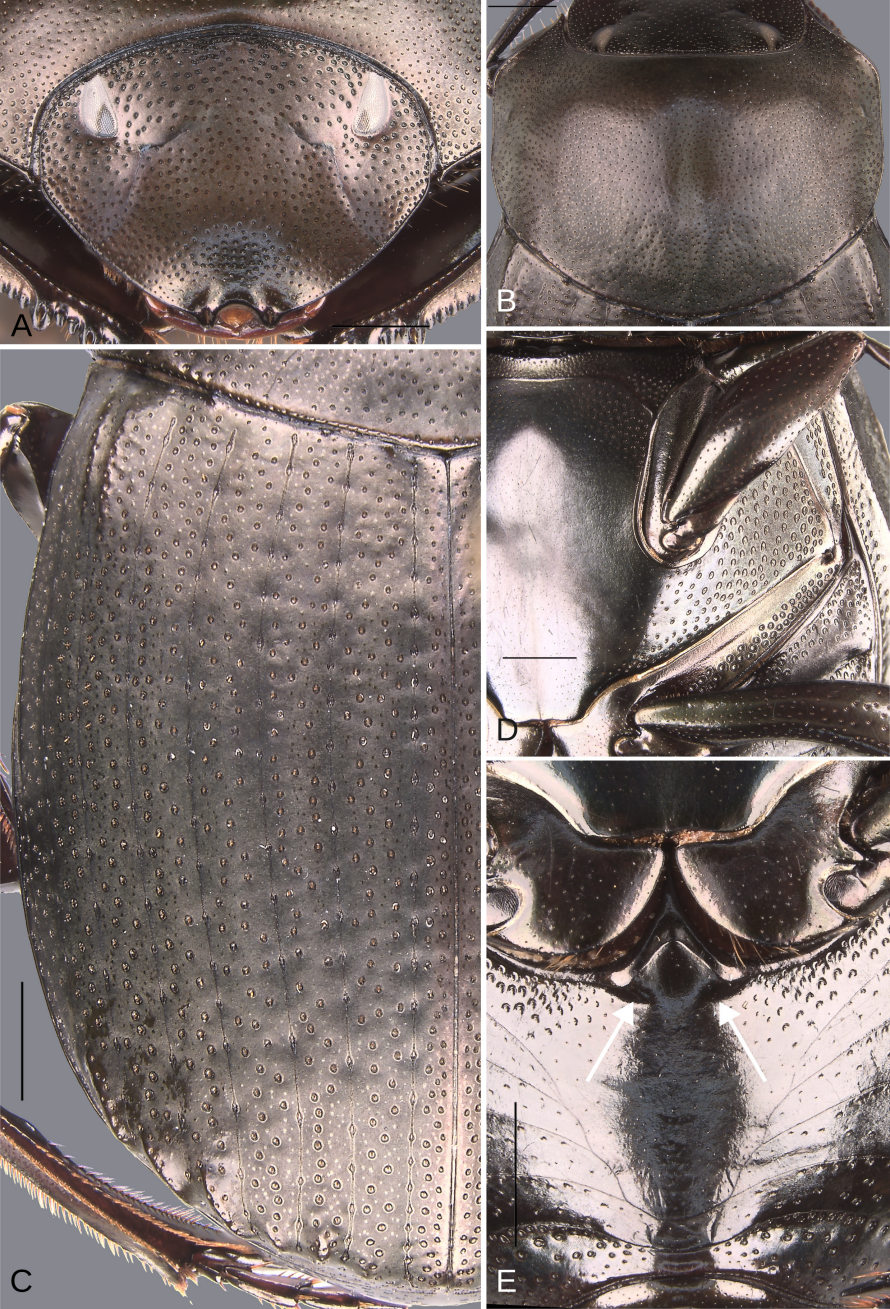
Morphology of *D. altiventris* sp. nov., holotype male. **A**. Head; **B**. Pronotum; **C**. Elytra; **D**. Metaventrite; **E**. Abdomen, ventrites, arrows showing the elevation of the first ventrite. Scale bars: 1 mm.

This species can be distinguished from all other species within the plebejum species group by having the punctures on the anterolateral areas of the metaventral process incompletely closed (Fig. 3E), in combination with a salient medial angle on the pronotum (Fig. 17G) (this angle is rounded in all other species). *Deltochilum altiventris* sp. nov. also exhibits these incompletely closed punctures, but it differs in several ways: the male first ventrite is elevated (Fig. 18E) (not elevated in *D. pauxi* sp. nov. (Fig. 16E)), and the striae are narrow (Fig. 18C) (broad in *D. pauxi* sp. nov. (Fig. 16C)).

##### Description.

Medium-sized male. Size 11 mm. ***Color***. Pale greenish-brown dorsally. Dark greenish-blue ventrally. ***Head*** (Fig. 16A). Interocular distance 9× the width of one eye. Punctures on frons separated by one or less than one diameter of each puncture and slightly larger than the head discal punctures. Disc punctures separated by one or more than one diameter of each puncture. Punctures from disc towards anterior area successively slightly enlarged. Genal punctures more dispersed and slightly larger than the discal punctures. ***Pronotum*** (Fig. 16B). Margin between anterior and medial-lateral angle almost straight. Medial angle of pronotum salient. Margin between medial-lateral angle and posterior-lateral angle subsinuate. Disc punctures half size to anterior-lateral ones and separated by more than one diameter of each puncture. Basal punctures smaller than anterior-lateral ones and separated by approximately one diameter of each puncture. ***Hypomera***. With most of punctures incompletely closed and setigerous (as Fig. 3A, white and green arrows). Anterior punctures denser and larger than posterior ones. ***Elytra*** (Fig. 16C). Carina of the ninth interstria almost reaching middle of elytral length. Elytral apex on interstriae III, V–VII with tubercles; interstriae I and IV with a small elevation with the same brightness as the tubercles. Striae I–VII conspicuous. Width of third stria on disc ~1/20 of the distance between striae II and III. Strial punctures ~2× wider than the stria. First stria slightly wider and deeper than second stria. Stria VIII conspicuous apically and laterally and reaching carina of the ninth interstria. Punctures on interstriae approximately separated by one diameter and almost with the same size or slightly larger than strial punctures. Punctures of the third interstria on disc occupying ~1/9 of the distance between striae II and III. Interstriae with shiny points mixed with the punctures. Points smaller than interstrial punctures, ~4× smaller. ***Metaventrite*** (Figs 3E, 16D). Disc shallowly excavated, occupying the metaventral basal third. Anterolateral areas of metaventral process with punctures not completely closed and separated by less than one diameter of each puncture (Fig. 3E). Anterocentral area of metaventral process with few and small punctures, subequal in size to the discal punctures. Punctures on the lateral lobules most of them not completely closed, separated by less than one diameter of each puncture, dispersed towards the metaventral disc. ***Legs*** (Figs 1C, 3I, 17A, 17B). Protibia regularly broadens toward the apex (Fig. 3I). Profemora with punctures separated by less than one diameter of each puncture. Mesofemur with punctures separated by ≥ 2× the diameter of each puncture. Mesofemur unarmed on the posterior edge. Metafemur regularly broadens toward the apex. Metafemur with punctures separated by ≥ 2× the diameter of each puncture. Metatibial spur insertion elongated, with the length subequal to the basal two tarsomeres (Fig. 1C). Metatibial spur clearly distinct from the metatibia (Fig. 1C). Internal margin of metatibia with a basal irregular carina; this carina is interrupted by setae giving it the appearance of tubercles. Carina reduced in the middle of the metatibial length; following this reduction, the carina rises again as an irregular carina (giving the appearance of having tubercles). ***Abdomen*** (Fig. 16E). Ventrite I expanded posteriorly, expansion reaching the fifth ventrite; on ventrite III, the expansion narrower than the distance between mesocoxae. Basal part of the ventrite I, between the metacoxae not modified. ***Pygidium*** (Fig. 17C). Pygidial punctures almost uniform in size and density. Punctures separated by one or more than the diameter of each puncture. Some lateral-basal punctures not completely closed. Discal punctures, on the center of the pygidium, small, each puncture occupying ~1/40 of the distance between the lateral pygidial margins. ***Genitalia*** (Fig. 17D–F). Paramera smaller than the phallobase. Apex of paramera with a denticle in lateral view. Medial area of endophallus with two endophallites, the right ~5× longer than the other.

##### Variation.

The female exhibits the differences described here under the sexual dimorphism section of the redescription of the species group. The major male (Fig. 17H) has the basal metatibia carina larger. The medial angle of the pronotum is more salient (Fig. 17G). The first ventrite is wider. The metafemur is strongly widened toward the apex. The metaspur is fused. Minor males are unknown.

##### Etymology.

The name refers to the type locality, RN Pauxi pauxi, noun in apposition.

##### Known distribution

**(7B yellow triangles)**. Type locality: Colombia: Santander: Betulia, RN Pauxi pauxi. Bosque, 06°58'14.1"N, 073°26'32.3"W, 1124 m. Besides the type locality, this species is known from El Carmen de Chucurí, Vereda La Belleza, Santander Colombia.

##### Remarks.

This species is currently known from two localities: the type locality and El Carmen de Chucurí, Vereda La Belleza, Santander, Colombia. The type locality represents a forested habitat, while the second appears to be situated in a transition zone between forest and pasture.

#### Deltochilum altiventris sp. nov.

Taxon classificationAnimaliaColeopteraScarabaeidae

34656E6E-64CD-5FE0-BA2F-A05B7F679BCA

https://zoobank.org/FA25867A-AD8D-4641-97CA-1C8BCC4AA079

Figs 3A, 3C, 3H, 4H, 7A (yellow triangles), 18, 19

##### Type material.

***Holotype***: ♂, **Colombia: Cundinamarca**: Medina, Vda. Periquito, Fca. Miramar, Campamento 2. Bosque subandino, Bosque entresacado, 04°30'58.9"N, 73°26'04.2"W, 1212 m, 2018.iii.15–17, Castro M.I., Neita J.C. & Torres E., T. Exc. H. T2T9(48 h) (IAvH) [IAvH-E-203206]. ***Paratypes* (108 specimens)**: **Colombia: Arauca**: Tame. Brisas el Cravo. Bosque, 06°31'25"N, 71°51'57"W, 1500 m, 1♂, 15–19/II/08, F. Alvarado, E.H. (CALT-ECC); **Boyacá**: Cusiana Cerca a Pajarito, 5°23'39"N, 72°41'17"W, 1250 m, 1♀, vi.1997, F. Escobar coll. (CMNC), Cusiana Cerca a Pajarito, 5°23'39"N, 72°41'17"W, 1350 m, 1♂, vi.1997, F. Escobar coll. (CMNC), Cusiana, Cerca a Pajarito. Bosque, 5°23'39"N, 72°41'17"W, 1250 m, 2♀, 3♂, 1997.vi, Escobar F., T. Exc. H. (IAvH), Cusiana, Cerca de Pajarito, Fca. Guayabetal, el paisano. Bosque, 5°17'N, 72°40'W, 1300 m, 1♂, 1997.vi.1, Escobar F., T. Exc. H. 74 (IAvH), Cusiana, Cerca de Pajarito, Fca. Guayabetal, el paisano. Potrero, 5°26'N, 72°41'W, 1000 m, 1♂, 1997.vi.1, Escobar F., T. Exc. H. 73 (IAvH), Pajarito. Corinto. Vereda La Sabana, Corregimiento Corinto, Finca El Mayor. Borde de bosque, 5°24'02.18"N, 72°43'29.20"W, 1542 m, 4 Unsexed, 2/04/2021, Andrés Felipe Morales Alba | David Camilo Martínez Dueñas, Trampa pitfall 4. 24H (UPTC-CE), Paya. Vda. Bocas de Monte. Finca La Piedrota. Bosque, 5°35'21.6"N, 72°27'55.7"W, 1577 m, 1 Unsexed, 9/08/2021, Andrés Felipe Morales Alba | David Camilo Martínez Dueñas, Trampa pitfall 1. 24H (UPTC-CE), Santa María, La Almenará, [4°52'11.29"N], [73°15'21.26"W], 800–1000 m, 1♀, Ago-2008, G. Amat & R. Sarmiento (ICN), Santa María, La Almenará, [4°52'11.29"N], [73°15'21.26"W], 880 m, 1♂, Octubre 2008, G. Amat & Sarmiento R. (ICN), Santa María, Vda. Caño Negro. Bosque, [4°50'57.43"N], [73°16'31.43"W], 1♀, 2♂, 17–19/11/03, Pitfall. M.26 54 (ICN), Santa María. RN La Almenara. Vda. Sta Cecilia. Bosque, 04°52'48.3"N, 073°15'12.5"W, 1191 m, 1♀, 1♂, 16.vii.2018, Martínez, D., T.Ecx.H. #ALF2(72 h) (IAvH), Santa María. Vda. Caño Negro. Fca. Santa María. Bosque, 04°50'22.5"N, 073°17'17.5"W, 1470 m, 2♀, 2♂, 11.vii.2018, Martínez, D., T.Ecx.H. #SMF1R3(72 h) (IAvH), Sta. Maria, Sendero Ecológico, [4°53'N], [73°17'W], 1♀, 2♂, 17–04–97, G. Amat (ICN); Comijoque. Transecto Cusiana. Bosque, 5°25'N, 72°41'W, 2000 m, 1♀, 1997.iv.6, Escobar F. (UPTC-CE), Cusiana cerca de Pajarito. Fca Guayabetal. El Paisano. Bosque, 5°23'N, 72°41'W, 1250 m, 1♀, 1♂, 1997.iii.6, Escobar F. (UPTC-CE), Cusiana cerca de Pajarito. Fca Guayabetal. El Paisano. Bosque, 5°26'N, 72°41'W, 1000 m, 1♀, 1997.v.6, Escobar F. (UPTC-CE), 1♂, 1997.vi.6, Escobar F. (UPTC-CE); **Casanare**: Cusiana. Bosque, 5°23'N, 72°41'W, 1200 m, 2♂, 1997.vi, Escobar F., T. Exc. H. (IAvH), Cusiana. Bosque, 5°23'N, 72°41'W, 1000 m, 1♂, 1997.vi, Escobar F., T. Exc. H. (IAvH), Cusiana. Bosque, 5°23'N, 72°41'W, 1200 m, 1♂, 1997.vi, Escobar F., T. Exc. H. (IAvH), Cusiana. Bosque, 5°23'N, 72°41'W, 1000 m, 1♂, 1997.vi, Escobar F., T. Exc. H. (IAvH), Cusiana. Bosque, 5°23'N, 72°41'W, 1200 m, 2♂, 1997.vi, Escobar F., T. Exc. H. (IAvH), Tauramena. Monserrate Alto. Bosque, 5.069943, -72.855504, 1130 m, 1♀, IV/2018, P. Triviño, PF exc hum (CALT-ECC), Tauramena. Monserrate Alto. Bosque, 5.064737, -72.84851, 1311 m, 1♀, IV/2018, P. Triviño, PF exc hum (CALT-ECC), Tauramena. Monserrate Alto. Bosque, 5.069697, -72.855163, 1120 m, 3♂, IV/2018, P. Triviño, PF exc hum (CALT-ECC), Tauramena. Monserrate Alto. Bosque, 5.069943, -72.855504, 1130 m, 4♂, IV/2018, P. Triviño, PF exc hum (CALT-ECC), Tauramena. Monserrate Alto. Bosque, 5.07047, -72.85743, 1169 m, 1♂, IV/2018, P. Triviño, PF exc hum (CALT-ECC), Tauramena. Monserrate Alto. Bosque, 5.064961, -72.848524, 1271 m, 1♂, IV/2018, P. Triviño, PF exc hum (CALT-ECC), Tauramena. Monserrate Alto. Bosque, 5.064435, -72.848473, 1290 m, 2♂, IV/2018, P. Triviño, PF exc hum (CALT-ECC), Tauramena. Monserrate Alto. Bosque, 5.063548, -72.847425, 1339 m, 3♂, IV/2018, P. Triviño, PF exc hum (CALT-ECC), Tauramena. Vda. El Palmar. Bosque, 5.063548, -72.847425, 1339 m, 1♂, IV/2018, P. Triviño, PF exc hum (CALT-ECC); **Cundinamarca**: Medina, Miralindo, 4°35'33"N, 73°23'17"W, 1500 m, 1♀, 1997.ii-iii, Escobar F., T. Exc. H. (IAvH), Medina, Miralindo, 4°35'33"N, 73°23'17"W, 1250 m, 1♀, 1997.ii-iii, Escobar F., T. Exc. H. (IAvH), Medina, Miralindo, 4°35'33"N, 73°23'17"W, 1000 m, 2♀, 1997.ii-iii, Escobar F., T. Exc. H. (IAvH), Medina, Miralindo, 4°35'33"N, 73°23'17"W, 1500 m, 1♀, 1997.ii-iii, Escobar F., T. Exc. H. (IAvH), Medina, Miralindo, 4°35'33"N, 73°23'17"W, 1000 m, 2♂, 1997.ii-iii, Escobar F., T. Exc. H. (IAvH), Medina, Miralindo, 4°35'33"N, 73°23'17"W, 1500 m, 1♂, 1997.ii-iii, Escobar F., T. Exc. H. (IAvH), Medina, Miralindo, 4°35'33"N, 73°23'17"W, 1000 m, 1♂, 1997.ii-iii, Escobar F., T. Exc. H. (IAvH), Medina, Miralindo, 4°35'33"N, 73°23'17"W, 1250 m, 1♂, 1997.ii-iii, Escobar F., T. Exc. H. (IAvH), Medina, Miralindo, 4°35'33"N, 73°23'17"W, 1000 m, 4♂, 1997.ii-iii, Escobar F., T. Exc. H. (IAvH), Medina, Vda. Periquito, Fca. Miramar, Campamento 2. Bosque subandino, Bosque entresacado, 04°30'58.0"N, 73°26'09.3"W, 1277 m, 1♀, 2♂, 2018.iii.15–16, Castro M.I., Neita J.C. & Torres E., T. Exc. H. T2T6(24 h) (IAvH), Medina, Vda. Periquito, Fca. Miramar, Campamento 2. Bosque subandino, Bosque entresacado, 04°30'58.4"N, 73°26'05.9"W, 1229 m, 1♀, 2018.iii.15–16, Castro M.I., Neita J.C. & Torres E., T. Exc. H. T2T8(24 h) (IAvH), Medina, Vda. Periquito, Fca. Miramar, Campamento 2. Bosque subandino, Bosque entresacado, 04°30'56.8"N, 73°26'11.0"W, 1289 m, 1♂, 2018.iii.15–17, Castro M.I., Neita J.C. & Torres E., T. Exc. H. T2T5(48 h) (IAvH), Medina, Vda. Periquito, Fca. Miramar, Campamento 2. Bosque subandino, Bosque entresacado, 04°30'59.1"N, 73°26'07.8"W, 1246 m, 1♂, 2018.iii.15–17, Castro M.I., Neita J.C. & Torres E., T. Exc. H. T2T7(48 h) (IAvH), Medina, Vda. Periquito, Fca. Miramar, Campamento 2. Bosque subandino, Bosque primario, 04°30'52.4"N, 73°26'32.2"W, 1510 m, 1♀, 2018.iii.14–15, Castro M.I., Neita J.C. & Torres E., T. Exc. H. T1T2(24 h) (IAvH), Medina, Vda. Periquito, Fca. Miramar, Campamento 2. Bosque subandino, Bosque primario, 04°30'54.8"N, 73°26'29.9"W, 1507 m, 1♂, 2018.iii.14–15, Castro M.I., Neita J.C. & Torres E., T. Exc. H. T1T4(24 h) (IAvH), Medina, Vda. Periquito, Fca. Miramar, Campamento 2. Bosque subandino, Bosque primario, 04°30'50.7"N, 73°26'32.6"W, 1515 m, 1♀, 2♂, 2018.iii.14–16, Castro M.I., Neita J.C. & Torres E., T. Exc. H. T1T1(48 h) (IAvH), Medina, Vda. Periquito, Fca. Miramar, Campamento 2. Bosque subandino, Bosque primario, 04°30'53.4"N, 73°26'30.9"W, 1509 m, 1♂, 2018.iii.14–16, Castro M.I., Neita J.C. & Torres E., T. Exc. H. T1T3(48 h) (IAvH), Medina, Vda. Periquito, Fca. Miramar, Campamento 2. Bosque subandino, Bosque primario, 04°30'55.9"N, 73°26'28.5"W, 1527 m, 1♀, 2018.iii.14–16, Castro M.I., Neita J.C. & Torres E., T. Exc. H. T1T5(48 h) (IAvH), Medina. Miralindo, 4°35'33"N, 73°23'17"W, 550 m, 1♂, Fev.Mar/1997, F. Escobar, Ex. Hum. (CALT-ECC), Miralindo, Medina, 4°35'33"N, 73°23'17"W, 1000 m, 1♀, 1♂, ii-iii.1997, F. Escobar coll. (CMNC); **Meta**: Cubarral. F. La Cabaña. B. Montano, 3°49'39"N, 73°53'22.00"W, 1000 m, 1♀, 1♀, IV/2015, M. Ramírez, PF Exc Hum (CALT-ECC), Cubarral. F. La Cabaña. B. Montano, 3°49'39"N, 73°53'22.00"W, 530 m, 2♀, IV/2015, M. Ramírez, PF Exc Hum (CALT-ECC), Cubarral. F. La Cabaña. B. Montano, 3°49'39"N, 73°53'22.00"W, 1000 m, 2♀, IV/2015, M. Ramírez, PF Exc Hum (CALT-ECC), Cubarral. F. La Cabaña. B. Montano, 3°49'39"N, 73°53'22.00"W, 530 m, 2 h?#, IV/2015, M. Ramírez, PF Exc Hum (CALT-ECC), Cubarral. F. La Cabaña. B. Montano, 3°49'39"N, 73°53'22.00"W, 1000 m, 4♂, IV/2015, M. Ramírez, PF Exc Hum (CALT-ECC), Cubarral. F. La Cabaña. B. Montano, 3°49'39"N, 73°53'22.00"W, 530 m, 1♂, IV/2015, M. Ramírez, PF Exc Hum (CALT-ECC), Cubarral. F. La Cabaña. B. Montano, 3°49'39"N, 73°53'22.00"W, 1000 m, 2♂, IV/2015, M. Ramírez, PF Exc Hum (CALT-ECC), Cubarral. F. La Cabaña. B. Montano, 3°49'39"N, 73°53'22.00"W, 530 m, 1♂, IV/2015, M. Ramírez, PF Exc Hum (CALT-ECC), Cubarral. F. La Cabaña. B. Montano, 3°49'39"N, 73°53'22.00"W, 1000 m, 1♂, IV/2015, M. Ramírez, PF Exc Hum (CALT-ECC), Cubarral. F. La Cabaña. B. Montano, 3°49'39"N, 73°53'22.00"W, 530 m, 1♂, IV/2015, M. Ramírez, PF Exc Hum (CALT-ECC), Cubarral. Vda. Alto Vergel. RN Las Palmeras. Bosque premontano, 03°50'39.4"N, 73°53'15.7"W, 1500 m, 2♂, Abril 2015, M. Ramírez, Exc H PF (CALT-ECC).

##### Diagnosis.

**Figure 19.**
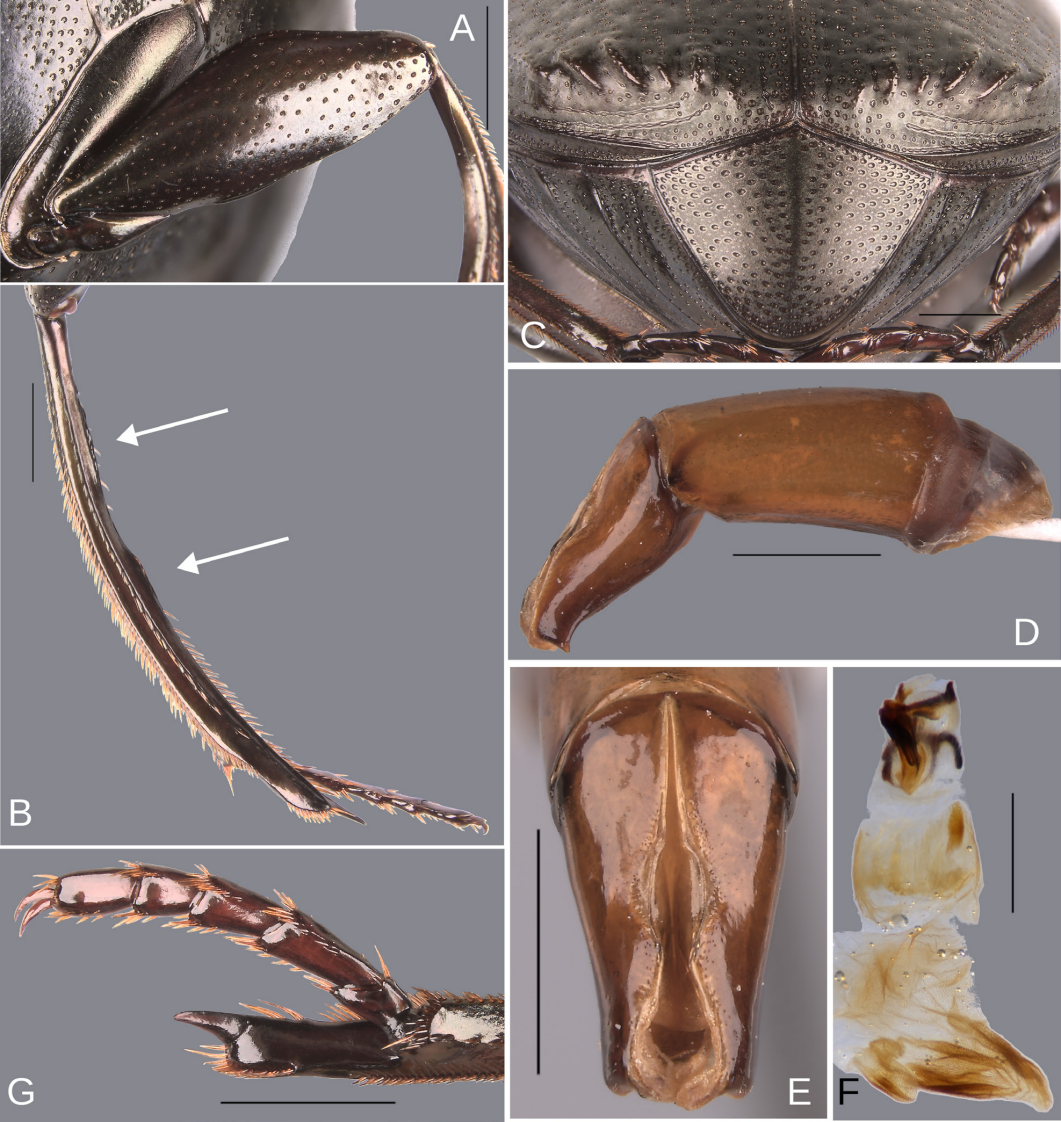
Morphology of *D. altiventris* sp. nov., holotype male. **A**. Mesofemur; **B**. Metatibia, arrows showing the internal carina; **C**. Pygidium; **D**. Aedeagus, lateral view; **E**. Parameres, dorsal view; **F**. Endophallus; **G**. Apex of the metatibia. Scale bars: 1 mm.

This species can be distinguished from all other species within the plebejum species group by having incompletely closed punctures on the anterolateral areas of the metaventral process (as Fig. 3E), in combination with the elevated first ventrite in males (Fig. 18E) and the distribution (II–VII) of the tubercles on the elytral apex (Fig. 19C).

*Deltochilum pauxi* sp. nov. also has these punctures incompletely closed, but its first ventrite is not elevated (Fig. 16E), it has a salient medial angle on the pronotum (Fig. 17G) (which is rounded in *D. altiventris* sp. nov. (Fig. 18B)), and the striae are broad (Fig. 16C) (narrow in *D. altiventris* sp. nov. (Fig. 18C)).

The narrow striae and the elevated first ventrite in males may lead to confusion with *D. plebejum* Balthasar, 1939 (Fig. 24C) and *D. nobile* sp. nov. (Fig. 22C), but *D. altiventris* sp. nov. can be differentiated by the apical tubercles of the elytra: on interstriae III, V, and VII in those species (Figs 23C, 24E) versus interstriae II–VII in *D. altiventris* sp. nov. (Fig. 19C).

##### Description.

Major male. Size 10.30 mm. ***Color***. Dark brown dorsally. Dark greenish-blue ventrally. ***Head*** (Fig. 18A). Interocular distance 8× the width of one eye. Punctures on frons separated by one or more than one diameter of each puncture and subequal or larger in size than discal punctures. Disc punctures separated by two or more diameter of each puncture. Punctures from disc towards anterior area successively slightly enlarged. Genal punctures almost with the same density and slightly larger than the discal punctures. ***Pronotum*** (Fig. 18B). Margin between anterior and medial-lateral angle almost straight. Medial angle of pronotum rounded. Margin between medial-lateral angle and posterior-lateral angle almost straight. Disc punctures three times smaller than the anterior-lateral ones and separated by more than one diameter of each puncture. Basal punctures smaller than anterior-lateral ones and separated by one or more diameters of each puncture. ***Hypomera***. With most of punctures incompletely closed and setigerous (Fig. 3A, white and green arrows). Anterior punctures denser and larger than posterior ones. ***Elytra*** (Fig. 18C). Carina of the ninth interstria almost reaching middle of elytral length. Elytral apex on interstriae II–VII with tubercles. Striae I–VII conspicuous. Width of third stria on disc ~1/16 of the distance between striae II and III. Strial punctures ~2× wider than the striae. First stria slightly wider and deeper than second stria. Stria VIII conspicuous apically, laterally almost inconspicuous. Punctures on interstriae approximately separated by two diameters and almost with the same size as strial punctures. Punctures of the third interstria on disc occupying ~1/9 of the distance between striae II and III. Interstriae with shiny points mixed with the punctures. Points smaller than interstrial punctures, ~4× smaller. ***Metaventrite*** (Fig. 18D). Disc shallowly excavated, occupying the metaventral basal third. Anterolateral areas of metaventral process with punctures not completely closed and separated by one diameter of each puncture. Anterocentral area of metaventral process with few and small punctures, slightly larger in size than the discal punctures. Punctures on the lateral lobules most of them not completely closed, separated by less than one diameter of each puncture, dispersed towards the metaventral disc. ***Legs*** (Fig. 19A, B). Protibia strongly broadens toward the apex. Profemora with punctures separated by one or less than one diameter of each puncture. Mesofemur with punctures separated by ≥ 2× the diameter of each puncture. Mesofemur unarmed on the posterior edge. Metafemur strongly constricted basally and broadens toward the apex. Metafemur with punctures separated by ≥ 2× the diameter of each puncture. Metatibial spur insertion elongated, with the length subequal to the basal two tasomeres. Metatibial spur fused with the metatibia. Internal margin of metatibia with a basal irregular carina. Carina reduced in the middle of the metatibial length; following this reduction, the carina rises again as an irregular carina (giving the appearance of having two carinae). ***Abdomen*** (Fig. 18E). Ventrite I expanded posteriorly, expansion reaching the fourth ventrite; on ventrite III, the expansion narrower than the distance between mesocoxae. Basal part of the ventrite I, between the metacoxae elevated. ***Pygidium*** (Fig. 19C). Pygidial punctures almost uniform in size and density. Punctures separated by one or less than the diameter of each puncture. Some lateral-basal punctures not completely closed. Discal punctures, on the center of the pygidium, medium sized, each puncture occupying ~1/36 of the distance between the lateral pygidial margins. ***Genitalia*** (Fig. 19D–F). Paramera smaller than the phallobase. Apex of paramera with a denticle in lateral view. Medial area of endophallus with one endophallite.

##### Variation.

The female exhibits the differences described here under the sexual dimorphism section of the redescription of the species group. In females the punctures of the anterior-lateral areas of the metaventral process extend more towards the anterior-central area than in males. Some specimens have the punctures on the anterior part of the head more dispersed and/or smaller than the holotype; others a little larger and denser. In worn specimens, the striae are very difficult to see.

The minor males have the metatibial basal carina smaller. The first ventrite is less elevated. The metafemur is less broadened towards the apex. Metaspur articulate. In medium-sized males, the metatibial basal carina is larger, the first ventrite is more elevated and the metafemur is more broadened towards the apex than those of the minor males. Metaspur articulate. In the largest major males, the metatibial spur insertion is broadened apically, and the spur has a hook-like (as Fig. 1A) and the protibia is broader in the apical half (Fig. 3H).

##### Etymology.

The name, *altiventris*, is an adjective formed from the Latin *altus* meaning elevated, and the Latin *ventris* meaning abdomen. The name refers to the distinctly elevated first ventrite characteristic (although not exclusive) of this species.

##### Known distribution

**(Fig. 7A yellow triangles)**. Type locality: Colombia: Cundinamarca: Medina, Vda [Vereda]. Periquito, Fca [Finca]. Miramar, Campamento 2. Bosque subandino, Bosque entresacado, 04°30'58.9"N, 73°26'04.2"W, 1212 m. This species also is known from Colombia, Arauca, Boyacá, Casanare, and Meta.

##### Remarks.

This species has been collected in forest, sub-montane and montane forest, primary and secondary forest and one specimen was collected in pasture. This species is one of the members of the plebejum species group that is widely distributed, possessing a broad altitudinal range from 500 to probably 1,500 m.

#### Deltochilum ventripuncturatus sp. nov.

Taxon classificationAnimaliaColeopteraScarabaeidae

24AC097D-44A9-5F37-8D91-5C0917D419C9

https://zoobank.org/AC7E0827-CCBF-4CCE-8D7C-545E96AD3611

Figs 1A, 4I, 7A (purple squares), 20, 21

Deltochilum (Deltohyboma) abdominalis Martínez 1954: 47 (cited from Colombia, error).

##### Type material.

***Holotype***: ♂, **Colombia: Magdalena**: Santa Marta, Minca, Cerro Kennedy. Bosque, 11°05'04.9"N, 074°01'54.8"W, 1908 m, 2018.i.29, Martínez D., T. Exc. H. SLF4(72 h) (IAvH) [IAvH-E-209518]. ***Paratypes* (28 specimens)**: **Colombia: Mag. [Magdalena]**: Sra.Nev.Sta. Marta Cerro Kennedy – Vda. Minas, *B. de* sombra con cafetal, [11°4'54.58"N], [74°1'16.32"W], 1280 m, 2♀, 1♂, Enero 5/1999, J. A. Noriega, *T. pitfall* Ex. Hum. Gradiente Altit. (CEMT), Sra.Nev.Sta. Marta Cerro Kennedy – Vda. Minas, *B. maduro* con cafetal, [11°4'54.58"N], [74°1'16.32"W], 1670 m, 1♀, Enero 2/1999, J. A. Noriega, *T. pitfall* Ex. Hum. Gradiente Altit. (CALT-ECC); **Magdalena**: Santa Marta, Minca, Cerro Kennedy. Bosque, 11°05'04.9"N, 074°01'54.8"W, 1908 m, 5♀, 5♂, 2018.i.29, Martínez D., T. Exc. H. SLF4(72 h) (IAvH), Santa Marta, Minca, Cerro Kennedy. Bosque, 11°05'01.6"N, 074°02'00.5"W, 1914 m, 1♂, 2018.i.29, Martínez D., T. Exc. H. SLF5(48 h) (IAvH), 1♀, 1♂, 2018.i.29, Martínez D., T. Exc. H. SLF5(72 h) (IAvH), Santa Marta, Minca, Cerro Kennedy. Bosque, 11°05'04.9"N, 074°01'54.8"W, 1908 m, 2♀, 3♂, 2018.i.30, Martínez D., T. Exc. H. SLF4(96 h) (IAvH), Santa Marta, Minca, Cerro Kennedy. Bosque, 11°05'01.6"N, 074°02'00.5"W, 1914 m, 3♂, 2018.i.30, Martínez D., T. Exc. H. SLF5(96 h) (IAvH), Santa Marta, Minca, Cerro Kennedy. Bosque, 11°05'08.4"N, 074°02'00.5"W, 1973 m, 1♀, 1♂, 2018.i.30, Martínez D., T. Exc. H. SLF6(96 h) (CBUMAG), Santa Marta, Mts. Vista Nieva, [11°4'22.69"N], [74°4'56.66"W], 5400 ft.m, 1♂, 26.vi.1920, F Saige & M coll. (BDGC).

##### Diagnosis.

**Figure 20.**
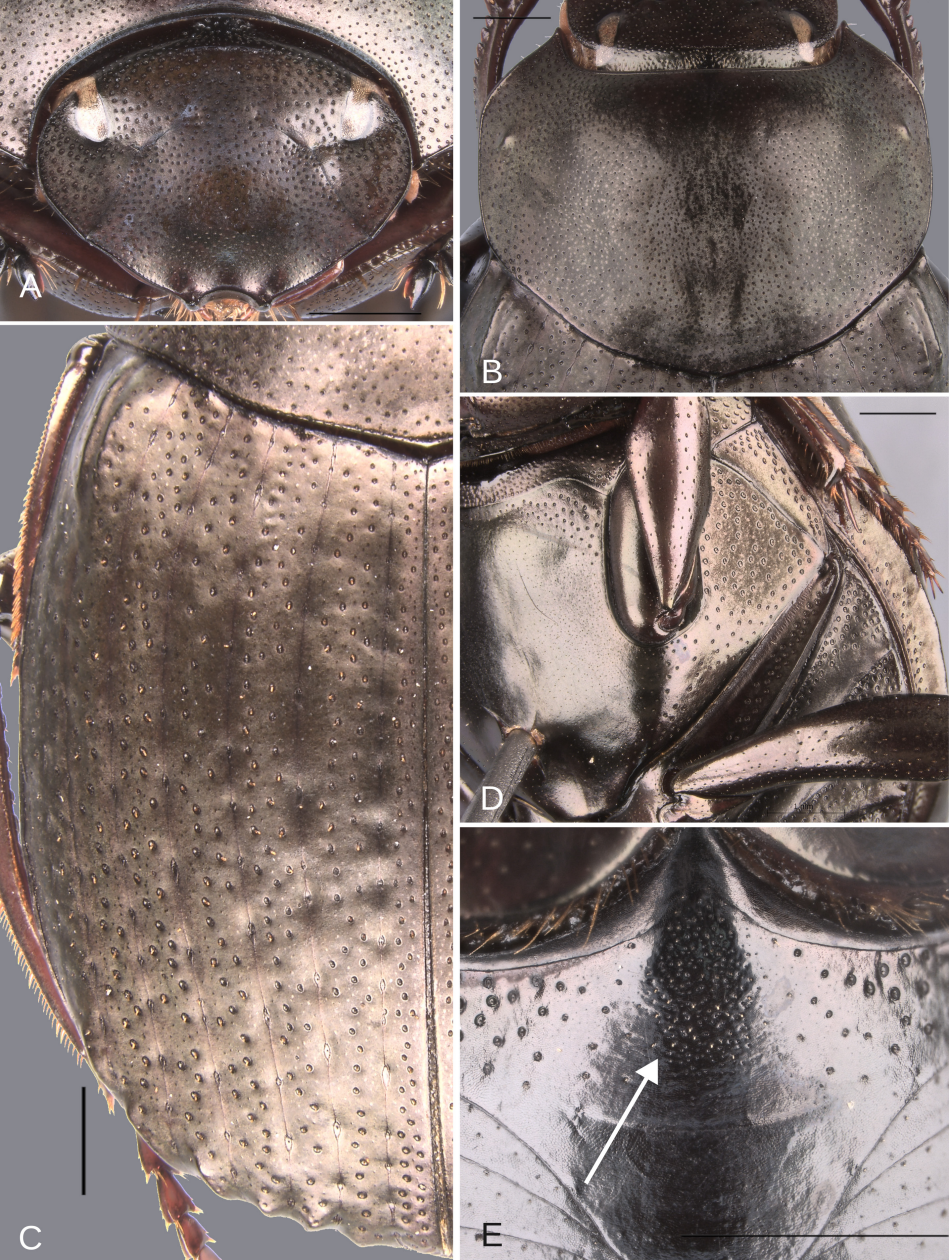
Morphology of *D. ventripuncturatus* sp. nov., holotype male. **A**. Head; **B**. Pronotum; **C**. Elytra; **D**. Metaventrite; **E**. Abdomen, ventrites, arrow showing the small punctures of the first ventrite. Scale bars: 1 mm.

**Figure 21.**
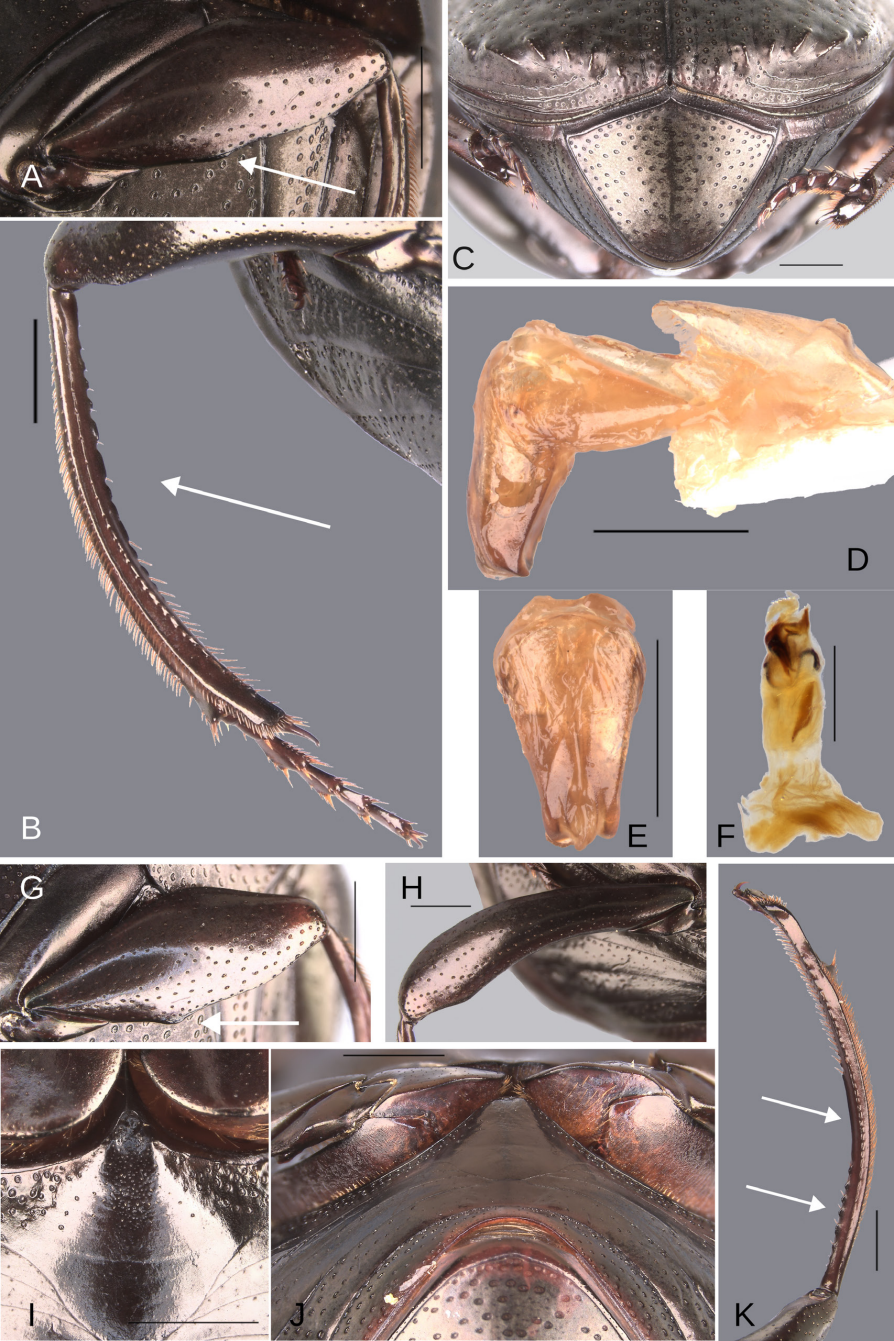
Morphology of *D. ventripuncturatus* sp. nov., **A–F**. Holotype male, **G–H**. Paratype, major male. **A**. Mesofemur, arrow showing the protuberance; **B**. Metatibia, arrow showing the internal carina; **C**. Pygidium; **D**. Aedeagus, lateral view; **E**. Parameres, dorsal view; **F**. Endophallus; **G**. Mesofemur, arrow showing the protuberance; **H**. Metafemur; **I**. Abdomen, first ventrite; **J**. Abdomen, showing the elevation of the first ventrite; **K**. Metatibia, arrows showing the internal carina. Scale bars: 1 mm.

This species can be distinguished from all other species within the plebejum species group by the unique combination of characters in males: the mesofemur possesses a basal protuberance (Fig. 21A, G), the first ventrite is elevated and with small punctures (Figs 20E, 21I, 21J). Also, it can be separated from all other species of the plebejum species group by the head punctures, the punctures on the anterior part of the head are smaller than the punctures of the head disc (Fig. 20A), in the other species of plebejum species group, the punctures on the anterior part of the head are equal to or larger than the punctures of the head disc.

##### Description.

Medium-sized male. Size 10.70 mm. ***Color***. Pale greenish-brown dorsally. Dark copperish-green ventrally. ***Head*** (Fig. 20A). Interocular distance 8× the width of one eye. Punctures on frons separated by one or less than one diameter of each puncture and subequal in size than discal punctures. Disc punctures separated by two diameters of each puncture. Punctures from disc towards anterior area successively smaller. Genal punctures larger and more dispersed than the discal punctures. ***Pronotum*** (Fig. 20B). Margin between anterior and medial-lateral angle almost straight. Medial angle of pronotum rounded. Margin between medial-lateral angle and posterior-lateral angle almost straight. Disc punctures slightly smaller than anterior-lateral ones and separated by more than one diameter of each puncture. Basal punctures smaller than anterior-lateral ones and separated by approximately two diameters of each puncture. ***Hypomera***. With most of punctures incompletely closed and setigerous (as Fig. 3A, white and green arrows). Anterior punctures denser and larger than posterior ones. ***Elytra*** (Fig. 20C). Carina of the ninth interstria almost reaching middle of elytral length. Elytral apex on interstriae II–VII with tubercles. Striae I–VII almost inconspicuous. Width of third stria on disc ~1/30 of the distance between striae II and III. Strial punctures variable in size, ≥ 3× wider than the stria. First stria as wider as second stria. Stria VIII conspicuous apically and laterally and reaching carina of the ninth interstria. Punctures on interstriae approximately separated by two diameters and almost with the same size or smaller than strial punctures. Punctures of the third interstria on disc occupying ~1/15 of the distance between striae II and III. Interstriae with shiny points mixed with the punctures. Points 4× smaller than interstrial punctures. ***Metaventrite*** (Fig. 20D). Disc shallowly excavated, occupying the metaventral basal third. Anterolateral areas of metaventral process with punctures completely closed and separated by one diameter of each puncture. Anterocentral area of metaventral process with few and small punctures, larger in size to the discal punctures. Punctures on the lateral lobules not completely closed (some of them) and variable in density, separated by one or more than one diameter of each puncture. ***Legs*** (Fig. 21A, B). Protibia strongly broadens toward the apex. Profemora with punctures separated by one or less than one diameter of each puncture. Mesofemur with punctures separated by ≥ 2× the diameter of each puncture. Mesofemur with a basal protuberance in the basal third of the posterior edge. Metafemur regularly broadens toward the apex. Metafemur with punctures separated by ≥ 2× the diameter of each puncture. Metatibial spur insertion elongated, with the length subequal to the first tarsomere. Metatibial spur clearly distinct from the metatibia. Internal margin of metatibia with large and irregular tubercles reaching the basal half of the metatibial length. ***Abdomen*** (Fig. 20E). Ventrite I expanded posteriorly, expansion reaching the fourth ventrite; on ventrite III, the expansion narrower than the distance between mesocoxae. Basal part of the ventrite I, between the metacoxae elevated and with small and dense punctures. ***Pygidium*** (Fig. 21C). Pygidial punctures almost uniform in size and density. Punctures separated by several times the diameter of each puncture. Some lateral-basal punctures not completely closed. Discal punctures on the center of the pygidium, medium, each puncture occupying ~1/33 of the distance between the lateral pygidial margins. ***Genitalia*** (Fig. 21D–F). Paramera smaller than the phallobase. Apex of paramera with a denticle in lateral view. Medial area of endophallus with a large endophallite.

##### Variation.

The female exhibits the differences described here under the sexual dimorphism section of the redescription of the species group. Furthermore, in females, the medial part of the first ventrite bears punctures, but these are the same size as the other punctures of that ventrite. These medial punctures are not as small and dense as those found in males. The clypeal teeth are closer to one another in females than in males.

In minor males, the mesofemur bears a smaller basal protuberance. The tubercles on the metatibia are smaller and more regular in size. The first ventrite is less elevated. The small punctures of the first ventrite are less conspicuous. Metaspur articulate. In major males, the mesofemur bears strongest basal protuberance (Fig. 21G). The metatibia is more strongly curved (Fig. 21K). The metatibial basal carina is strongly interrupted by setae, giving it the appearance of large tubercles (Fig. 21K, white arrow). This carina is significantly reduced in the middle of the metatibial length; following this reduction, the carina rises again (giving the appearance of having both tubercles and carina) (Fig. 21K, white arrow). The first ventrite is more elevated (Fig. 21I–J). Metaspur fused. In the largest males, the metatibial spur insertion is broadened apically, and the spur has a hook-like shape (Fig. 1A).

##### Etymology.

The name, *ventripuncturatus*, is an adjective derived from the Latin *ventris* meaning abdomen and *puncturatus* meaning punctured. The name refers to the presence of small and dense punctures on the first abdominal ventrite, a unique diagnostic character of this species.

##### Known distribution

**(Fig. 7A purple squares)**. Type locality: Colombia: Magdalena: Santa Marta, Minca, Cerro Kennedy. Bosque, 11°05'04.9"N, 074°01'54.8"W, 1908 m. This species is only known from the Sierra Nevada de Santa Marta, Colombia.

##### Remarks.

We were able to examine a male specimen deposited in the BDGC collection that shares the exact collecting data (locality, date, collector) of the two specimens reported by Martínez (1954) from Santa Marta, Colombia, under the name *D. abdominale*. Upon revision, this specimen does not correspond to *D. abdominale*; it is definitively *D. ventripuncturatus* sp. nov. This finding suggests that the historical record of *D. abdominale* from Santa Marta, Colombia, is likely based on a misidentified specimen of *D. ventripuncturatus* sp. nov.

This species appears to be restricted to the Sierra Nevada de Santa Marta (Colombia). It has been collected from 1,200 to ~ 2,000 m.

#### Deltochilum nobile sp. nov.

Taxon classificationAnimaliaColeopteraScarabaeidae

19CA152D-FF17-55AA-8E5E-D04CF976C30E

https://zoobank.org/EF097B66-F696-4D51-BB81-3914710A082E

Figs 4J, 7A (white star), 22, 23

##### Type material.

***Holotype***: ♂, **Colombia: Cesar**: Agustín Codazzi, Buenos Aires. Bosque, 10.022979, -73.105296, 1200–1300 m, 14–15.VII.2025, Chaverra, D., T. Exc.H #G2P2 (ICN). ***Paratypes* (22 specimens)**: **Colombia: Cesar**: Agustín Codazzi, Buenos Aires. Bosque, 10.022979, -73.105296, 1200–1300 m, 3♀, 12♂, 14–15.VII.2025, Chaverra, D., T. Exc.H #G2P2 (ICN); **La Guajira**: Barrancas. Las Pavas, 10°50'18.5"N, 72°40'15"W, 1700 m, 1♂, 16/05/2016, A. Redondo, T. Exc.H (CBUMAG), Barrancas. Las Pavas, 10°50'26.8"N, 72°40'10.7"W, 1708 m, 1♂, 16/05/2016, A. Redondo, T. Caida-Carroña (IAvH), Barrancas. Surimena, 10°51'17.3"N, 72°40'40.4"W, 1053 m, 1♀, 30/10/2015, A. Redondo (CBUMAG), Barrancas. Surimena, 10°51'10.7"N, 72°40'15.3"W, 1027 m, 1♀, 17/05/2016, A. Redondo, T. Caida-Carroña (IAvH), Urumita. [Vereda Los Medios] Serranía del Perijá. Tres Picos, [10°27'37.2"N], [72°57'47.8"W], [1800 m], 2♀, 1♂, 5/11/2009, J. Cárdenas (UPTC-CE).

##### Diagnosis.

**Figure 22.**
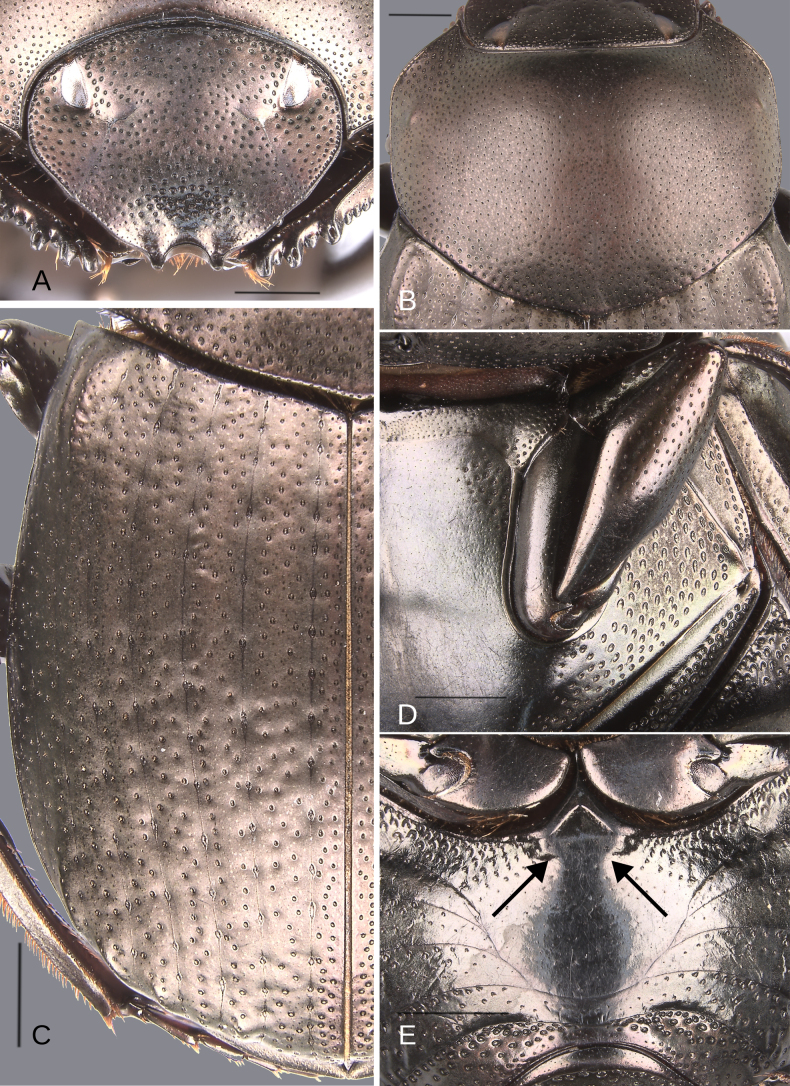
Morphology of *D. nobile* sp. nov., holotype male**. A**. Head; **B**. Pronotum; **C**. Elytra; **D**. Metaventrite; **E**. Abdomen, ventrites, arrow showing the elevation of the first ventrite. Scale bars: 1 mm.

**Figure 23.**
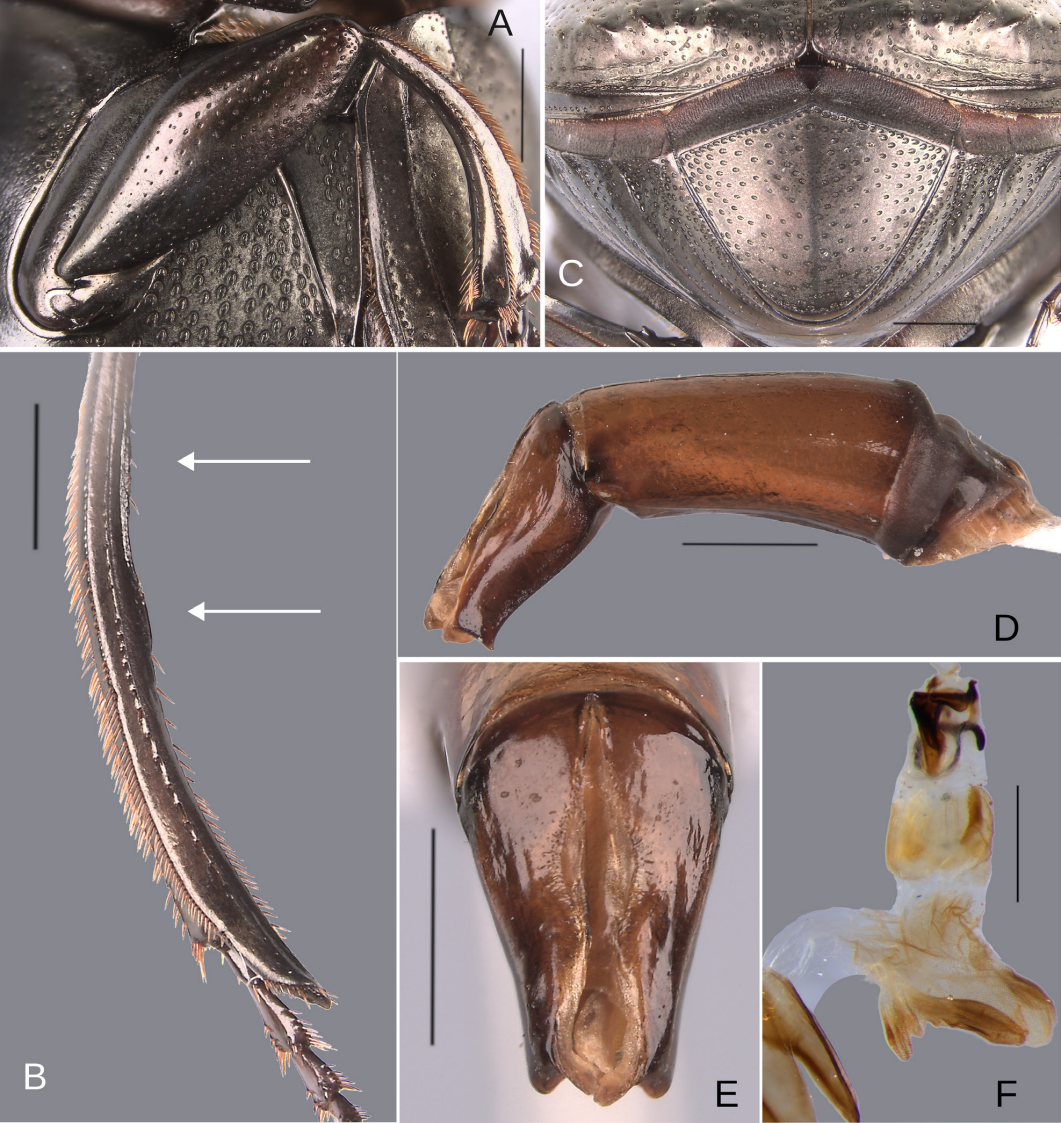
Morphology of *D. nobile* sp. nov., holotype male. **A**. Mesofemur; **B**. Metatibia, arrows showing the internal carina; **C**. Pygidium; **D**. Aedeagus, lateral view; **E**. Parameres, dorsal view; **F**. Endophallus. Scale bars: 1 mm.

This species can be distinguished from all other species within the plebejum species group by having the largest punctures on the anterior part of the head, which are larger than the punctures on the head disc (Fig. 22A). In *D. altiventris* sp. nov., these punctures are only slightly larger than the punctures of the head disc (Fig. 18A). These species can also be separated by the apical tubercles of the elytra: tubercles on interstriae III, V, and VII in *D. nobile* sp. nov. (Fig. 23C) versus interstriae II–VII in *D. altiventris* sp. nov. (Fig. 19C).

##### Description.

Medium-sized male. Size 10.40 mm. ***Color***. Pale greenish-brown dorsally. Dark greenish-blue ventrally. ***Head*** (Fig. 22A). Interocular distance 8× the width of one eye. Punctures on frons separated by one or less than one diameter of each puncture and subequal in size to head discal punctures. Disc punctures separated by one or more than one diameter of each puncture. Punctures from disc towards anterior area successively larger. Genal punctures larger and denser than the discal punctures. ***Pronotum*** (Fig. 22B). Margin between anterior and medial-lateral angle almost straight. Medial angle of pronotum rounded. Margin between medial-lateral angle and posterior-lateral angle almost straight. Disc punctures half size to anterior-lateral ones and separated by more than one diameter of each puncture. Basal punctures smaller than anterior-lateral ones and separated by approximately two diameters of each puncture. ***Hypomera***. With ocellated and setigerous punctures on the anterior part (as Fig. 3B, white arrow). Most of punctures incompletely closed posteriorly and without setae. Anterior punctures denser and larger than posterior ones. ***Elytra*** (Fig. 22C). Carina of the ninth interstria almost reaching middle of elytral length. Elytral apex on interstriae III, V–VII with tubercles. Striae I–VII almost inconspicuous. Width of third stria on disc ~1/30 of the distance between striae II and III. Strial punctures ~2× wider than the stria. First stria slightly wider and deeper than second stria. Stria VIII conspicuous apically and laterally and reaching carina of the ninth interstria. Punctures on interstriae approximately separated by two diameters, and are almost the same size or smaller than strial punctures. Punctures of the third interstria on disc occupying ~1/10 of the distance between striae II and III. Interstriae with shiny points mixed with the punctures. Points several times smaller than interstrial punctures. ***Metaventrite*** (Fig. 22D). Disc shallowly excavated, occupying the metaventral basal third. Anterolateral areas of metaventral process with punctures completely closed and separated by less than one diameter of each puncture. Anterocentral area of metaventral process with few and small punctures, subequal in size to the disc punctures. Punctures on the lateral lobules not completely closed (some of them) and variable in density, dispersed towards the metaventral disc. ***Legs*** (Fig. 23A, B). Protibia regularly broadens toward the apex. Profemora with punctures separated by less than one diameter of each puncture. Mesofemur with punctures separated by ≥ 2× the diameter of each puncture. Mesofemur unarmed on the posterior edge. Metafemur strongly constricted basally and broadens toward the apex. Metafemur with punctures separated by ≥ 2× the diameter of each puncture. Metatibial spur insertion almost with the length of the two basal tarsomeres. Metatibial spur clearly distinct from the metatibia. Internal margin of metatibia with a basal irregular carina. Carina reduced in the middle of the metatibial length; following this reduction, the carina rises again as an irregular carina (giving the appearance of having two carinae). ***Abdomen*** (Fig. 22E). Ventrite I expanded posteriorly, expansion reaching the fifth ventrite; on ventrite III, the expansion narrower than the distance between mesocoxae. Basal part of the ventrite I, between the metacoxae elevated. ***Pygidium*** (Fig. 23C). Pygidial punctures almost uniform in size and density. Punctures separated by one or less than the diameter of each puncture. Some lateral-basal punctures are not completely closed. Discal punctures on the center of the pygidium large, each puncture occupying ~1/25 of the distance between the lateral pygidial margins. ***Genitalia*** (Fig. 23D–F). Paramera smaller than the phallobase. Apex of paramera with a denticle in lateral view. Medial area of endophallus with one endophallite.

##### Variation.

The female exhibits the differences described here under the sexual dimorphism section of the redescription of the species group. Minor males have the first ventrite less elevated. The metafemur is regularly broadened toward the apex, and the carina of the internal margin of the metatibia is very small and only slightly reduced in the middle. Metaspur articulate. In major males, the first ventrite is more elevated, the metafemur is strongly broadened towards the apex, and the carina of the internal margin of the metatibia is stronger. In largest males, the metatibial spur insertion is broadened apically, and the spur has a hook-like (as Fig. 1A)

##### Etymology.

The name *nobile* is derived from the Latin *nobilis* meaning noble. The name is given in direct contrast to the closely related and morphologically similar species, *Deltochilum plebejum* (meaning common or plebeian).

##### Known distribution

**(7A white star)**. Type locality: Colombia: Cesar: Agustín Codazzi, Buenos Aires. Bosque, 10.022979, -73.105296, 1200–1300 m. This species is known from Cesar and La Guajira, Colombia.

#### Deltochilum plebejum

Taxon classificationAnimaliaColeopteraScarabaeidae

Balthasar, 1939

BEE11780-8E48-5F1C-9475-0BFFE7AF4509

Figs 4K–L, 7A (red cross), 24, 25

Deltochilum (s. str.) plebejum Balthasar, 1939: 14.
Deltochilum plebejum : Génier 2001: 6.

##### Type material examined.

***Lectotype* (here designated)** ♂ **Venezuela**: Maracaibo Basin, [10.455446]?, [-71.787763]? E. Gevaerts/ coll Valck Lucassen/Museum Leiden verz FT Valck Lucassen/ *Deltochilum* (s. str.) plebejum sp. nov. Dr. V. Balthasar det/ [red label] TYPUS/ [red label] LECTOTYPE [male symbol] *Deltochilum plebejum* Balth. des. F.Z. Vaz-de-Mello, 2014 (RMNH). ***Paralectotype***: ♀ **Venezuela**: Maracaibo Basin, [10.455446]?, [-71.787763]? E. Gevaerts/ coll Valck Lucassen/ ex coll. V. Balthasar National Museum Prague, Czech Republic/ *Deltochilum* spec.? det. Boucomont/ [red label] TYPUS/ [green label] plebejum m./ [yellow label] PARALECTOTYPE [male symbol] *Deltochilum plebejum* Balth. des. F.Z. Vaz-de-Mello, 2014 (NMPC).

##### Diagnosis.

**Figure 24.**
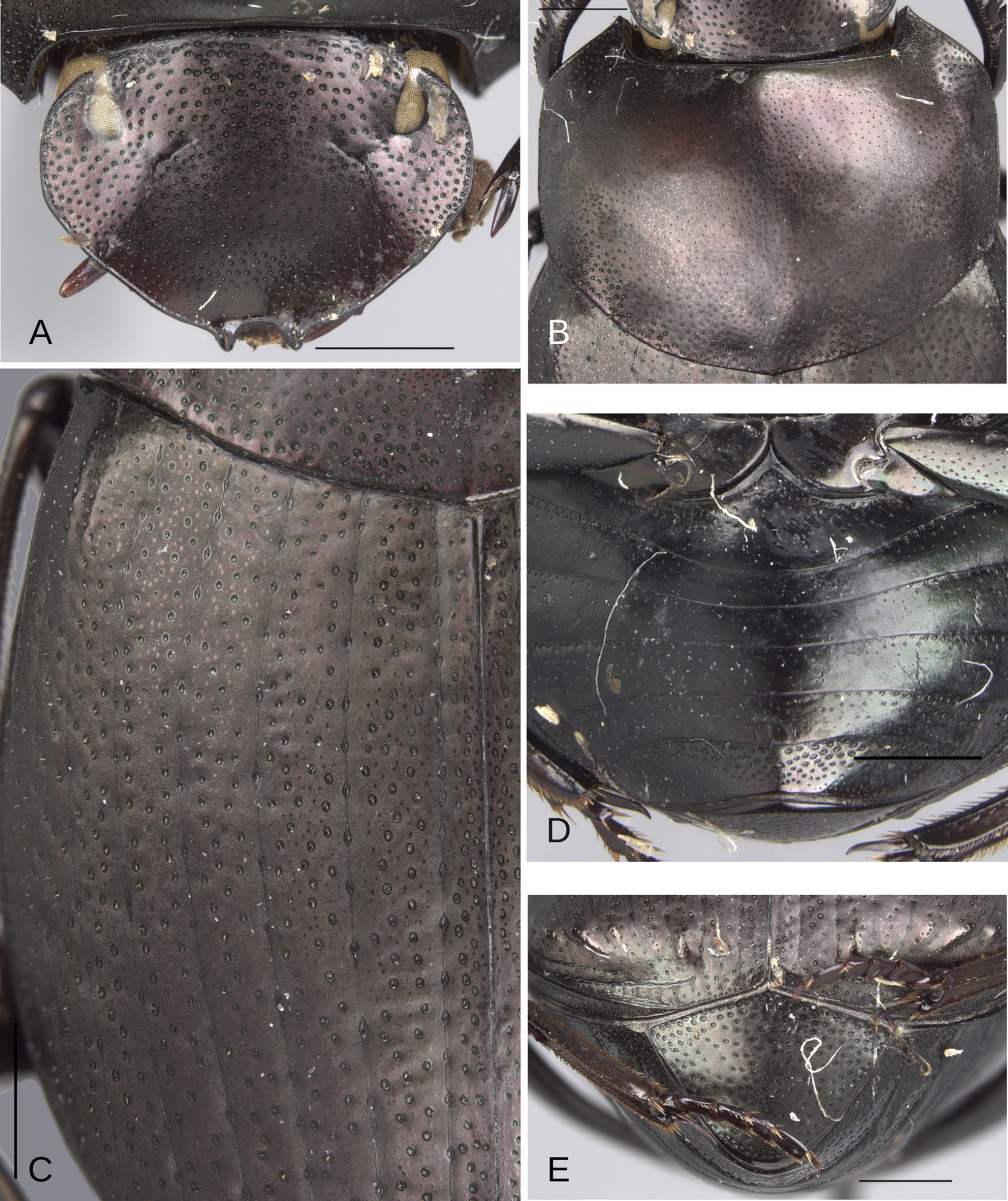
Morphology of *Deltochilum plebejum* Balthasar, 1939, paralectotype female. **A**. Head; **B**. Pronotum; **C**. Elytra; **D**. Abdomen; **E**. Pygidium. Scale bars: 1 mm.

This species shares the narrow striae and the apical tubercles of the elytra (on interstriae III, V, and VII) with *D. nobile* sp. nov. (Figs 23C, 24E), but it can be differentiated by the head punctures. The punctures on the anterior part of the head are nearly the same size as the head disc punctures in *D. plebejum* (Fig. 24A), whereas they are larger in *D. nobile* sp. nov. (Fig. 22A).

##### Redescription.

Female. Size 9.50 mm. ***Color***. Pale reddish-brown dorsally. Dark greenish-blue ventrally. ***Head*** (Fig. 24A). Interocular distance eight and a half times the width of one eye. Punctures on frons separated by less than one diameter of each puncture and slightly larger than the head discal punctures. Disc punctures separated by one or more than one diameter of each puncture. Punctures from disc towards anterior area successively almost the same size. Genal punctures subequal in size and density to the discal punctures. ***Pronotum*** (Fig. 24B). Margin between anterior and medial-lateral angle almost straight. Medial angle of pronotum rounded. Margin between medial-lateral angle and posterior-lateral angle almost straight. Disc punctures half size to anterior-lateral ones and separated by more than one diameter of each puncture. Basal punctures smaller than anterior-lateral ones and separated by approximately one diameter of each puncture. ***Hypomera***. With ocellated and setigerous punctures on the anterior part (as Fig. 3B, white arrow). Most of punctures incompletely closed posteriorly and with setae (as Fig. 3B, green arrow). Anterior punctures denser and larger than posterior ones. ***Elytra*** (Fig. 24C). Carina of the ninth interstria not reaching middle of elytral length. Elytral apex on interstriae III, V–VII with tubercles. Striae I–VII almost inconspicuous. Width of third stria on disc ~1/40 of the distance between striae II and III. Strial punctures variable in size, ≥ 2× wider than the stria. First stria slightly wider and deeper than second stria. Stria VIII conspicuous apically and laterally and reaching carina of the ninth interstria. Punctures on interstriae approximately separated by two diameters. Punctures of the third interstria on disc occupying ~1/7 of the distance between striae II and III. Interstriae with shiny points mixed with the punctures. Points smaller than interstrial punctures, ~4× smaller. ***Metaventrite***. Anterolateral areas of metaventral process with punctures not completely closed and separated by less than one diameter of each puncture. Anterocentral area of metaventral process with few and small punctures, slightly larger in size than the discal punctures. Punctures on the lateral lobules most of them not completely closed, separated by less than one diameter of each puncture. ***Legs***. Protibia regularly broadens toward the apex. Profemora with punctures separated by less than one diameter of each puncture. Mesofemur with punctures separated by ≥ 2× the diameter of each puncture. Mesofemur with unarmed on the posterior edge. Metafemur with punctures separated by ≥ 1× the diameter of each puncture. Metatibial spur insertion not elongated. Metatibial spur clearly distinct from the metatibia. ***Abdomen*** (Fig. 24D). Ventrite I regular, not expanded posteriorly. ***Pygidium*** (Fig. 24E). Pygidial punctures variable in size and density. Punctures separated by one or less than the diameter of each puncture on the disc, more disperse towards the sides. Discal punctures, on the center of the pygidium, small, each puncture occupying ~1/38 of the distance between the lateral pygidial margins.

##### Variation.

**Figure 25.**
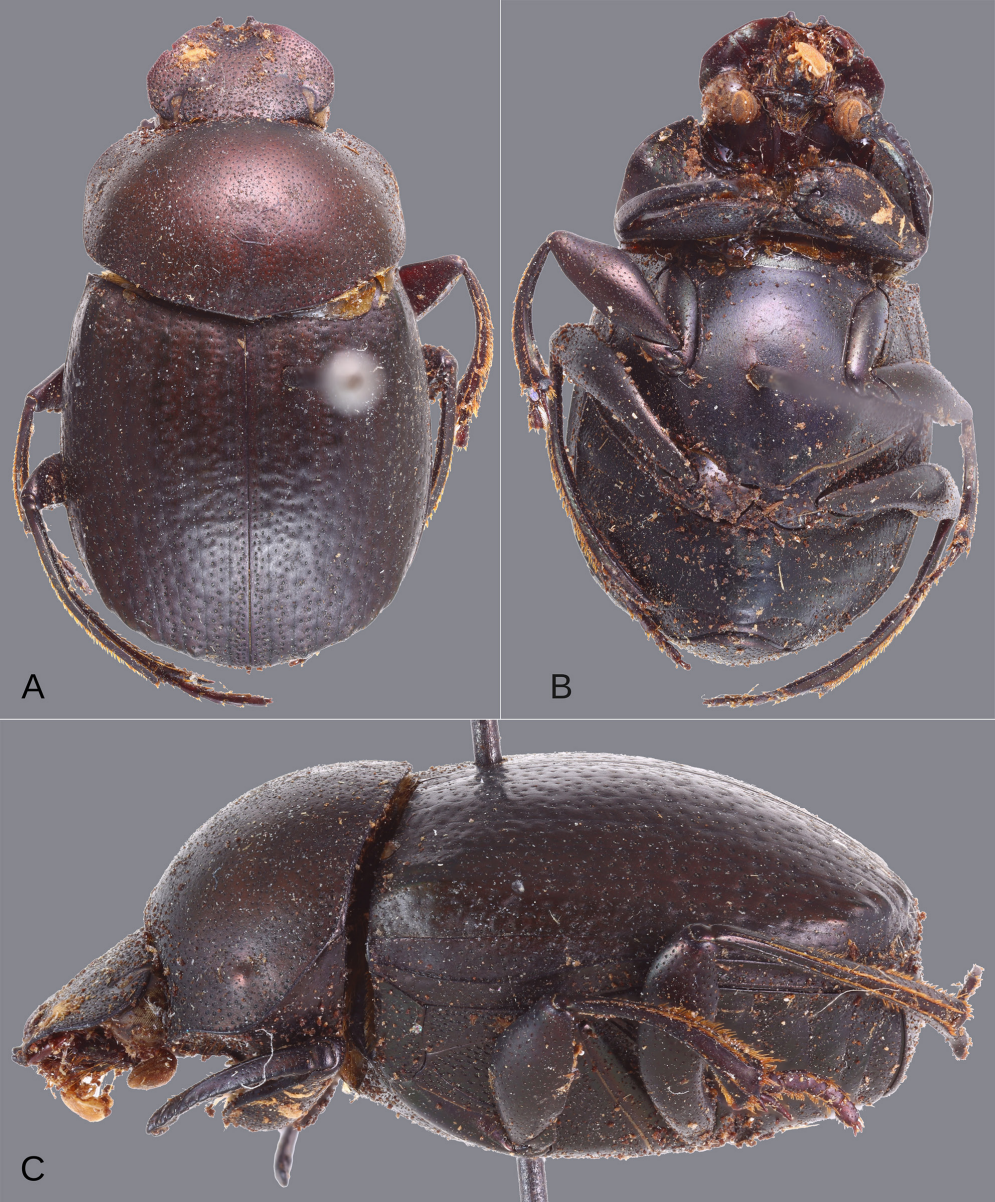
Morphology of *Deltochilum plebejum* Balthasar, 1939, lectotype male. **A**. Dorsal habitus; **B**. Ventral view; **C**. Lateral view.

Medium-sized male: Protibia regularly broadens toward the apex. Mesofemur unarmed on the posterior edge. Metafemur strongly constricted basally and broadens toward the apex. Metatibial spur insertion elongated, with the length subequal to the basal two tarsomeres. Metatibial spur fused with the metatibia. Internal margin of metatibia with a basal irregular carina. Carina reduced in the middle of the metatibial length; following this reduction, the carina rises again as a irregular carina (giving the appearance of having two carinae). Ventrite I expanded posteriorly, expansion reaching the fourth ventrite; on ventrite III, the expansion narrower than the distance between mesocoxae. Apparently, the basal part of the ventrite I, between the metacoxae is elevated (Fig. 25B–C).

##### Known distribution

(7A red cross). This species is only known from the type locality: the Maracaibo Basin, Venezuela. As this locality is highly unspecific, the red cross is intended only as a graphical representation of the possible distribution and, for this reason, is highlighted with a question mark (?).

##### Remarks.

The lectotype is designated following the Article 74 of the International Code of Zoological Nomenclature (ICZN 1999) and the recommendations herein, in order to stabilize the nomenclature.

This species is known to us only from the type series: a male lectotype (RMNH) and a female paralectotype (NMPC) (although this specimen bears a male symbol in the paralectotype label). The now-designated lectotype was originally housed in the Valck Lucassen collection in Vorden (Holland) (Balthasar 1939). After Mr. F. T. Valck Lucassen’s death in September 1939, his collection was transported to the Leiden Museum (De Jong 1970). The paralectotype was originally housed in Balthasar collection and is now housed in NMPC (Génier 2001). The paralectotype was reviewed in person and the redescription here was made using that specimen. The lectotype was examined by a photographs kindly provided by Dr. Fernando Vaz de Mello (Fig. 4K) and Dr. Olivier Montreuil (Fig. 25) It is not clearly visible, but apparently the first ventrite is elevated (Fig. 25B, C).

We consider this species to be closely related to *D. nobile* sp. nov. based on the size of the striae, the size and density of the interstrial punctures, the number and distribution of the tubercles on the elytral apex, the shape of the internal carina of the metatibia, the apparent elevation of the first ventrite, and the distribution.

### Keys to species of the plebejum species group

**Table:** 

**Key for major males**		
1	Basal part of ventrite I, between metacoxae modified (elevated or elevated and with small punctures) (Figs 8E, 9H, 18E, 21I–J, 22E)	**2**
–	Basal part of ventrite I, between metacoxae not modified (Figs 5E, 10E, 13E, 14E, 16E)	**6**
2	Basal part of ventrite I, between the metacoxae elevated and with small punctures (Fig. 21I–J)	*** Deltochilum ventripuncturatus* sp. nov**.
–	Basal part of ventrite I, between the metacoxae elevated, without small punctures (Figs 8E, 9H, 18E, 22E)	**3**
3	Mesofemur with basal denticle on the posterior edge (Fig. 9G)	*** Deltochilum tyba* sp. nov**.
–	Mesofemur without a basal denticle, at most with a basal sinuation (Figs 19A, 23A)	**4**
4	Punctures on anterior part of head larger than head discal punctures (Fig. 22A)	*** Deltochilum nobile* sp. nov**.
–	Punctures on anterior part of head slightly larger than head discal punctures (Figs 18A, 24A, 25A)	**5**
5	Tubercles at elytral apex on interstriae II–VII (Fig. 19C)	*** Deltochilum altiventris* sp. nov**.
–	Tubercles at elytral apex on interstriae III, V–VII (Figs 24E, 25C)	*** Deltochilum plebejum* Balthasar, 1939**
6	Mesofemur with basal denticle on posterior edge (Fig. 6G)	*** Deltochilum abdominale* Martínez, 1947**
–	Mesofemur without a basal denticle, at most with a basal sinuation (Figs 11A, 13A, 15A, 17A)	**7**
7	Medial-lateral angle of pronotum salient (Fig. 17G). Punctures on anterolateral areas of the metaventral process not fully closed and occupying a significant portion of these areas (Figs 3E, 16D)	*** Deltochilum pauxi* sp. nov**.
–	Medial-lateral angle of pronotum rounded (Figs 10B, 12B, 14B). Punctures on anterolateral areas of the metaventral process fully closed and occupying a small portion of these areas (Figs 3D, 10D, 12D, 14D)	**8**
8	Width of the expansion of ventrite I (on ventrite III), almost as wide as the distance between mesocoxae (Fig. 13E)	*** Deltochilum parapseudoabdominale* sp. nov**.
–	Width of the expansion of ventrite I (on ventrite III), narrower than the distance between mesocoxae (Figs 10E, 14E)	**9**
9	Width of the expansion of ventrite I (on ventrite III), narrower than the distance between mesocoxae (Fig. 10E). Tubercles at elytral apex on the interstriae III, V–VII (Fig. 11C)	*** Deltochilum pseudoabdominale* sp. nov**.
–	Width of the expansion of ventrite I (on ventrite III), much narrower than the distance between mesocoxae (Fig. 14E). Tubercles at elytral apex on the interstriae III–VII (Fig. 15C)	*** Deltochilum picachos* sp. nov**.
**Key for minor or medium-sized males**		
1	Basal part of ventrite I, between the metacoxae modified (elevated or elevated and with small punctures) (Figs 8E, 9H, 18E, 21I–J, 22E, 25B–C)	**2**
–	Basal part of ventrite I, between the metacoxae not modified (Figs 5E, 10E, 13E, 14E, 16E)	**6**
2	Basal part of ventrite I, between the metacoxae elevated and with small punctures (Fig. 21I–J)	*** Deltochilum ventripuncturatus* sp. nov**.
–	Basal part of ventrite I, between the metacoxae elevated, but without small punctures (Figs 8E, 9H, 18E, 22E, 25B–C)	**3**
3	Punctures on anterior part of head larger than head discal punctures (Fig. 22A)	*** Deltochilum nobile* sp. nov**.
–	Punctures on anterior part of head with almost the same size or slightly larger than head discal punctures (Figs 8A, 18A, 24A, 25A)	**4**
4	Internal carina of metatibia, forming small tubercles (Fig. 9B)	*** Deltochilum tyba* sp. nov**.
–	Internal carina of metatibia elevated or slightly elevated, that elevation normally reduced on the middle of the metatibial length, giving the appearance of two carinae (Figs 19B, 25A–B)	**5**
5	Tubercles at elytral apex on interstriae II–VII (Fig. 19C)	*** Deltochilum altiventris* sp. nov**.
–	Tubercles at elytral apex on interstriae III, V–VII (Figs 24E, 25C)	*** Deltochilum plebejum* Balthasar, 1939**
6	Mesofemur with basal denticle on posterior edge (Fig. 6A). Striae almost inconspicuous, width of third stria on disc ~1/55 of the distance between striae II and III (Fig. 5C)	*** Deltochilum abdominale* Martínez, 1947**
–	Mesofemur without a basal denticle, at most with a basal sinuation (Figs 11A, 13A, 15A, 17A). Striae conspicuous, width of third stria on disc ~1/20–1/30 of the distance between striae II and III (Figs 10C, 12C, 14C, 16C)	**7**
7	Medial-lateral angle of pronotum salient (Fig. 16B). Punctures on anterolateral areas of metaventral process not fully closed and occupying a significant portion of these areas (Figs 3E, 16D)	*** Deltochilum pauxi* sp. nov**.
–	Medial-lateral angle of pronotum rounded (Figs 10B, 12B, 14B). Punctures on anterolateral areas of metaventral process fully closed and occupying a small portion of these areas (Figs 3D, 10D, 12D, 14D)	**8**
8	Width of the expansion of ventrite I (on ventrite III), almost as wide as the distance between mesocoxae (Fig. 13E)	*** Deltochilum parapseudoabdominale* sp. nov**.
–	Width of the expansion of ventrite I (on ventrite III), narrower than the distance between mesocoxae (Figs 10E, 14E)	**9**
9	Width of the expansion of ventrite I (on ventrite III), narrower than the distance between mesocoxae (Fig. 10E). Tubercles at elytral apex on interstriae III, V–VII (Fig. 11C)	*** Deltochilum pseudoabdominale* sp. nov**.
–	Width of the expansion of ventrite I (on ventrite III), much narrower than the distance between mesocoxae (Fig. 14E). Tubercles at elytral apex on interstriae III–VII (Fig. 15C)	*** Deltochilum picachos* sp. nov**.
**Key for females (also applicable to males)**		
1	Interstrial shiny points subequal in size to interstrial punctures (Fig. 5C)	*** Deltochilum abdominale* Martínez, 1947**
–	Interstrial shiny points smaller than interstrial punctures (Figs 8C, 10C, 12C, 14C, 16C, 18C, 20C, 22C, 24C)	**2**
2	Punctures on anterolateral areas of metaventral process not fully closed (Figs 3E, 16D, 18D, 25B)	**3**
–	Punctures on anterolateral areas of metaventral process fully closed (Figs 3D, 8D, 10D, 12D, 14D, 20D, 22D)	**5**
3	Medial-lateral angle of pronotum salient (Figs 16B, 17G). Interstrial punctures separated by one diameter of each puncture (Fig. 16C)	*** Deltochilum pauxi* sp. nov**.
–	Medial-lateral angle of pronotum rounded (Figs 18B, 24B, 25A). Interstrial punctures separated by approximately two diameters of each puncture (Figs 18C, 24C)	**4**
4	Tubercles at elytral apex on interstriae II–VII (Fig. 19C)	*** Deltochilum altiventris* sp. nov**.
–	Tubercles at elytral apex on interstriae III, V–VII (Figs 24E, 25C)	*** Deltochilum plebejum* Balthasar, 1939**
5	Striae thin, in some species almost inconspicuous (Figs 8C, 20C, 22C)	**6**
–	Striae wide, striae always conspicuous (Figs 10C, 12C, 14C)	**8**
6	Punctures on anterior part of head smaller than head discal punctures (Fig. 20A)	*** Deltochilum ventripuncturatus* sp. nov**.
–	Punctures on anterior part of the head from almost same size to larger than head discal punctures (Figs 8A, 22A)	**7**
7	Punctures on the anterior part of head larger than head discal punctures (Fig. 22A)	*** Deltochilum nobile* sp. nov**.
–	Punctures on anterior part of head slightly larger than head discal punctures (Fig. 8A)	*** Deltochilum tyba* sp. nov**.
8	Tubercles at elytral apex on interstriae III–VII (Fig. 15C)	*** Deltochilum picachos* sp. nov**.
–	Tubercles at elytral apex on interstriae III, V–VII (Figs 11C, 13C)	**9**
9	Punctures on anterolateral areas of metaventral process separated by less than one diameter of each puncture (Fig. 10D). Punctures on anterior part of head with almost the same size than head discal punctures (Fig. 10A)	*** Deltochilum pseudoabdominale* sp. nov**.
–	Punctures on anterolateral areas of metaventral process separated by one diameter of each puncture (Fig. 12D). Punctures on anterior part of head slightly larger than head discal punctures (Fig. 12A)	*** Deltochilum parapseudoabdominale* sp. nov**.

## Supplementary Material

XML Treatment for Deltochilum abdominale

XML Treatment for Deltochilum tyba

XML Treatment for Deltochilum pseudoabdominale

XML Treatment for Deltochilum parapseudoabdominale

XML Treatment for Deltochilum picachos

XML Treatment for Deltochilum pauxi

XML Treatment for Deltochilum altiventris

XML Treatment for Deltochilum ventripuncturatus

XML Treatment for Deltochilum nobile

XML Treatment for Deltochilum plebejum

## Additional information

### Artificial Intelligence (AI) use

The authors accept full responsibility for the content of the manuscript, including the disclosure of any use of AI.

No AI tools were used in the preparation of this manuscript.

### Author ORCIDs

A. González-Alvarado https://orcid.org/0000-0002-0343-3704

J.C. Neita https://orcid.org/0000-0003-2998-2063

### Data availability

All of the data that support the findings of this study are available in the main text.
